# Supplementary material for: Tumor-Induced IL-6 Reprograms Host Metabolism to Suppress Anti-tumor Immunity
Source: Cell Metab. 2016 Nov 8;24(5):672–84. doi: 10.1016/j.cmet.2016.10.010 (PMC5106372; doi:10.1016/j.cmet.2016.10.010)
Supplement: Document S1. Supplemental Experimental Procedures, Figures S1–S7, and Table S1 [file mmc1.pdf]

Cell Metabolism, Volume *24*

Supplemental Information

Tumor-Induced IL-6 Reprograms Host Metabolism to Suppress Anti-tumor Immunity

Thomas R. Flint, Tobias Janowitz, Claire M. Connell, Edward W. Roberts, Alice E. Denton, Anthony P. Coll, Duncan I. Jodrell, and Douglas T. Fearon

Figure S1

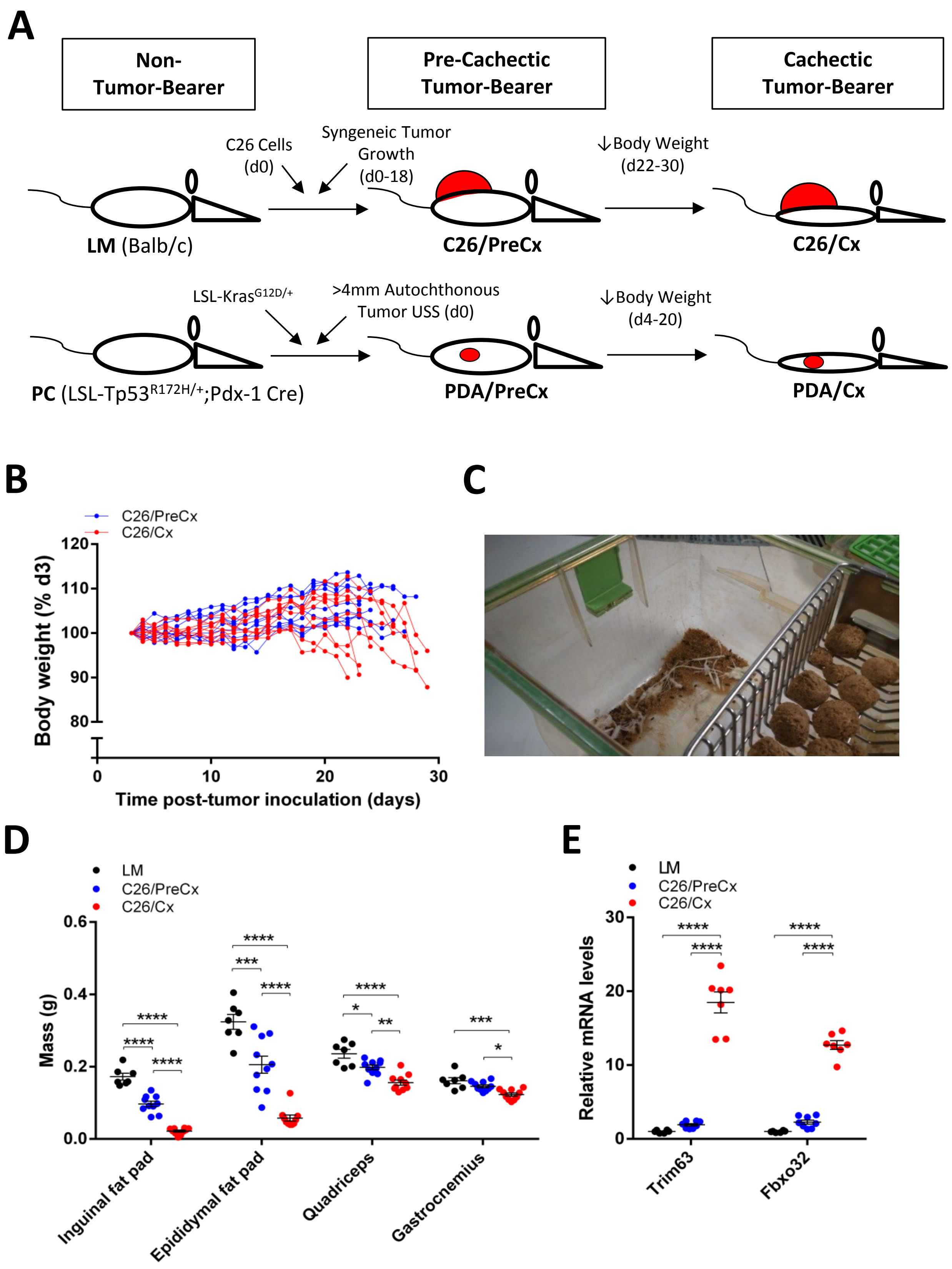
A
Non-Tumor-Bearer
Pre-Cachectic Tumor-Bearer
Cachectic Tumor-Bearer
C26 Cells (d0)
Syngeneic Tumor Growth (d0-18)
↓Body Weight (d22-30)
LM (Balb/c)
C26/PreCx
C26/Cx
LSL-Kras$^{G12D/+}$
>4mm Autochthonous Tumor USS (d0)
↓Body Weight (d4-20)
PC (LSL-Tp53$^{R172H/+}$;Pdx-1 Cre)
PDA/PreCx
PDA/Cx
B
C26/PreCx
C26/Cx
Body weight (% d3)
Time post-tumor inoculation (days)
C
D
LM
C26/PreCx
C26/Cx
Mass (g)
Inguinal fat pad
Epididymal fat pad
Quadriceps
Gastrocnemius
E
LM
C26/PreCx
C26/Cx
Relative mRNA levels
Trim63
Fbxo32

Figure S1

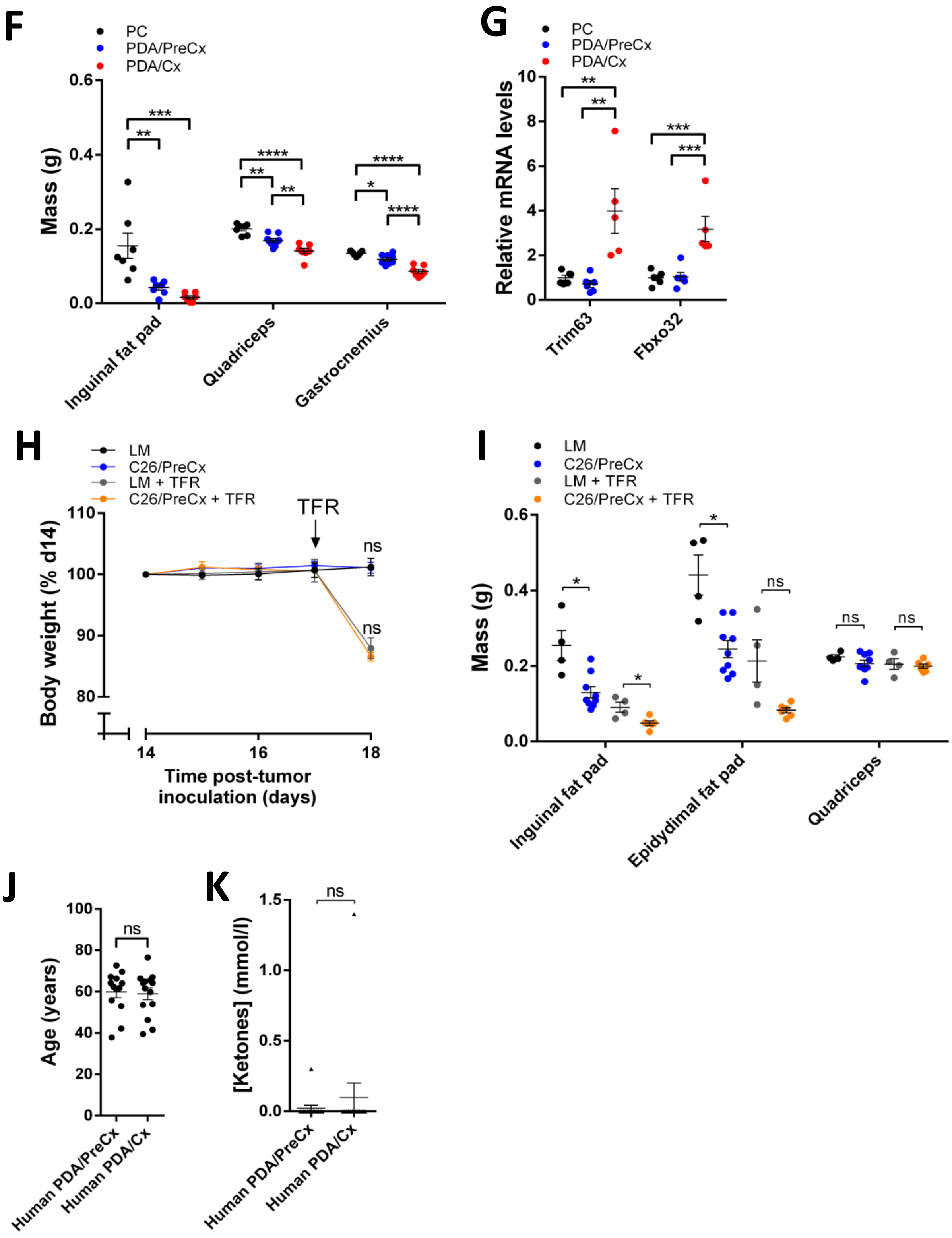

F
PC
PDA/PreCx
PDA/Cx
Mass (g)
0.6
0.4
0.2
0.0
Inguinal fat pad
Quadriceps
Gastrocnemius
G
Relative mRNA levels
Trim63
Fbxo32
H
LM
C26/PreCx
LM + TFR
C26/PreCx + TFR
TFR
ns
Body weight (% d14)
Time post-tumor inoculation (days)
I
Epidydimal fat pad
J
Age (years)
Human PDA/PreCx
Human PDA/Cx
K
[Ketones] (mmol/l)

Figure S2

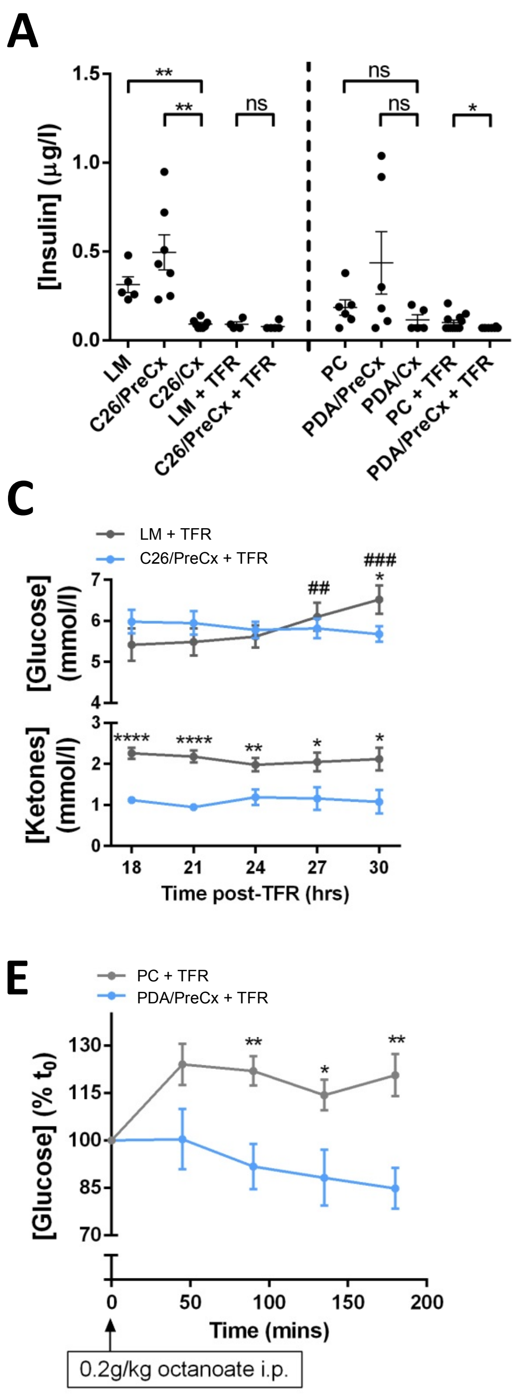
A
[Insulin] (μg/l)
LM
C26/PreCx
C26/Cx
LM + TFR
C26/PreCx + TFR
PC
PDA/PreCx
PDA/Cx
PC + TFR
PDA/PreCx + TFR
B
[Leptin] (pg/l)
C
LM + TFR
C26/PreCx + TFR
[Glucose] (mmol/l)
[Ketones] (mmol/l)
Time post-TFR (hrs)
D
C26/PreCx + TFR
LM + TFR
[Corticosterone] (ng/ml)
Time post-TFR (hrs)
E
PC + TFR
PDA/PreCx + TFR
[Glucose] (% t0)
Time (mins)
0.2g/kg octanoate i.p.

Figure S3

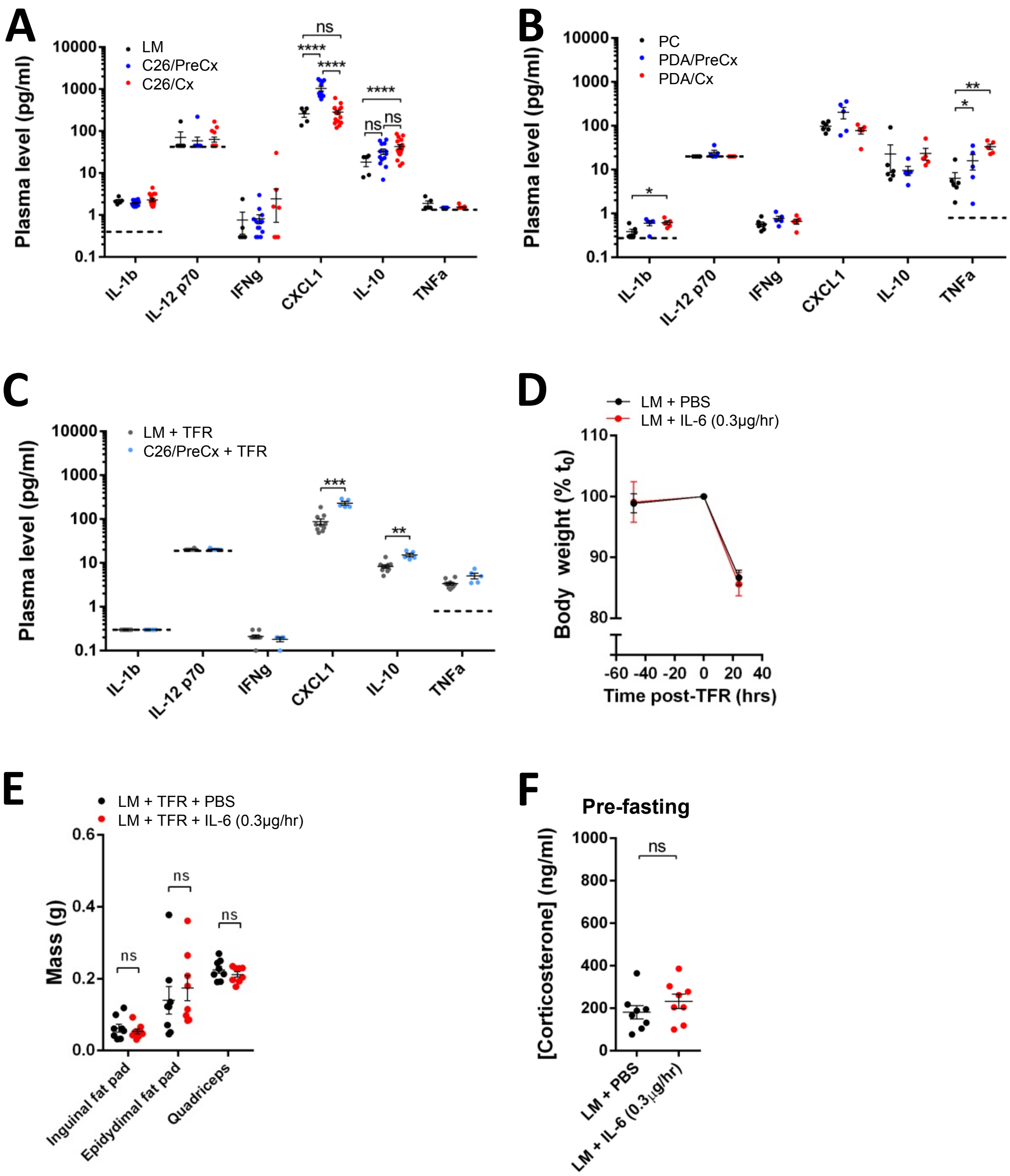

A
LM
C26/PreCx
C26/Cx
Plasma level (pg/ml)
IL-1b
IL-12 p70
IFNg
CXCL1
IL-10
TNFa
B
PC
PDA/PreCx
PDA/Cx
C
LM + TFR
C26/PreCx + TFR
D
LM + PBS
LM + IL-6 (0.3μg/hr)
Body weight (% t0)
Time post-TFR (hrs)
E
LM + TFR + PBS
LM + TFR + IL-6 (0.3μg/hr)
Mass (g)
Inguinal fat pad
Epidydimal fat pad
Quadriceps
F
Pre-fasting
[Corticosterone] (ng/ml)
LM + PBS
LM + IL-6 (0.3μg/hr)

Figure S4

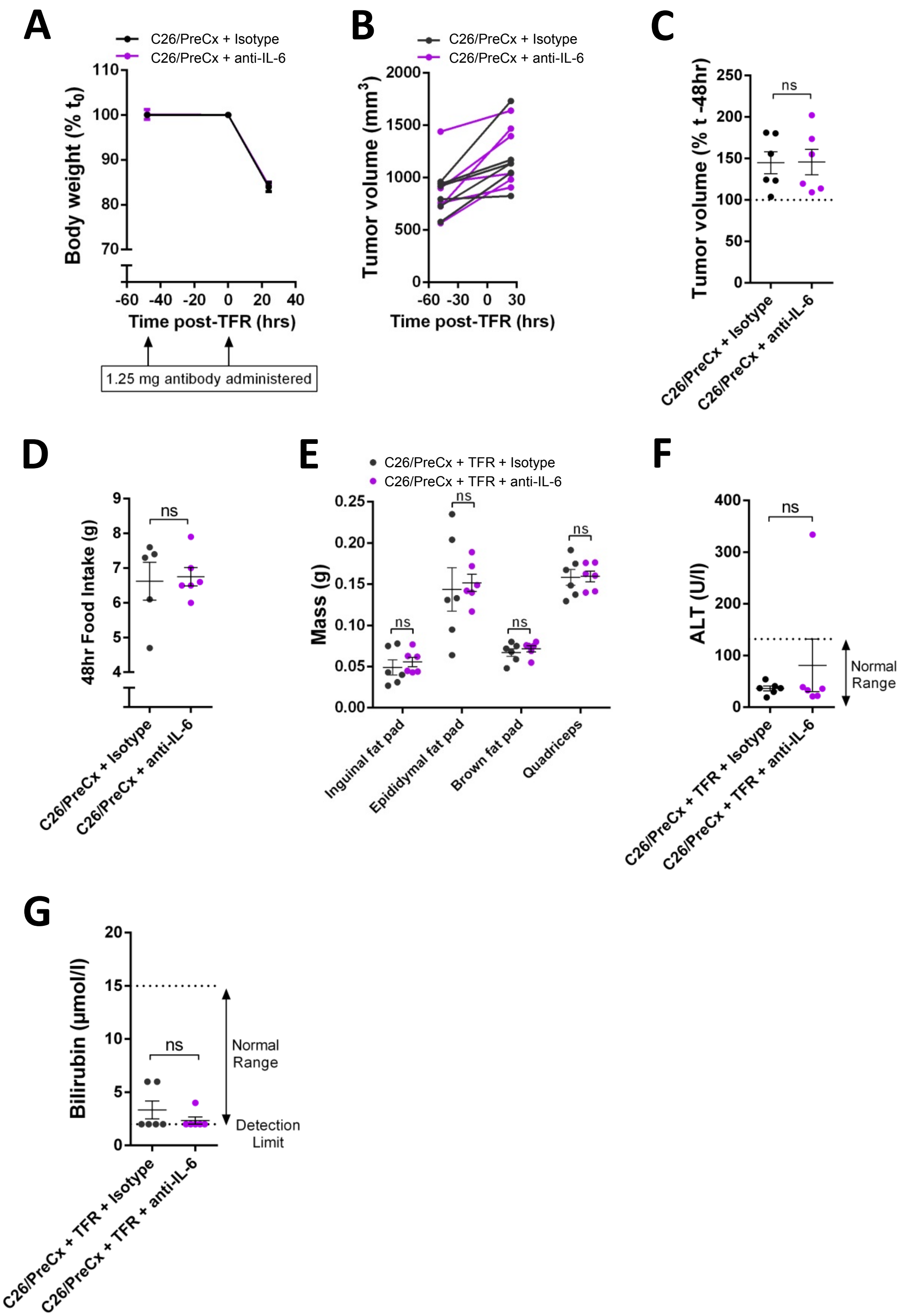
A
C26/PreCx + Isotype
C26/PreCx + anti-IL-6
Body weight (% t_0)
Time post-TFR (hrs)
1.25 mg antibody administered
B
Tumor volume (mm^3)
Time post-TFR (hrs)
C
Tumor volume (% t -48hr)
ns
D
48hr Food Intake (g)
E
C26/PreCx + TFR + Isotype
C26/PreCx + TFR + anti-IL-6
Mass (g)
Inguinal fat pad
Epididymal fat pad
Brown fat pad
Quadriceps
F
ALT (U/l)
Normal Range
G
Bilirubin (μmol/l)
Normal Range
Detection Limit

Figure S5

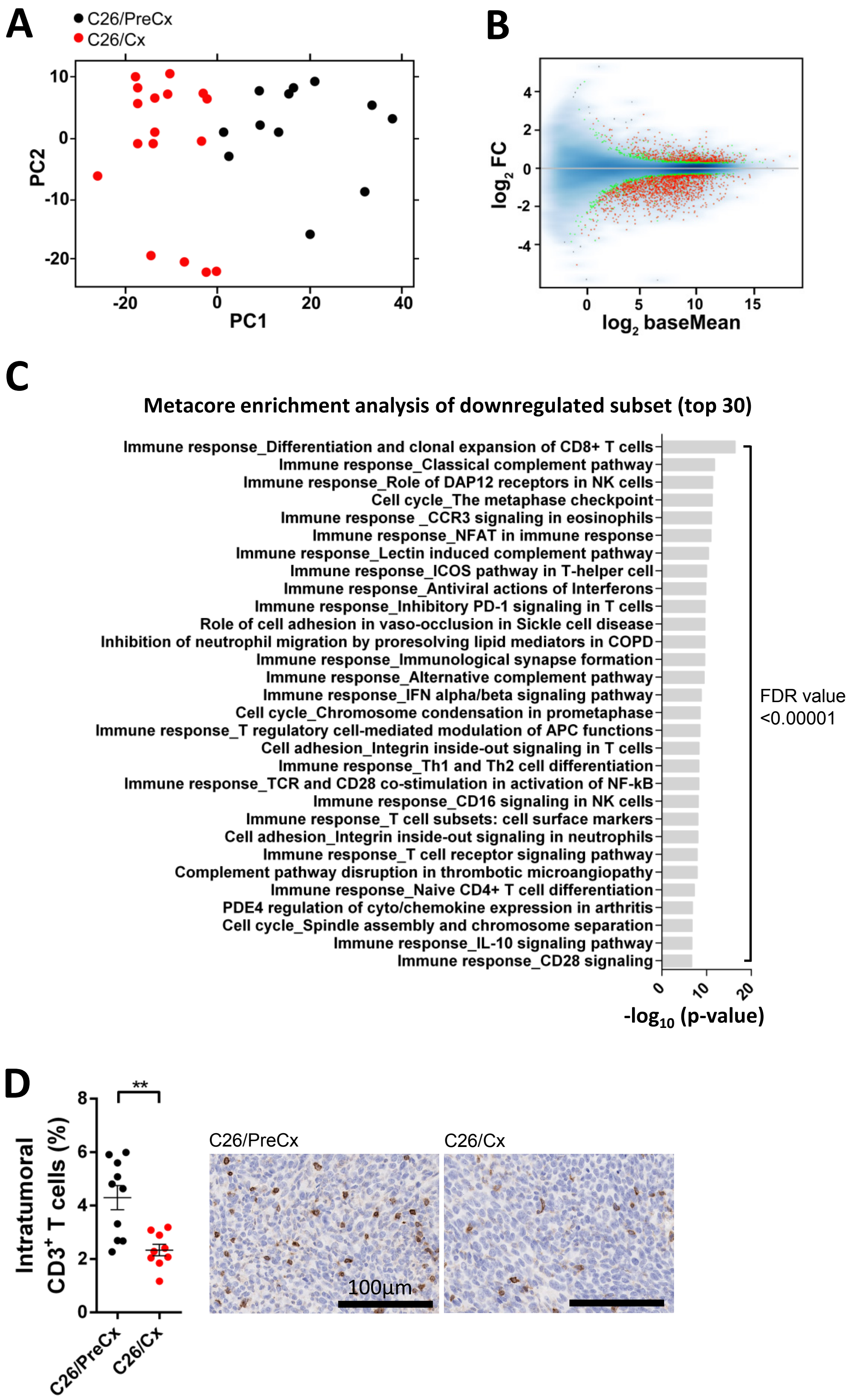
A
C26/PreCx
C26/Cx
PC1
PC2
B
log2 FC
log2 baseMean
C
Metacore enrichment analysis of downregulated subset (top 30)
Immune response_Differentiation and clonal expansion of CD8+ T cells
Immune response_Classical complement pathway
Immune response_Role of DAP12 receptors in NK cells
Cell cycle_The metaphase checkpoint
Immune response _CCR3 signaling in eosinophils
Immune response_NFAT in immune response
Immune response_Lectin induced complement pathway
Immune response_ICOS pathway in T-helper cell
Immune response_Antiviral actions of Interferons
Immune response_Inhibitory PD-1 signaling in T cells
Role of cell adhesion in vaso-occlusion in Sickle cell disease
Inhibition of neutrophil migration by proresolving lipid mediators in COPD
Immune response_Immunological synapse formation
Immune response_Alternative complement pathway
Immune response_IFN alpha/beta signaling pathway
Cell cycle_Chromosome condensation in prometaphase
Immune response_T regulatory cell-mediated modulation of APC functions
Cell adhesion_Integrin inside-out signaling in T cells
Immune response_Th1 and Th2 cell differentiation
Immune response_TCR and CD28 co-stimulation in activation of NF-kB
Immune response_CD16 signaling in NK cells
Immune response_T cell subsets: cell surface markers
Cell adhesion_Integrin inside-out signaling in neutrophils
Immune response_T cell receptor signaling pathway
Complement pathway disruption in thrombotic microangiopathy
Immune response_Naive CD4+ T cell differentiation
PDE4 regulation of cyto/chemokine expression in arthritis
Cell cycle_Spindle assembly and chromosome separation
Immune response_IL-10 signaling pathway
Immune response_CD28 signaling
FDR value
<0.00001
-log10 (p-value)
D
Intratumoral CD3+ T cells (%)
**
C26/PreCx
C26/Cx
100µm

Figure S5

E

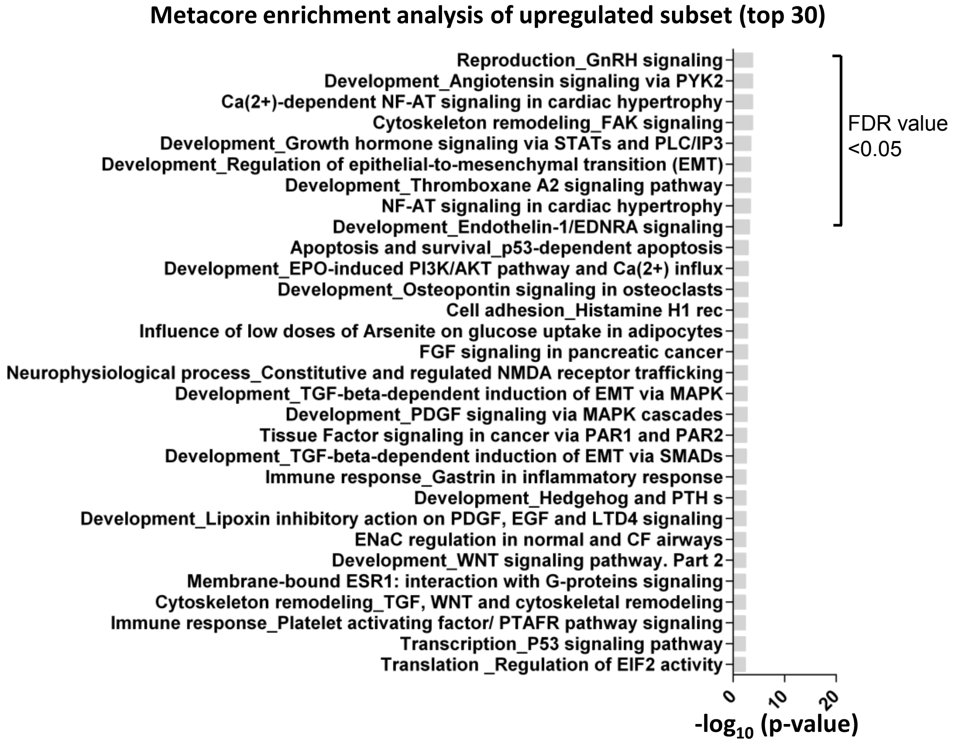
Metacore enrichment analysis of upregulated subset (top 30)
Reproduction_GnRH signaling
Development_Angiotensin signaling via PYK2
Ca(2+)-dependent NF-AT signaling in cardiac hypertrophy
Cytoskeleton remodeling_FAK signaling
Development_Growth hormone signaling via STATs and PLC/IP3
Development_Regulation of epithelial-to-mesenchymal transition (EMT)
Development_Thromboxane A2 signaling pathway
NF-AT signaling in cardiac hypertrophy
Development_Endothelin-1/EDNRA signaling
Apoptosis and survival_p53-dependent apoptosis
Development_EPO-induced PI3K/AKT pathway and Ca(2+) influx
Development_Osteopontin signaling in osteoclasts
Cell adhesion_Histamine H1 rec
Influence of low doses of Arsenite on glucose uptake in adipocytes
FGF signaling in pancreatic cancer
Neurophysiological process_Constitutive and regulated NMDA receptor trafficking
Development_TGF-beta-dependent induction of EMT via MAPK
Development_PDGF signaling via MAPK cascades
Tissue Factor signaling in cancer via PAR1 and PAR2
Development_TGF-beta-dependent induction of EMT via SMADs
Immune response_Gastrin in inflammatory response
Development_Hedgehog and PTH s
Development_Lipoxin inhibitory action on PDGF, EGF and LTD4 signaling
ENaC regulation in normal and CF airways
Development_WNT signaling pathway. Part 2
Membrane-bound ESR1: interaction with G-proteins signaling
Cytoskeleton remodeling_TGF, WNT and cytoskeletal remodeling
Immune response_Platelet activating factor/ PTAFR pathway signaling
Transcription_P53 signaling pathway
Translation _Regulation of EIF2 activity
FDR value
<0.05
0
10
20
-log10 (p-value)

F

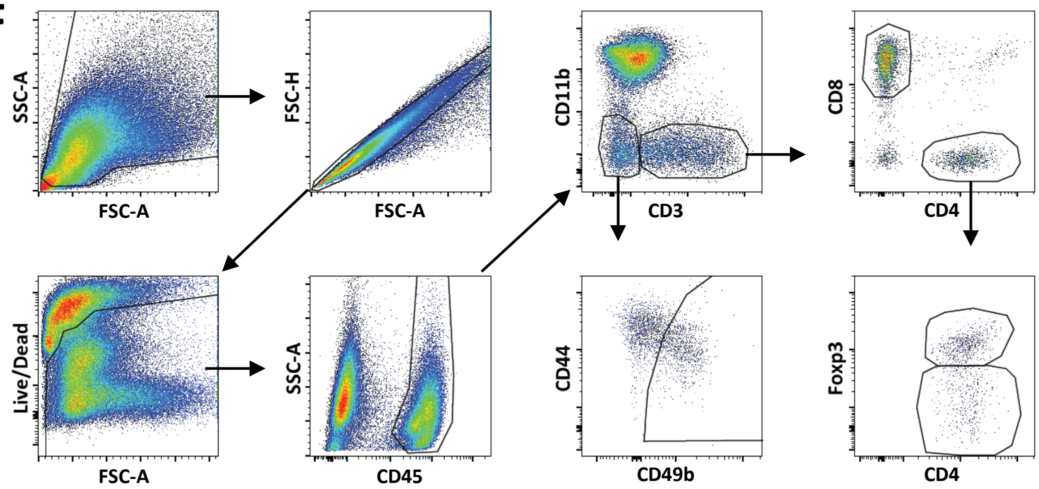
SSC-A
FSC-A
FSC-H
FSC-A
CD11b
CD3
CD8
CD4
Live/Dead
FSC-A
SSC-A
CD45
CD44
CD49b
Foxp3
CD4

Figure S6

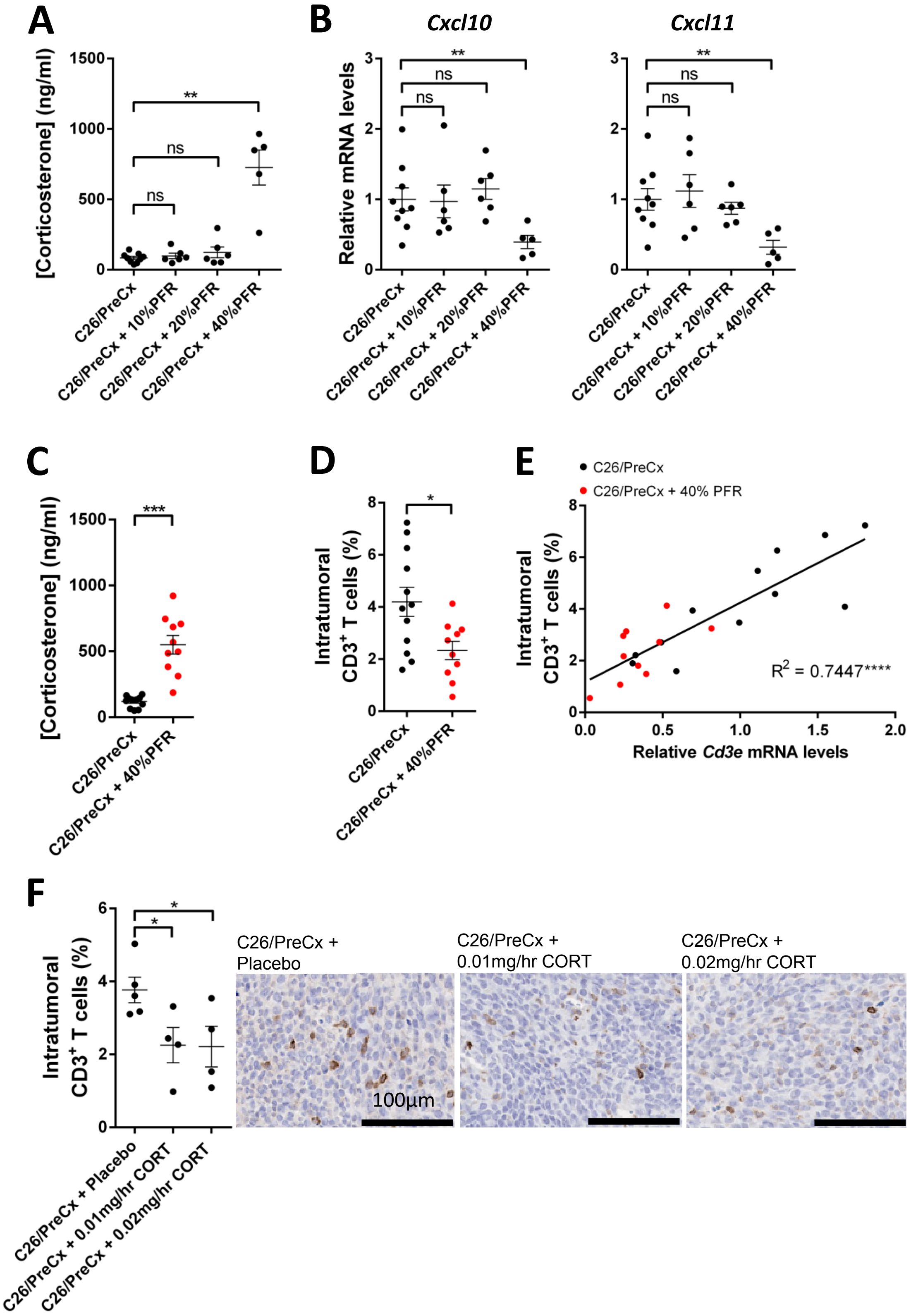

A
[Corticosterone] (ng/ml)
1500
1000
500
0
**
ns
ns
C26/PreCx
C26/PreCx + 10%PFR
C26/PreCx + 20%PFR
C26/PreCx + 40%PFR
B
Cxcl10
Cxcl11
Relative mRNA levels
3
2
1
0
**
ns
ns
C
***
C26/PreCx + 40%PFR
D
Intratumoral CD3+ T cells (%)
8
6
4
2
0
*
E
C26/PreCx
C26/PreCx + 40% PFR
R2 = 0.7447****
Relative Cd3e mRNA levels
0.0
0.5
1.0
1.5
2.0
F
C26/PreCx + Placebo
C26/PreCx + 0.01mg/hr CORT
C26/PreCx + 0.02mg/hr CORT
100µm

Figure S6

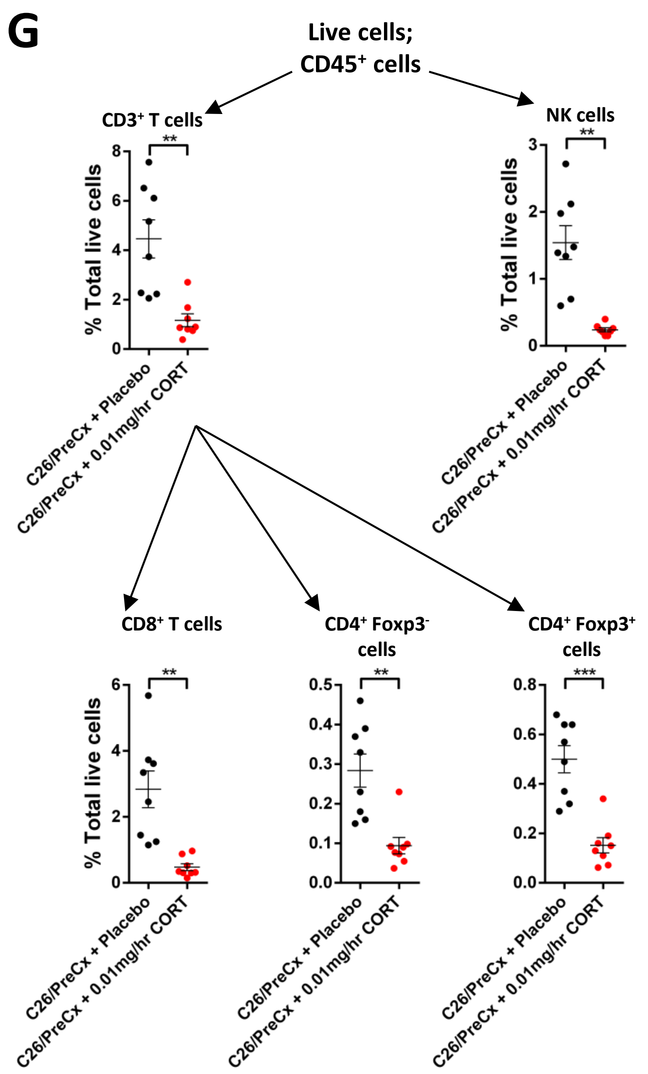

G
Live cells; CD45+ cells
CD3+ T cells
NK cells
CD8+ T cells
CD4+ Foxp3- cells
CD4+ Foxp3+ cells
% Total live cells
**
***
C26/PreCx + Placebo
C26/PreCx + 0.01mg/hr CORT

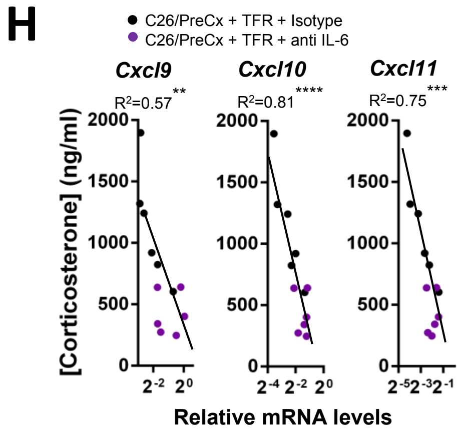

H
C26/PreCx + TFR + Isotype
C26/PreCx + TFR + anti IL-6
Cxcl9
R²=0.57 **
Cxcl10
R²=0.81 ****
Cxcl11
R²=0.75 ***
[Corticosterone] (ng/ml)
Relative mRNA levels

Figure S7

A

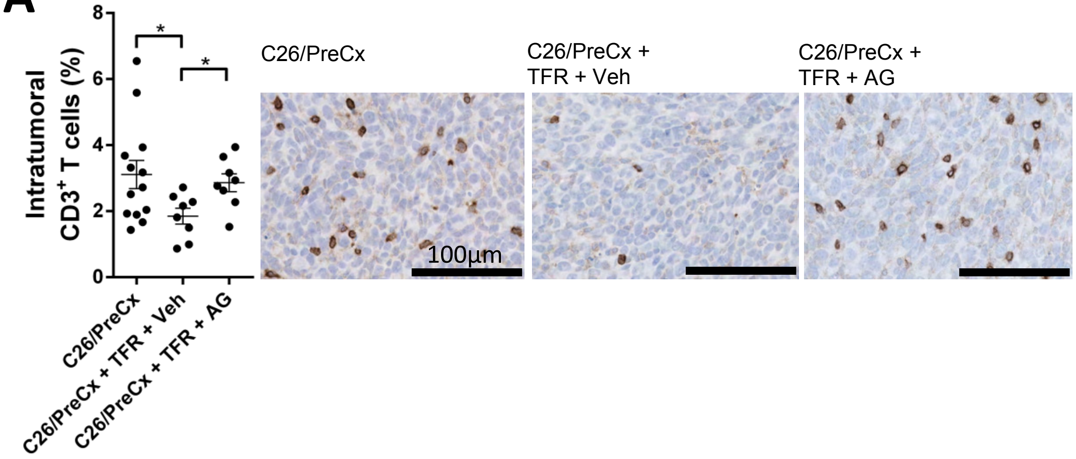

Intratumoral CD3+ T cells (%)
8
6
4
2
0
*
*
C26/PreCx
C26/PreCx + TFR + Veh
C26/PreCx + TFR + AG
C26/PreCx
C26/PreCx + TFR + Veh
C26/PreCx + TFR + AG
100µm

B

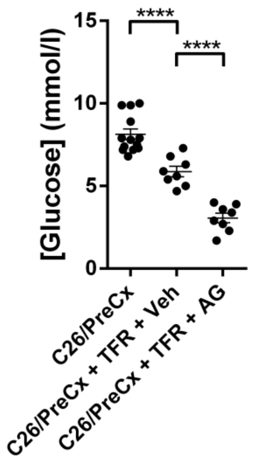

[Glucose] (mmol/l)
15
10
5
0
****
****
C26/PreCx
C26/PreCx + TFR + Veh
C26/PreCx + TFR + AG

Supplemental figure legends

Figure S1, related to Figure 1 and Figure 2: Phenotypic characterization of C26- and PDA-bearing mice, and extended human data. (A) A schematic illustration of pre-cachectic (/PreCx) and cachectic (/Cx) mice from the C26 and PDA model systems, and the respective non-tumor-bearing control groups, is presented. Weight-stable mice with established C26 or PDA tumors that were yet to develop cachexia were termed pre-cachectic (C26/PreCx and PDA/PreCx respectively). C26- and PDA-bearing mice were termed cachectic (C26/Cx and PDA/Cx respectively) following >5% body weight loss from their peak weight. (B) Body weight trajectories from individual C26/PreCx and C26/Cx mice are shown. (C) Representative photograph of masticated food pellets in a cage of a representative individually housed C26/Cx mouse. This phenomenon was not observed in the cages of C26/PreCx mice. The mass of the masticated food was taken into account when calculating the daily food intake for both C26 and PDA model systems. (D) Body composition was assessed via tissue wet weights in LM, C26/PreCx, and C26/Cx mice. (E) mRNA levels of genes involved in muscle catabolism were measured by RT-qPCR in the quadriceps of LM, C26/PreCx, and C26/Cx mice. (F) Body composition and (G) quadriceps mRNA levels were also assessed in PC, PDA/PreCx, and PDA/Cx mice. (H) Changes in body weight and (I) tissue wet weights caused by 24 hr TFR of LM and C26/PreCx mice were measured. Body weights were normalized to d14 post-tumor inoculation, and tissue wet weights were determined at d18. The (J) ages and (K) plasma ketone levels of pre-cachectic and cachectic patients with PDA whose weight changes and serum cortisol levels were presented in Fig. 1F, G are shown. The comparisons in (D-G) were performed using one-way ANOVA with Tukey's correction for post-hoc testing. LM vs C26/PreCx, LM + TFR vs C26/PreCx + TFR, and non-cachectic vs cachectic cancer patient data comparisons in (H-K) were performed using two-tailed t-tests with Welch's correction. *$p<0.05$, **$p<0.01$, ***$p<0.001$, ****$p<0.0001$. Data are presented as mean ±SEM.

Figure S2, related to Figure 1 and Figure 2: Extended characterization of the response to spontaneous and induced food restriction in mice bearing C26 and PDA tumors, respectively. (A) Insulin and (B) leptin plasma levels were measured from terminal blood samples. Dotted lines represent assay detection limits. (C, D) Blood ketone, blood glucose, and plasma corticosterone profiles in C26/PreCx and LM mice were determined 18-30 hrs post-initiation of TFR via tail vein bleeds (n=10 per group). (E) Sodium octanoate was administered to PC and PDA/PreCx mice 24 hrs post-initiation of TFR, and blood glucose levels were measured (n=7-8 per group). Data from LM, C26/PreCx, and C26/Cx mice, and PC, PDA/PreCx, and PDA/Cx mice, were compared using one-way ANOVA with Tukey's correction for post-hoc comparisons. The LM + TFR vs C26/PreCx + TFR, and PC + TFR vs PDA/PreCx + TFR data comparisons in (A, B) as well as data comparisons at each timepoint in (C-E) were performed using two-tailed t-tests with Welch's correction. Additional within-group comparisons of the data from the timecourses in (C, D) were performed using two-tailed ratio paired t-tests of both groups at each timepoint relative to their 18hrs post-TFR measurements (#).* $p<0.05$; ** $p<0.01$; *** $p<0.001$; ****$p<0.0001$. Data are presented as mean +/- SEM.

Figure S3, related to Figure 3: Extended data from plasma cytokine and chemokine screens, and effects of IL-6 infusion on additional metabolic parameters. (A-C) Multi-cytokine array analysis was performed using terminal bleed samples from LM, C26/PreCx , C26/Cx, LM + TFR and C26/PreCx + TFR mice, as well as PC, PDA/PreCx and PDA/Cx mice. Dotted lines represent assay detection limits for the respective target cytokines. (D) Body weight kinetics (n=8 per group) and (E) tissue wet weights at dissection were determined in non-tumor-bearing LM mice after 72 hrs of 0.3μg/hr IL-6 infusion, with TFR during the final 24 hrs. (F) Tail bleed corticosterone levels after 48 hrs of 0.3μg/hr IL-6 infusion and before TFR were determined in LM mice. Data from (A) LM, C26/PreCx, and C26/Cx mice, and (B) PC, PDA/PreCx, and PDA/Cx mice were compared using one-way ANOVA with Tukey's correction for post-hoc comparisons. All other data comparisons as well as comparisons of data at each timepoint in (D) were performed using two-tailed t-tests with Welch's correction. * p<0.05; **p<0.01; ***p<0.001; ****p<0.001. Data are presented as mean +/- SEM.

Figure S4, related to Figure 4: Additional effects of neutralization of IL-6 in C26/PreCx + TFR mice. Mice receiving anti-IL-6 or isotype control antibody were assessed for (A) changes in body weight (n=6 per group), and (B, C) tumor volume. (D) The food intake during the 48 hrs of antibody exposure prior to TFR was measured. Following TFR, which occurred during the final 24 hrs of the experiment, (E) tissue wet weights and terminal bleed plasma levels of (F) ALT and (G) bilirubin were assessed. C26/PreCx + TFR + isotype vs C26/PreCx + TFR + anti-IL-6 data comparisons, including data comparisons at each timepoint in (A), were performed via two-tailed t-tests with Welch's correction. Data are presented as mean ±SEM.

Figure S5, related to Figure 5: Extended RNA-seq and flow cytometry data from tumors taken from C26/PreCx and C26/Cx mice. (A) Principal Components Analysis of RNA-seq data from tumor lysates from C26/PreCx and C26/Cx mice. The first component (PC1) accounted for 45.0% of the variance and separated the two groups. (B) A plot of differential gene expression in tumors from C26/Cx relative to C26/PreCx mice is displayed. The intensity of the blue area represents density, green dots represent changes that were significant at a false discovery rate (FDR) <0.05, red dots represent changes significant at FDR<0.01, and $\log_2$FC is $\log_2$(fold change of C26/Cx transcripts relative to C26/PreCx). (C, E) The significantly down- and up-regulated gene subsets in tumors from C26/Cx relative to C26/PreCx mice, totalling 66.2% and 33.8% of the differentially expressed gene population respectively, were subjected to MetaCore enrichment analysis. The pathways were ranked by $-\log_{10}$ (p-value) and the top 30 pathways for each subset are displayed. (D) $CD3^+$ T cell percentages from C26/PreCx and C26/Cx mice were quantified via IHC. (F) Tumors from an independent cohort of C26/PreCx and C26/Cx mice were subjected to flow cytometric analysis using markers for lymphoid cells. Data comparisons in (D) were performed using two-tailed t-tests with Welch's correction. **p<0.01. Data are presented as mean ±SEM.

Figure S6, related to Figure 6: Effects of partial food restriction, corticosterone infusion, and anti-IL-6 treatment on intratumoral immunity. (A, B) C26/PreCx mice were subjected to 3 days of partial food restriction (PFR) by 10, 20, and 40% relative to baseline. (A) Corticosterone levels were determined from tail bleed samples, and (B) mRNA levels of both *Cxcl10* and *Cxcl11* were determined by RT-qPCR from C26 tumor lysates. In freely feeding C26/PreCx mice and C26/PreCx mice that were subjected to 3 days of 40% PFR, (C) plasma corticosterone levels via tail bleeds, (D) tumoral $CD3^+$ T cell percentages via IHC of whole-tumor sections, and (E) the correlation between $CD3^+$ T cell percentages (as assessed by IHC) and relative *Cd3e* mRNA levels (as assessed by RT-qPCR) were determined (n=10-12 per group). (F) C26-bearing mice were subjected to 0.01mg/hr or 0.02mg/hr corticosterone infusions or placebo control, and $CD3^+$ T cell percentages were determined via IHC. (G) Additional C26-bearing mice were subjected to 0.01mg/hr corticosterone infusion or placebo control, and lymphoid populations were quantified using flow cytometry. (H) C26/PreCx mice were administered isotype control or neutralizing anti-IL-6 antibodies 48 hrs prior to TFR and when TFR was initiated. The tumoral mRNA levels for the CXCR3-related chemokines were measured by RT-qPCR, logarithmically transformed, and plotted versus the terminal bleed plasma corticosterone levels. Data in (E, H) were analysed by linear regression. Data in (F) were compared using one way ANOVA with Fisher's LSD test for post-hoc comparisons. All other indicated data comparisons were performed using two-tailed t-tests with Welch's correction. * $p<0.05$; ** $p<0.01$; *** $p<0.001$; **** $p<0.0001$. Data are presented as mean +/- SEM.

Figure S7, related to Figure 6: Extended data from aminoglutethimide-treated C26/PreCx + TFR mice. C26/PreCx mice were administered aminoglutethimide or vehicle control at 0hr, 8hr, and 16hrs post-TFR and sacrificed 24hrs post-TFR. (A) Intratumoral $CD3^+$ T cell percentages were determined via IHC of whole-tumor sections, and (B) blood glucose levels were measured via tail bleed at 24hrs post-TFR. All indicated comparisons were performed using two-tailed t-tests with Welch's correction. * $p<0.05$; ** $p<0.01$; *** $p<0.001$; **** $p<0.0001$. Data are presented as mean ±SEM. Veh = vehicle; AG = aminoglutethimide.

Table S1, related to Figure 5: Selected differentially expressed genes in tumors from pre-cachectic and cachectic C26 bearing mice.

	(Protein)	Gene	Mean (C26)	Mean (C26/Cx)	Fold Change	p-value (adj.)
**Epithelial Cells**		*Cdh1*	31.24	62.64	2.005	8.49E-03
		Krt20	8475.04	14107.02	1.665	8.86E-05
**Fibroblasts**		*Fap*	677.62	828.98	1.223	1.81E-01
	(aSMA)	*Acta2*	113.93	48.48	0.426	8.14E-06
		Col1a1	16606.91	11264.02	0.678	5.31E-03
		Col1a2	28152.49	24170.74	0.859	1.35E-01
		Col4a1	8504.55	12754.90	1.500	4.87E-05
		Col4a2	5960.03	9566.37	1.605	1.22E-05
**Leucocytes**	(CD45)	*Ptprc*	3161.28	1213.74	0.384	4.11E-09
**Lymphoid Cells**		*Cd3e*	57.09	17.53	0.307	1.08E-03
		Cd4	69.13	26.99	0.390	1.37E-04
		Cd8a	117.38	36.19	0.308	2.73E-04
		Cd8b	36.81	10.09	0.274	1.03E-03
		Klrb1b	178.73	74.75	0.418	4.79E-12
		Klrb1c	48.17	18.99	0.394	1.97E-07
		Gzma	144.82	24.97	0.172	5.29E-04
		Gzmb	258.12	77.72	0.301	9.14E-04
		Prf1	156.53	65.85	0.421	2.62E-05
		Foxp3	14.56	9.85	0.677	6.02E-02
	(CD25)	*Il2ra*	30.96	21.97	0.710	6.62E-02
		Ifng	12.54	2.02	0.161	3.84E-03
	(T-bet)	*Tbx21*	13.59	4.38	0.322	7.89E-05
		Eomes	37.58	23.67	0.630	6.61E-05
	(PD-1)	*Pdcd1*	50.99	20.08	0.394	2.20E-04
**Myeloid Cells**	(CD11b)	*Itgam*	2901.61	1697.48	0.585	1.32E-09
	(CD11c)	*Itgax*	2135.56	1223.86	0.573	1.45E-12
	(MHC II)	*Cd74*	15713.18	5582.11	0.355	2.67E-08
		Cd40	70.24	20.57	0.293	2.26E-08
		Cd68	1212.43	679.46	0.560	<1.21E-13
	(F4/80)	*Emr1*	1427.34	545.88	0.382	8.32E-12
		Ly6c1	328.85	139.20	0.423	3.09E-04
		Ly6c2	105.67	15.81	0.150	5.35E-04
		Ly6g	0.11	0.47	4.112	4.43E-01
		Ccr2	825.30	293.64	0.356	2.61E-09
**Endothelial Cells**	(CD31)	*Pecam1*	964.03	554.18	0.575	6.63E-08
		Vwf	78.15	104.92	1.342	1.09E-01
	(eNOS)	*Nos3*	38.47	34.22	0.889	6.86E-01
**Chemokines**		*Cxcl9*	1384.42	298.51	0.216	<1.21E-13
		Cxcl10	473.52	112.15	0.237	6.27E-07
		Cxcl11	68.17	12.57	0.184	2.91E-06
		Cxcl12	187.27	81.07	0.433	1.56E-03
		Ccl2	1676.61	757.83	0.452	1.91E-03
		Ccl5	133.98	37.68	0.281	4.64E-06
**Growth Factors**	(M-CSF)	*Csf1*	2034.25	1264.97	0.622	8.46E-07
	(GM-CSF)	*Csf2*	1.13	1.03	0.913	9.98E-01
	(G-CSF)	*Csf3*	10.37	33.14	3.196	3.49E-01

Experimental procedures

Animal Experiments

All experiments were performed in accordance with national and institutional guidelines and were approved by the UK Home Office, the animal ethics committee of Cancer Research UK Cambridge Institute, and the University of Cambridge. The C26 model experiments were performed on wild-type male Balb/c mice purchased from Charles River Laboratories and kept on a 24hr 12:12 light-dark cycle. For tumor experiments, cells from the C26 cell line were cultured in RPMI 1640 Medium (+L-Glutamine) with 10% heat-inactivated FBS under sterile conditions. This was followed by non-enzymatic dissociation (Gibco), resuspension in FBS-free RPMI, counting of viable cell concentration using a Vi-Cell counter and injection of $2*10^6$ viable cells subcutaneously into the flank of each mouse, in an RPMI vehicle at 100µl per mouse, at between 8-10 weeks of age. All mice were singly housed on blotting paper bedding post-inoculation so as to enable quantification of food spillover as part of food intake measurements (See Fig. S1C).

C26-bearing mice were termed pre-cachectic from 18 days post-inoculation onwards. Cachexia, defined as >5% loss of body weight from peak body weight, occurred in these mice at 22-30 days post-inoculation. The 24hr fasted pre-cachectic C26-bearing mice (C26/PreCx + TFR) were enrolled in experiments at day 18 post-injection, contingent on prior weight stability, and fasted at 1200h which was the middle of the 12hr daily light period. Only pre-cachectic C26 and littermate control (LM) mice weighing 23-28g at the point of food restriction were enrolled in food restriction experiments. All tumors from experimentally enrolled C26/PreCx + TFR mice weighed 0.7-1.5g upon dissection, were fully located in the flank, and had no macroscopic evidence of ulcerations. C26-bearing mice in all statistical comparisons were matched regarding age and tumor incubation time, and C26-bearers undergoing therapeutic reversal with anti-IL-6 or aminoglutethimide were, in addition, stratified by body weight prior to randomisation to either treatment or control study arms. Anti-IL-6 (MP5.20F3) and isotype IgG1 (HRPN) was purchased from BioXcell, diluted in PBS and injected i.p. at 1.25mg per mouse 48hrs prior to and again at the point of TFR. For the octanoate challenge, sodium octanoate (Sigma) was dissolved to 200mM in 0.9% NaCl and injected i.p. at 6ml/kg in 24hr fasted mice. For aminoglutethimide experiments, aminoglutethimide was dissolved in a PBS vehicle containing 9% DMSO, 1% Tween, and 30% PEG-400 by volume. Aminoglutethimide was administered to pre-cachectic C26-bearing mice at 37.5mg/kg at 0hr, 8hr, and 16hr post-food restriction, with sacrifice 24hrs post-food restriction. Mild sedation and ataxia were observed in some treated mice. For partial food restriction (PFR) experiments, C26-bearing mice were singly housed day 10 post-inoculation and a 7-day baseline food intake was determined for each mouse following a 24hr acclimatization period. Mice were then stratified primarily by baseline food intake and secondarily by body weight, food restricted by 10, 20 or 40% from their individual baselines and fed at 1700h on days 17, 18, and 19 before being sacrificed at 1300h on day 20. For the continuous corticosterone infusion experiments, C26-bearing mice were anaesthetized with isoflurane and subcutaneously implanted with one placebo or one or two 5mg corticosterone pellets (Innovative Research of America) designed to release their content over 21 days. Pellet implantation was initiated at day 13 post-tumor inoculation.

For the IL-6 infusion in non-tumor bearing littermate mice, recombinant carrier-free murine IL-6 was purchased from BioLegend, diluted in PBS, loaded into osmotic minipumps (ALZET model 1003D, which elutes at 1.0µl/hr) at 0.3, 0.1, and 0.03mg/ml, and inserted into the subcutaneous space of singly housed 12 week old male Balb/c mice. The pumps were left to elute for 72hrs with the final 24hrs under TFR. The PBS-infused male Balb/c control group was matched for age and mice were stratified by body weight prior to enrolment into the experimental groups. TFR was initiated in the middle of the 12hr light period at 1200h, and mice were sacrificed after 24hrs of TFR. At this point, some mice were approaching the clinical severity limit.

KPC mice and PC littermates were of various age and male and female mice were enrolled. All experimental groups were randomised for sex and age. Pancreatic ductal adenocarcinoma (PDA) tumors in KPC mice were detected via palpation and high-resolution ultrasound scans (Vevo 2100, VisualSonics), and confirmed at necropsy. All tumours

from treatment trials were confirmed histopathologically as adenocarcinomata. Only weight-stable, PDA-bearing mice with tumors with maximum diameters >4mm (measured by ultrasound) were enrolled in experiments, and these mice were all termed pre-cachectic at the point of enrolment. Pre-cachectic PDA-bearing mice were monitored for body weight and food intake on a daily basis, and were termed cachectic following weight loss of more than 5% from their peak recorded weight. Excluding those PDA-bearing mice that succumbed to ascites, 85% of the PDA-bearing mice that were enrolled as pre-cachectic subsequently developed cachexia over a period of 30 days post enrolment. KPC mice that were found to have macroscopic liver metastases, severe ascites, diarrhoea or bowel obstruction at or before necropsy were excluded from the analyses.

To determine the effects of corticosterone pellets +/- immune therapy on PDA tumors mice, pre-cachectic PDA-bearing KPC mice with average maximum tumor diameters between 4-7mm (measured via ultrasound) and good clinical condition were enrolled at d-2 for treatment studies. The treatment was started at d0 and tumour size was measured using ultrasound scans on d3 and d6. Where possible, tumors were assessed at multiple angles and the average volumes were determined for each timepoint. Subcutaneous insertion of corticosterone or placebo pellets and/or osmotic pumps (ALZET model 1007D containing a 0.22µm filtered 90mg/ml solution of AMD3100) in KPC mice was performed on d0, with anti-PD-L1 (10F.9G2; BioXcell) or isotype (LTF-2; BioXcell) therapy at 0.2mg/mouse on d0, 2 and 4 post-surgery via i.p. injection.

Euthanasia was performed within the 12hr light period between 1200-1400h unless otherwise stated. Terminal bleeds were obtained through exanguination via cardiac puncture under isofluorane anaesthesia and death was confirmed by cervical dislocation. Heparin was used as anti-coagulant for plasma collection. Tail vein bleeds were performed using venesection before anaesthesia and without restraint and blood was collected into heparinized BRAND micro haematocrit capillary tubes (Sigma). Samples were kept on ice at all time. Plasma was prepared by centrifugation at 14,000g for 5 minutes at 4^0C and snap frozen in liquid nitrogen. Organs and other tissues were rapidly dissected in a consistent order within 3 minutes, snap frozen in liquid nitrogen and stored at -80^0C. Aliquots of tumor samples were fixed in 10% neutral buffered formaldehyde for 24hrs at room temperature before being transferred to 70% ethanol and processed for immunohistochemistry.

Murine Blood & Plasma Measurements

Tail bleeds (whole blood) acquired via tail venesection using a scalpel were analysed for glucose and ketone concentrations using Aviva Accu-Check glucometers (Roche) and Freestyle Optium Neo ketometers (Abbott laboratories) respectively. Mice were not restrained for tail bleeds. Corticosterone from heparinized plasma prepared from tail bleeds (collected using heparinized BRAND micro haematocrit capillary tubes) or terminal cardiac bleeds (acquired using a syringe flushed with heparin solution in PBS) where indicated was quantified using either the IBL or the IDS ELISA. The assay provider was switched from IDS to IBL due to IDS discontinuing their ELISA as data for this manuscript were being collected. The sample incubation step from the IBL assay protocol was 3hrs at room temperature so as to reach displacement equilibrium as determined by preliminary data (not shown). The IBL assay was used for the measurements of corticosterone in the following figure panels: 3F; 6B, D; 7C; S2D; S3F; S6C. All other corticosterone assays in this manuscript were performed using the IDS assay. Terminal cardiac bleed plasma glucose levels were assessed using an automated assay on the Siemens Dimension RxL analyser, and terminal bleed plasma ketone levels were assessed using the Stanbio Beta Hydroxybutyrate Liquicolour kit. Insulin, leptin and cytokines for the multi-cytokine array were measured using Meso Scale Discovery kits. The IL-6 levels for Fig. 3C were measured using R&D's Quantikine ELISA for murine IL-6.

AMD3100 was extracted from plasma samples by protein precipitation with methanol of samples pre spiked with internal standard (D_4 labelled AMD3100) and EDTA. Quantitation was by LC-MS/MS over the calibration range 50-5000 ng/mL. LC separation was achieved on a Phenomenex Kinetex 2.6µm Bipehenyl 100x2.1mm using an optimised gradient with 0.1 % formic acid in methanol and water using an Accela UHPLC autosampler and pump.

Electro spray in the positive ionisation mode monitoring the mass transitions 252-420 for AMD3100 and 254-424 for the deuterium labelled internal standard was performed on a Thermo TSQ Vantage.

RT-qPCR

mRNA was extracted from frozen tissues using TRIzol Reagent (Invitrogen) following the manufacture's protocol. The concentration and purity of aqueous RNA from each preparation was assessed using an ND1000 spectrophotometer. mRNA templates were diluted to 2ng/µl (muscle and liver) or 30ng/µl (tumor) and mRNA was analyzed by quantitative real-time PCR using the TaqMan RNA-to-Ct 1-Step Kit (Life Technologies). The detection threshold for all samples including controls in a given run was manually adjusted so that it lay within the exponential phase of product amplification. mRNA levels were normalized to either *Rn18s* (liver and tumor) or *Tbp* (quadriceps) using the ddCt method. Tumor lysate *Rn18s* values were measured on a 1 in 10 dilution of the template to ensure that the results for this parameter lay within the log-linear range of the standard curve. The following TaqMan gene expression assays were used: Mm01277044_m1 (*Tbp*); Mm03928990_g1 (*Rn18s*); Mm00440939_m1 (*Ppara*); Mm01323360_g1 (*Acadm*); Mm00550050_m1 (*Hmgcs2*); Mm00499523_m1 (*Fbxo32*); Mm01185221_m1 (*Trim63*); Mm00434946_m1 (*Cxcl9*); Mm99999072_m1 (*Cxcl10*); Mm00444662_m1 (C*xcl11*); Mm00599683_m1 (*Cd3e*); Mm01182108_m1 (*Cd8a*); Mm01168134_m1 (*Ifng*); Mm00442834_m1 (*Gzmb*); Mm00812512_m1 (*Prf1*)

RNA-sequencing

RNA extracted from frozen tissues via TRIzol was run through Qiagen RNeasy columns following the "RNA cleanup" protocol. Integrity was confirmed using RIN values (Agilent) with a cutoff of 8, and libraries were prepared using the Illumina TruSeq mRNA Stranded Sample prep kit (96 index High Throughput) using 12 rounds of PCR. Libraries were quantity checked and normalized using Kapa Biosystem's Library Quantification Kit. Library Quality was assessed using Agilent Bioanalyser DNA high sensitivity kits. The final pooled library was run on a MiSeq to assess final sequencing quality before HiSeq 2500 V4 single end 50bp sequencing. We aimed to generate 10-20M single-end 50bp reads per sample.

For the analysis, single-end 50bp reads were aligned to the mouse genome version GRCm38.74 using TopHat v2.0.4 (Trapnell et al., 2009). Read counts were obtained using HTSeq-count v0.5.3p9 (http://www-huber.embl.de/users/anders/HTSeq/doc/overview.html). Read counts were normalized and tested for differential gene expression using the Bioconductor package DESeq v1.10.1 (Anders and Huber, 2010). Multiple testing correction was applied using the Benjamini-Hochberg method. GSEA was performed by ranking all genes tested in RNA-Seq using $-\log_{10}$ (p-values) derived from differential expression analyses and testing against MSigDB Canonical Pathways (C2:CP). Metacore enrichment analyses gated on the significantly (FDR<0.05) up- and down-regulated genes were also performed using Metacore's pathway maps database.

Immunohistochemistry

Tissues were fixed in 10% neutral buffered formaldehyde for 24hrs at room temperature before transferral to 70% ethanol and embedding into paraffin wax. All sections were cut at 3µm and dried at 60°C for 1 hr. Immunohistochemistry was performed for CD3 using a Leica Bond III immunostainer (Leica, Milton Keynes, UK). Deparaffinisation and rehydration was conducted on a Leica ST5020 before transferring to the Bond III where antigen retrieval was performed using Tris EDTA, pH9 (ER2) for 20 mins at 100 °C, incubation in a rabbit polyclonal anti-CD3 antibody (A0452) for 15 mins, HRP-linked anti-rabbit polymer for 8 mins, diaminobenzidine for 10 mins and DAB Enhancer (Leica) for 10 mins. Counterstaining was with Haematoxylin (Leica) for 2 mins. All incubations were performed at room temperature and all incubation steps were followed with 3 washed in TBS (Bondwash, Leica). Following application of cover slips and drying, the slides were scanned at 20x on a Leica AT2 and subsequently analysed in a blinded manner using the Cytonuclear v1.4 algorithm on the HALO platform (Indica Labs).

Flow Cytometry

C26 tumors were dissected and placed in ice-cold PBS. Portions of each tumor were then minced using razor blades, and placed into a ice-cold digestion buffer containing RPMI with 1mg/ml collagenase (Sigma C0130) and 0.1mg/ml DNAse (Sigma D4527). The suspensions were warmed to 37 ^{0}C and agitated at 750rpm for 60 minutes using an Eppendorf Thermomixer Comfort. The suspensions were triturated for 5 seconds at the 0, 20, 40, and 60 minute timepoints during the agitation period. At 60 minutes, EDTA was added to the suspension to a final concentration of 10mmol/l, and 5 minutes after that the suspension was pipetted up and down before being passed though at 70µm cell strainer. 5 volumes of flow cytometry staining buffer (PBS containing 5% heat-inactivated FBS, 0.05% sodium azide, and 2mmol/l EDTA) were added through the filter at this point and the resulting single-cell suspension was kept on ice. Cells were stained for flow cytometry according to reagent manufacturers' protocols.Viability was determined using eBioscience e780 fixable viability dye at a 1/1000 dilution. The following antibodies were used to identify immune cell subsets: anti-CD45 (BUV395; 30-F11; 0.5µg/ml; BD); anti-CD3e (PE; 145-2C11; 1µg/ml; Biolegend); anti-CD4 (BV605; GK1.5; 1µg/ml; Biolegend); anti-CD8a (FITC; 53-6.7; 2.5µg/ml; Biolegend); anti-CD49b (PE-Cy7; DX5; 2µg/ml; Biolegend); anti-CD11b (BV786; M1/70; 0.5µg/ml; Biolegend); anti-Foxp3 (APC; FJK-16s; 3µg/ml; eBioscience); anti-CD44 (PerCP/Cy5.5; IM7; 0.2µg/ml; Biolegend). CD49b positivity was determined using a fluorescence-minus-one control. Anti-CD16/32 (Unconjugated; 2.4G2; 5 µg/ml; BD) was used for Fc-blocking prior to antibody staining. The eBioscience Foxp3/Transcription Factor Fixation/Permeabilization Concentrate and Diluent was used prior to Foxp3 staining. Sample analysis was performed using an LSR II cytometer. At least 200,000 events per tumor sample were collected. Data were subsequently analysed using FlowJo.

Human studies

The study and the sample acquisition were performed in concordance with local and national guidelines. Patients at least 18 years old with histologically confirmed pancreatic adenocarcinoma were recruited in the outpatient department of the Cambridge University Hospital NHS Foundation Trust as part of the CAMPAN study. Important exclusion criteria were confirmed second malignancies and previous steroid therapy other than anti-emetic therapy with steroids more than 14 days preceding sample acquisition. All participants provided written informed consent. All samples were taken between 1300h and 1500h. For serum acquisition, blood samples were spun at 14000rpm in a table top centrifuge for 5 min after 15min of coagulation. Plasma was obtained using heparin as anti-coagulant with the same centrifugation protocol. Plasma and serum samples were snap frozen in aliquots. Patients in both study cohorts were matched with regard to age, sex, and treatment history. Patients were designated cachectic if they met the international consensus definition for cachexia i.e. if they exhibited weight loss of >5% over past 6 months (in the absence of simple starvation) or BMI <20 and ongoing weight loss of more than 2% (Fearon et al., 2011). Serum cortisol levels were assessed using the Cortisol Parameter Assay Kit from R&D systems. Serum IL-6 levels were assessed using the Human IL-6 Quantikine ELISA from R&D systems. Ketone levels from plasma were assessed using Freestyle Optium Neo ketometers (Abbott laboratories).

Statistical Analysis

Statistical analyses, unless otherwise indicated, were performed using GraphPad Prism 6.

References

Anders, S., and Huber, W. (2010). Differential expression analysis for sequence count data. Genome Biol. *11*, R106.

Fearon, K., Strasser, F., Anker, S.D., Bosaeus, I., Bruera, E., Fainsinger, R.L., Jatoi, A., Loprinzi, C., MacDonald, N., Mantovani, G., et al. (2011). Definition and classification of cancer cachexia: an international consensus. Lancet Oncol. *12*, 489–495.

Trapnell, C., Pachter, L., and Salzberg, S.L. (2009). TopHat: discovering splice junctions with RNA-Seq. Bioinformatics *25*, 1105–1111.
