# Supplementary material for: Tumor-Induced IL-6 Reprograms Host Metabolism to Suppress Anti-tumor Immunity
Source: Cell Metab. 2016 Nov 8;24(5):672–84. doi: 10.1016/j.cmet.2016.10.010 (PMC5106372; doi:10.1016/j.cmet.2016.10.010)
Supplement: Document S2. Article plus Supplemental Information [file mmc2.pdf]

Cell Metabolism

Article

Tumor-Induced IL-6 Reprograms Host Metabolism to Suppress Anti-tumor Immunity

Graphical Abstract

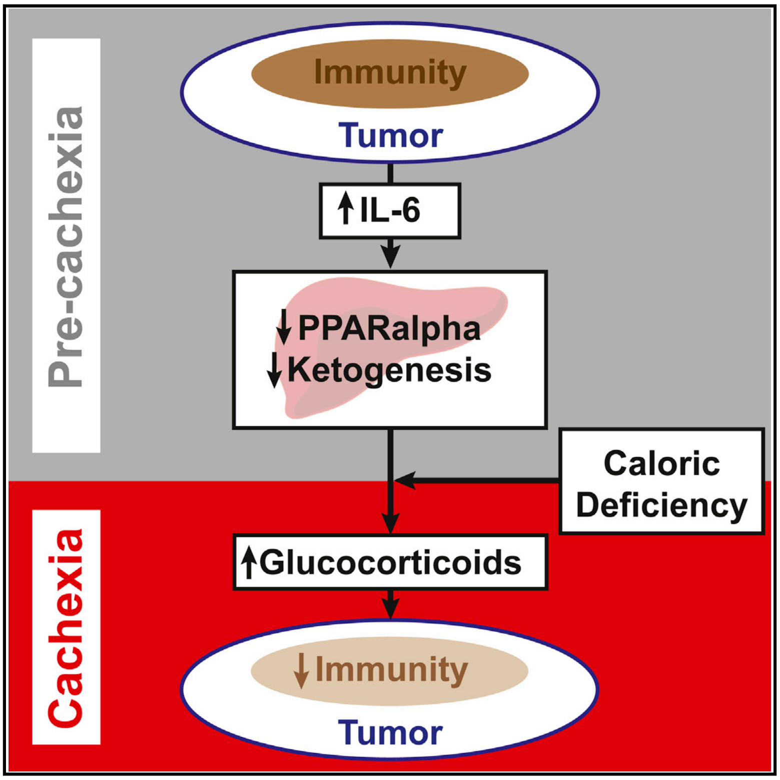

Highlights

- IL-6 suppresses hepatic ketogenesis in pre-cachectic, tumor-bearing mice
- During caloric deficiency, hypoketonemia triggers marked glucocorticoid secretion
- Glucocorticoids, induced by metabolic stress, suppress intratumoral immunity
- Stress-induced glucocorticoids cause failure of cancer immunotherapy

Authors

Thomas R. Flint, Tobias Janowitz, Claire M. Connell, ..., Anthony P. Coll, Duncan I. Jodrell, Douglas T. Fearon

Correspondence

tf261@cam.ac.uk (T.R.F.),
tj212@cam.ac.uk (T.J.)

In Brief

Flint and Janowitz et al. reveal the intricate links between cancer cachexia, hepatic metabolism, and tumor immunology. They find that tumor-induced IL-6 suppresses hepatic ketogenesis, and during caloric deficiency, this triggers marked glucocorticoid secretion. This hormonal stress response suppresses intratumoral immunity and causes failure of anti-cancer immunotherapy.

Flint et al., 2016, Cell Metabolism *24*, 672–684
November 8, 2016 © 2016 The Authors. Published by Elsevier Inc.
http://dx.doi.org/10.1016/j.cmet.2016.10.010

Cell Metabolism
Article

Tumor-Induced IL-6 Reprograms Host Metabolism to Suppress Anti-tumor Immunity

Thomas R. Flint,[1,7,*] Tobias Janowitz,[1,2,7,8,*] Claire M. Connell,[1,2] Edward W. Roberts,[3] Alice E. Denton,[1] Anthony P. Coll,[4] Duncan I. Jodrell,[1] and Douglas T. Fearon[1,5,6]
[1]Cancer Research UK Cambridge Institute, University of Cambridge, Li Ka Shing Centre, Cambridge CB2 0RE, UK
[2]Department of Oncology, University of Cambridge, Addenbrooke's Hospital, Cambridge CB2 0QQ, UK
[3]Department of Pathology, University of California, San Francisco, San Francisco, CA 94143, USA
[4]University of Cambridge Metabolic Research Laboratories, MRC Metabolic Diseases Unit, Level 4, Wellcome Trust-MRC Institute of Metabolic Science, Addenbrooke's Hospital, Cambridge CB2 0QQ, UK
[5]Cold Spring Harbor Laboratory, Cold Spring Harbor, NY 11724, USA
[6]Weill Cornell Medical College, New York, NY 10021, USA
[7]Co-first author
[8]Lead Contact
*Correspondence: tf261@cam.ac.uk (T.R.F.), tj212@cam.ac.uk (T.J.)
http://dx.doi.org/10.1016/j.cmet.2016.10.010

SUMMARY

In patients with cancer, the wasting syndrome, cachexia, is associated with caloric deficiency. Here, we describe tumor-induced alterations of the host metabolic response to caloric deficiency that cause intratumoral immune suppression. In pre-cachectic mice with transplanted colorectal cancer or autochthonous pancreatic ductal adenocarcinoma (PDA), we find that IL-6 reduces the hepatic ketogenic potential through suppression of PPARalpha, the transcriptional master regulator of ketogenesis. When these mice are challenged with caloric deficiency, the resulting relative hypoketonemia triggers a marked rise in glucocorticoid levels. Multiple intratumoral immune pathways are suppressed by this hormonal stress response. Moreover, administering corticosterone to elevate plasma corticosterone to a level that is lower than that occurring in cachectic mice abolishes the response of mouse PDA to an immunotherapy that has advanced to clinical trials. Therefore, tumor-induced IL-6 impairs the ketogenic response to reduced caloric intake, resulting in a systemic metabolic stress response that blocks anti-cancer immunotherapy.

INTRODUCTION

Pancreatic ductal adenocarcinoma (PDA) is a leading cause of cancer death (Siegel et al., 2012). It is poorly responsive to available chemotherapies and unresponsive to checkpoint-targeted immunotherapies (Brahmer et al., 2012; Royal et al., 2010), and it predisposes patients to the lethal wasting syndrome, cachexia (Bachmann et al., 2013). Resistance to chemotherapy and immune evasion are topics of active research in PDA (Feig et al., 2013; Olive et al., 2009), but how PDA tumors alter host metabolism in cachexia and whether this affects the host's immune interaction with the tumor are less studied questions.

Cachexia, which is clinically defined by weight loss, represents a spectrum of disease. It initially arises as pre-cachexia, progresses to cachexia, and then progresses to refractory cachexia, a process that is driven by negative energy balance and abnormal metabolism (Fearon et al., 2011). The pathogenesis and progression of cachexia have been attributed in part to systemic elevations of pro-inflammatory cytokines (Fearon et al., 2012), but anti-cytokine therapies alone have not demonstrated clinical benefit. Cachexia-enhancing alterations such as increased catabolic signaling and increased uncoupling protein expression have also been described at the level of muscle and fat tissues, respectively (Kir et al., 2014; Petruzzelli et al., 2014; Zhou et al., 2010). It is not known, however, whether the normal host response to caloric deficiency, a central component of the syndrome (Fearon et al., 2011), is itself impaired in cachexia. Such an impairment might explain why macronutrient supplementation has failed to reverse cachexia in clinical trials (Tisdale, 2009).

Altered macronutrient utilization must also be considered in the context of tumor biology and therapeutic resistance. Previous studies have highlighted the capacity of cancer cells to respond to alterations of the host metabolism (Kalaany and Sabatini, 2009; Park et al., 2010), but the stromal constituents of the tumor may also sense such alterations. One possibility, considering the sensitivity of intratumoral T cells to their local metabolic environment (Chang et al., 2015; Ho et al., 2015), as well as the marked effects of poor nutritional status on systemic immunity in other contexts (Keusch and Farthing, 1986), is a suppressive effect of cachexia on the T cell-mediated anti-tumor immune reaction. Such an effect could contribute to the failure of patients with PDA to respond to the current generation of T cell checkpoint targeted immunotherapies.

In the present study, we have examined the interactions between cancer, systemic metabolism, and tumor immunology in mice using two cancer types that have been documented to predispose to cachexia, the transplanted C26 model of colorectal cancer (Tanaka et al., 1990) and the genetically engineered, autochthonous LSL-Kras$^{G12D/+}$; LSL-Trp53$^{R172H/+}$;

 © 2016 The Authors. Published by Elsevier Inc.
This is an open access article under the CC BY license (http://creativecommons.org/licenses/by/4.0/).

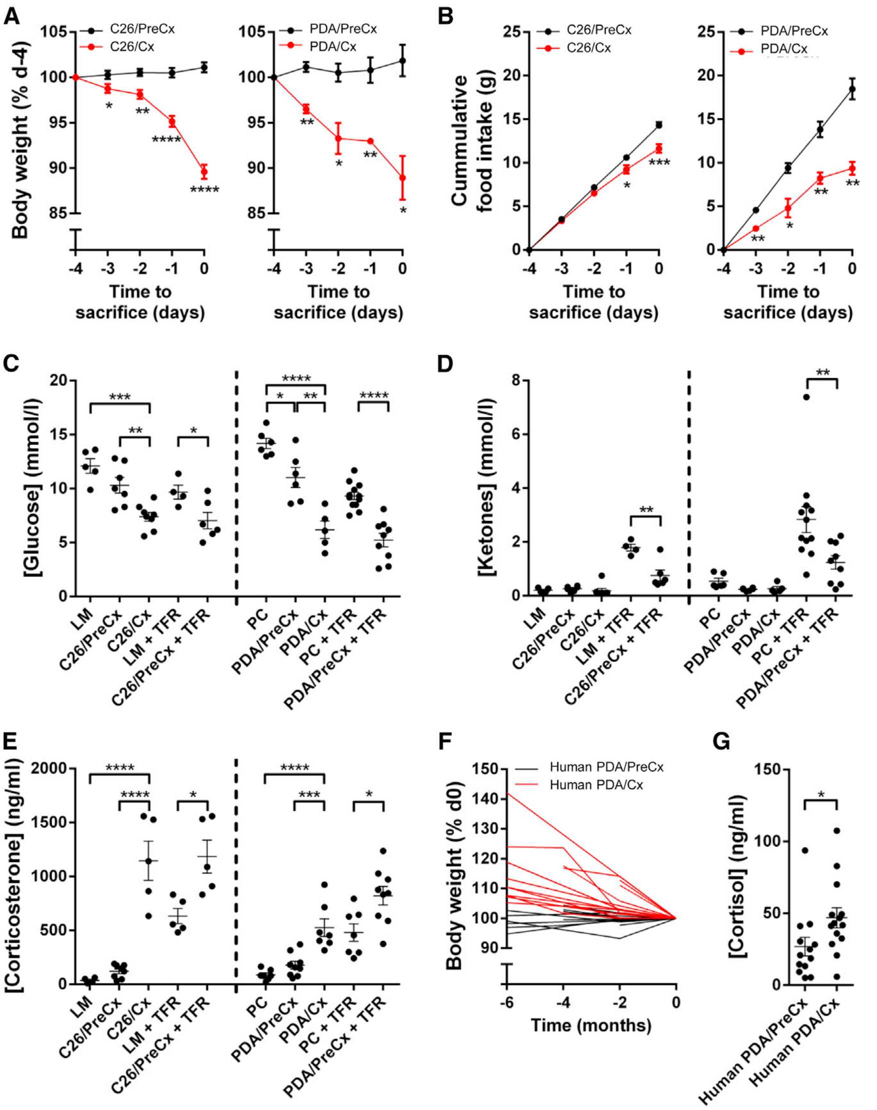

Figure 1. The Metabolic Response to Spontaneous or Imposed Food Restriction of Pre-cachectic Mice Bearing Transplantable C26 Tumors or Autochthonous PDA, and Translational Correlates in Patients with PDA

Weight-stable mice with established C26 or PDA tumors that were yet to develop cachexia were termed pre-cachectic (C26/PreCx and PDA/PreCx, respectively). C26- and PDA-bearing mice were termed cachectic (C26/Cx and PDA/Cx, respectively) following >5% body weight loss from their peak weight.

(A and B) Body weight (A) and cumulative food intake (B) were monitored in C26/PreCx, C26/Cx, PDA/PreCx, and PDA/Cx mice, and measurements were aligned to the time of sacrifice.

(C–E) Terminal bleed plasma levels of (C) glucose and (D) ketones and tail bleed plasma levels of (E) corticosterone were assessed in littermates (LM), C26/PreCx and C26/Cx mice, and LM and C26/PreCx mice that had been subjected to 24 hr TFR (LM + TFR and C26/PreCx + TFR, respectively). Parallel assessments were performed in PDA-bearing mice, with LSL-Trp53$^{R172H/+}$; Pdx-1-Cre/+ (PC) mice as the non-tumor-bearing controls.

(F) Longitudinal pre-sampling weights for pre-cachectic and cachectic patients with pancreatic cancer are displayed.

(G) Midday serum cortisol levels for both groups of patients are shown. The comparisons of data for LM, C26/PreCx, and C26/Cx mice, and for PC, PDA/PreCx, and PDA/Cx mice, were performed using one-way ANOVA with Tukey's correction for post hoc testing. The LM + TFR versus C26/PreCx + TFR, PC + TFR versus PDA/PreCx + TFR, and pre-cachectic versus cachectic patient comparisons were each performed separately using two-tailed t tests with Welch's correction.

*$p < 0.05$, **$p < 0.01$, ***$p < 0.001$, ****$p < 0.0001$. Data are presented as mean ± SEM.

Pdx-1-Cre/+ (KPC) model of PDA (Hingorani et al., 2005; Roberts et al., 2013).

RESULTS

Reprogrammed Hepatic Metabolism Is Evident in Pre-cachectic and Cachectic Mice

C26 and PDA tumors cause cachexia, as defined by loss of body weight (Figures 1A, S1A, and S1B, available online), depletion of fat and muscle mass (Figures S1D and S1F), and elevated markers of muscle catabolism (Figures S1E and S1G). In both model systems, we observed that cachexia was associated with decreased food intake (Figures 1B and S1C). The cachexia-associated reduction in food intake was more pronounced in PDA-bearing relative to C26-bearing mice despite similar rates of weight loss, possibly reflecting differences in tumor size, tumor metabolic activity, and/or murine activity levels. Given the relevance of reduced food intake to cachexia (Fearon et al., 2011), we examined more closely the response to reduced food intake in the C26 and PDA systems. In order to avoid the confounding effects of differing food intake patterns that are well documented as a problem of pair-feeding experiments (Ellacott et al., 2010), we subjected weight-stable, pre-cachectic C26- and PDA-bearing mice, and the respective non-tumor-bearing littermate control mice, to 24 hr total food restriction (TFR) (Figures S1H and S1I). We measured endocrine markers and metabolites in all experimental groups (Figures 1C–1E and S2A–S2D). Cachectic and food-restricted pre-cachectic C26- and PDA-bearing mice had lower plasma glucose and ketone levels relative to food-restricted non-tumor-bearing controls (Figures 1C, 1D, and S2C). Metabolic stress, as measured by plasma levels of corticosterone, occurred in all food-restricted and cachectic mice (Figure 1E), but the highest corticosterone levels were observed in the food-restricted pre-cachectic groups, presumably in response to low levels of ketones and glucose (Figures 1C–1E, S2C, and S2D) (Amiel et al., 1991). Low levels of ketones and raised glucocorticoid levels during periods of continuous weight loss are not restricted to mouse

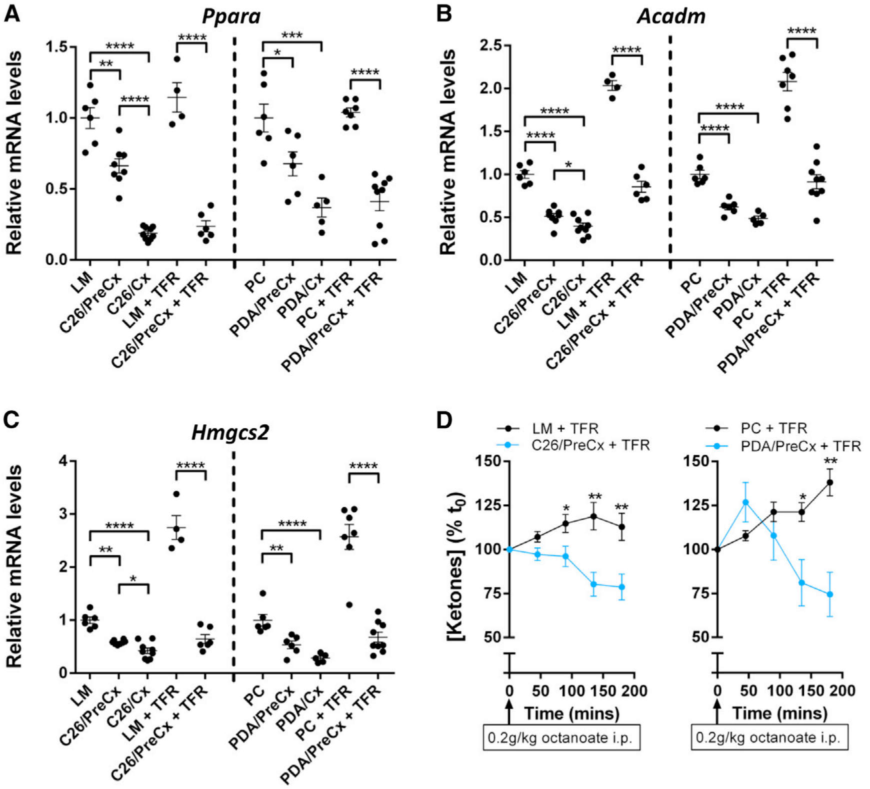

Figure 2. Reprogrammed Hepatic Response to Caloric Deprivation in Pre-cachectic and Cachectic Mice

(A–C) mRNA expression levels of (A) *Ppara*, (B) *Acadm*, and (C) *Hmgcs2* genes involved in mitochondrial beta-oxidation and ketogenesis were measured via qRT-PCR in livers taken from LM, C26/PreCx, C26/Cx, LM + TFR, C26/PreCx + TFR, PC, PDA/PreCx, PDA/Cx, PC + TFR, and PDA/PreCx + TFR mice. All measurements were normalized to the respective freely feeding non-tumor-bearing control groups.

(D) The ketogenic reserve was assessed in LM + TFR, C26/PreCx + TFR, PC + TFR, and PDA/PreCx + TFR mice in vivo by administration of sodium octanoate 24 hr post-TFR. Blood ketone concentrations were measured for up to 180 min post-substrate administration (n = 7–12 per group). The comparisons between LM, C26/PreCx, and C26/Cx mice, and between PC, PDA/PreCx, and PDA/Cx mice, were performed using one-way ANOVA with Tukey's correction for post hoc testing. Comparisons between LM + TFR and C26/PreCx + TFR mice, and between PC + TFR and PDA/PreCx + TFR mice, were performed using two-tailed t tests with Welch's correction.

$^{*}p < 0.05$, $^{**}p < 0.01$, $^{***}p < 0.001$, $^{****}p < 0.0001$. Data are presented as mean ± SEM.

models of cancer, for they were also present in patients with PDA and cachexia (Figures 1F, 1G, S1J, and S1K).

Ketogenesis occurs in the liver and depends on the transcription factor PPARalpha, as demonstrated by the severely impaired ketogenesis in mice with a germline deletion of the *Ppara* gene (Kersten et al., 1999). Hepatic *Ppara* mRNA levels were significantly lower in pre-cachectic C26- and PDA-bearing mice than in non-tumor-bearing control mice. They were further decreased in both cachectic and food-restricted pre-cachectic C26- and PDA-bearing mice, but not in the food-restricted non-tumor-bearing control groups (Figure 2A). Hepatic mRNA levels for *Acadm* and *Hmgsc2*, target genes of PPARalpha (Mandard et al., 2004), were significantly decreased in all tumor-bearing mice that exhibited suppression of *Ppara* mRNA (Figures 2B and 2C). The products of these genes mediate the mitochondrial beta oxidation and conversion to ketones of the free fatty acids that have been released from adipose tissues during caloric deprivation. Their relatively diminished level of expression may therefore explain the low ketone levels that we observed in cachectic mice and food-restricted pre-cachectic mice. Impaired ketogenic potential in food-restricted pre-cachectic C26- and PDA-bearing mice was confirmed by the significantly reduced blood ketone levels following intraperitoneal (i.p.) administration of the ketogenic substrate, octanoate (McGarry and Foster, 1971), as compared to the ketone levels in the food-restricted non-tumor-bearing control groups (Figure 2D). Food-restricted PDA-bearing mice also exhibited reduced blood glucose in response to octanoate challenge relative to their control group (Figure S2E). These experiments do not exclude an additional contribution to fasting hypoketonemia by the depletion of adipose tissues, which was particularly pronounced in the cachectic relative to the food-restricted pre-cachectic groups (Figures S1D, S1F, and S1I), but they correspond directly to findings from models of PPARalpha deletion and hepatic PPARalpha dysfunction (Chakravarthy et al., 2005; Kersten et al., 1999; Sengupta et al., 2010). Taken together, these findings demonstrate that the ketogenic potential of the liver is impaired in pre-cachectic mice, most likely because of suppressed *Ppara* expression, and that this tumor-induced metabolic reprogramming exacerbates metabolic stress during subsequent periods of caloric deficiency.

IL-6 Is Necessary and Sufficient to Suppress Hepatic Ketogenesis in Pre-cachectic Mice

To investigate the mechanistic basis of tumor-induced suppression of hepatic *Ppara* and ketogenesis, we first performed a screen of tumor-associated cytokines and chemokines in the plasma of C26- and PDA-bearing mice. Given that the tumor-induced suppression of hepatic *Ppara* and ketogenesis was observed even in pre-cachectic C26- and PDA-bearing mice (Figures 2A–2D), we reasoned that the tumor-associated cytokine that accounted for these effects would be elevated in both pre-cachectic and cachectic mice from each model system. Of the cytokines that we screened, only IL-6 fulfilled this criterion (Figures 3A and S3A–S3C). Although TNFα was elevated in pre-cachectic and cachectic mice with PDA, plasma levels of this cytokine were unchanged in mice bearing C26 tumors. We also observed significantly elevated IL-6 levels in pre-cachectic and cachectic patients with PDA (Figure 3B). Importantly, this observation is consistent with the data from C26- and

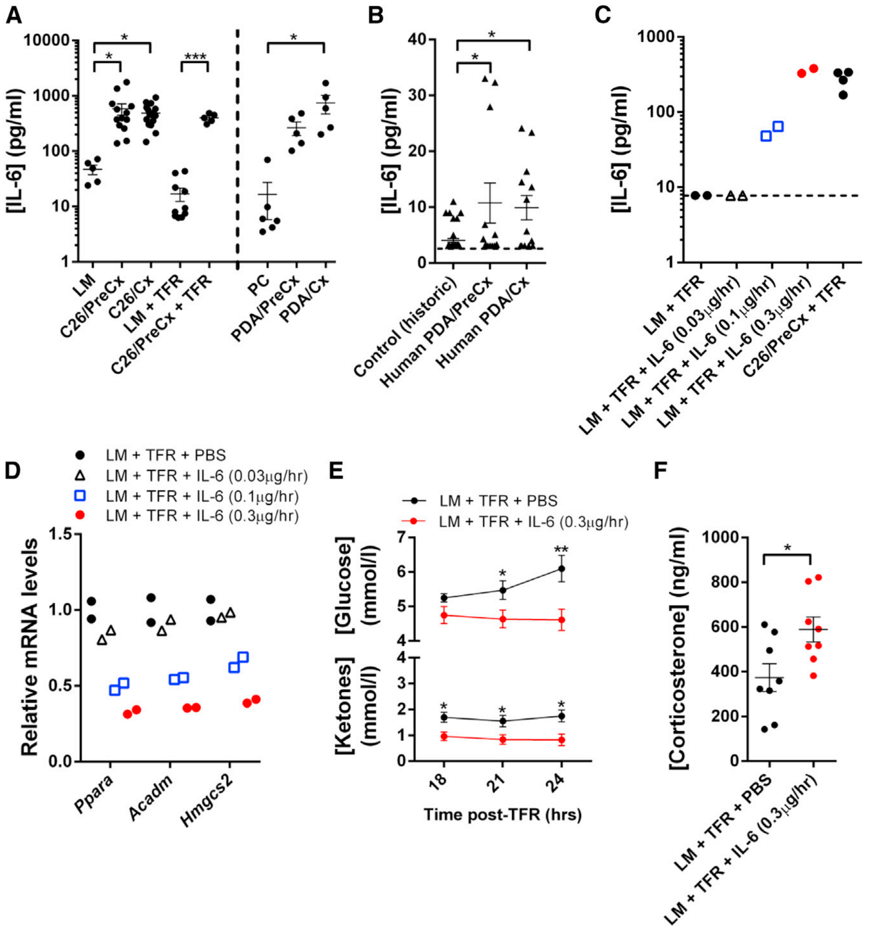

Figure 3. Reprogramming of the Hepatic Response to Caloric Deficiency by IL-6

(A) Plasma levels of IL-6 were measured in LM, C26/PreCx, C26/Cx, LM + TFR, C26/PreCx + TFR, PC, PDA/PreCx, and PDA/Cx mice.

(B) IL-6 serum levels in pre-cachectic and cachectic patients with PDA, as well as levels of historical control volunteers (as reported by the assay manufacturer), are displayed.

(C and D) Escalating doses of IL-6 were infused into non-tumor-bearing LM mice for 72 hr, with the final 24 hr under TFR. (C) IL-6 plasma levels and (D) hepatic mRNA levels for *Ppara*, *Acadm*, and *Hmgcs2* were assessed, the latter by qRT-PCR.

(E and F) LM mice were administered 0.3 μg/hr IL-6 for 72 hr, with the final 24 hr under the condition of TFR. Tail vein bleeds were assessed for (E) glucose and ketone levels 18–24 hr post-TFR (n = 8 per group) and for (F) corticosterone levels 18 hr post-TFR.

The measurements from (A) LM, C26/PreCx, and C26/Cx mice; (A) PC, PDA/PreCx, and PDA/Cx mice; as well as (B) control, pre-cachectic, and cachectic patients were compared using one-way ANOVA with Tukey's correction for post hoc comparisons. Comparisons of data at each time point in (E), of data in (F), and the comparison of LM +TFR versus C26/PreCx + TFR in (A) were performed using two-tailed t tests with Welch's correction. The dotted line in (B) and (C) represents the assay detection limit. $^{*}p < 0.05$, $^{**}p < 0.01$, $^{***}p < 0.001$. Data are presented as mean ± SEM.

PDA-bearing mice outlined above (Figure 3A), as well as with previous reports of elevated IL-6 levels in patients with PDA and with other cachexia-associated cancers (Staal-van den Brekel et al., 1995; Fearon et al., 1991; Okada et al., 1998).

To determine whether IL-6 contributed to the regulation of hepatic *Ppara* expression and ketogenesis, we infused recombinant IL-6 or PBS into non-tumor-bearing littermate mice for 72 hr, with the highest rate of infusion achieving plasma IL-6 levels comparable to those occurring in pre-cachectic and cachectic C26- and PDA-bearing mice (Figure 3C). During the final 24 hr, we subjected the mice to TFR. The recombinant IL-6 dose-dependently suppressed hepatic mRNA levels for *Ppara*, *Acadm*, and *Hmgcs2* and lowered fasting glucose and ketone levels while elevating fasting corticosterone levels (Figures 3D–3F). These changes were observed in the absence of IL-6-dependent alterations in body weight kinetics, body composition, and changes in pre-fasting plasma corticosterone levels (Figures S3D–S3F).

We then determined whether the elevated IL-6 in tumor-bearing mice accounted for the suppression of hepatic *Ppara* and ketogenesis by administering neutralizing anti-IL-6 antibody to pre-cachectic mice with C26 tumors for 72 hr, during which TFR was imposed for the final 24 hr. Anti-IL-6 administration partially restored not only the hepatic mRNA levels of *Ppara*, *Acadm*, and *Hmgcs2*, but also the ketogenic response to the octanoate challenge (Figures 4A and 4B). These changes were also associated with an improved metabolic response to fasting, as reflected by the normalized plasma levels of glucose, ketones, and corticosterone (Figures 4C and 4D). Anti-IL-6 administration did not change body weight kinetics, body composition, tumor growth, serum markers of hepatocellular damage, or pre-fasting food intake (Figures S4A–S4G).

The results from the above experiments clarify some important aspects underlying the suppression of hepatic ketogenesis and elevated corticosterone levels observed in our pre-cachectic model systems following food restriction. The reduced ketogenesis cannot be ascribed to reduced fat stores (Figures S1D, S1F, and S1I) because both gain- and loss-of-function experiments involving IL-6 were performed with mice that were initially matched for fat stores, and there were no IL-6-dependent alterations of fat mass (Figures S3E and S4E). Likewise, although IL-6 has previously been reported as a direct activator of the hypothalamic-pituitary-adrenal axis (Wang and Dunn, 1998), this mechanism cannot account for the corticosterone elevations that were observed in the C26 and PDA model systems under food restriction (Figure 1E). Pre-cachectic C26- and PDA-bearing mice exhibited normal corticosterone levels despite raised IL-6 levels (Figures 1E and 3A), and IL-6 infusion did not elevate corticosterone in the absence of food restriction (Figure S3F). Taken together, these data support the conclusion that tumor-induced IL-6 is both necessary and sufficient to suppress the potential of the liver for ketogenesis. This metabolic

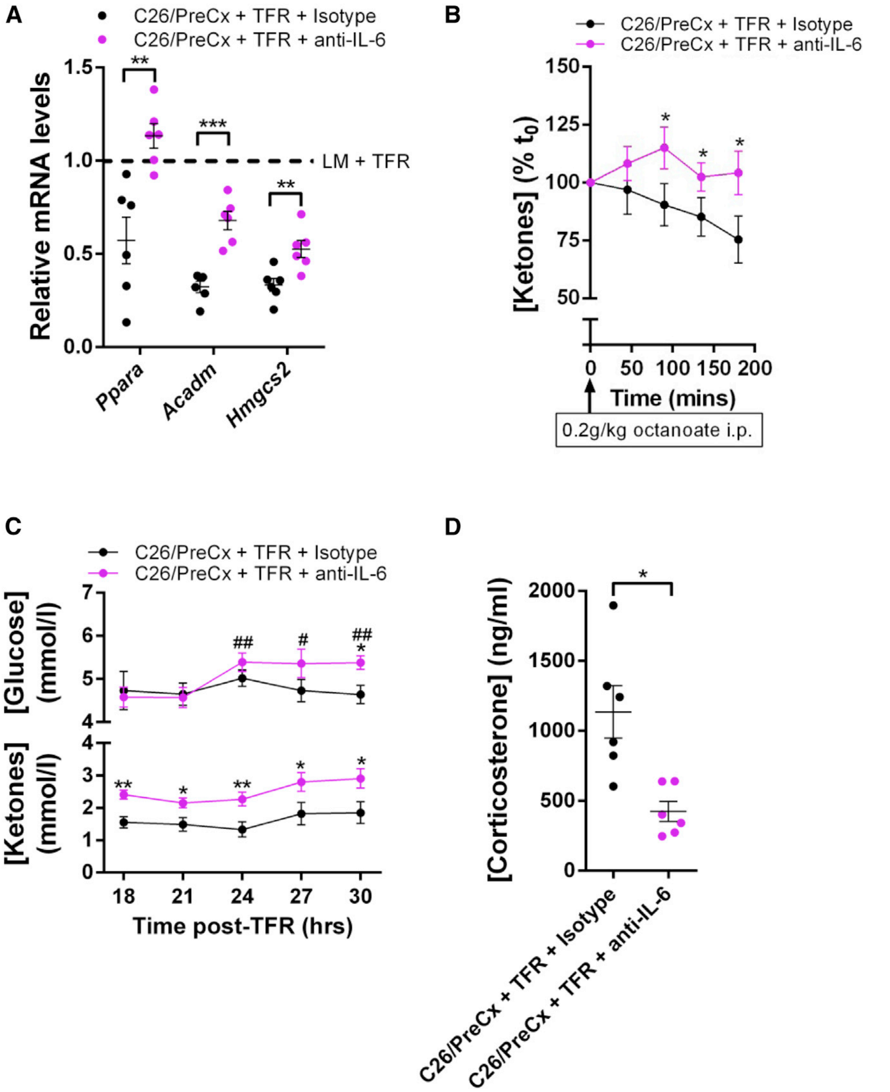

Figure 4. Neutralizing IL-6 Reverses Hepatic Reprogramming in C26-Bearing Mice

(A) C26/PreCx mice were administered isotype control or neutralizing anti-IL-6 antibodies 48 hr prior to and at the initiation of TFR. Hepatic mRNA levels for *Ppara* and its target genes were measured by qRT-PCR 24 hr post-TFR.

(B–D) Ketogenic response to octanoate 24 hr post-TFR (n = 7 per group) (B), glucose and ketone levels from tail bleeds 18–30 hr post-TFR (n = 10 per group) (C), and terminal bleed plasma corticosterone levels 24 hr post-TFR (D) were assessed.

Data comparisons in (A) and (D) as well as comparisons at each time point in (C) were performed using two-tailed t tests with Welch's correction. *$p < 0.05$, **$p < 0.01$, ***$p < 0.001$. Within-group comparisons of (C) glucose data were performed relative to the 18 hr time point using ratio paired t tests (#). For the (B) octanoate challenge, mice from the two groups were stratified by body weight prior to enrollment and the percent changes in blood ketones were compared by ratio paired t tests at each time point. Data are presented as mean ± SEM.

switch, however, does not affect glucocorticoid levels until the imposition of caloric deficiency, where hepatic ketogenesis is required to support the energy demands of the brain (Cahill, 2006). The circumstance of caloric deficiency leads to the metabolic stress that induces the marked glucocorticoid response (Figures 1E, 3F, and 4D).

Tumor-Induced Metabolic Stress Is Coupled with Suppressed Intratumoral Immunity

We addressed the possibility that the cachexia-associated alterations of host metabolism (Figures 1, 2, S1, and S2) might affect tumor biology by comparing the transcriptomes of C26 tumors taken from pre-cachectic and cachectic mice. Unsupervised analysis of the RNA sequencing (RNA-seq) data distinguished the tumors taken from pre-cachectic and cachectic C26-bearing mice (Figure S5A), and between-group comparisons yielded 2,973 differentially expressed genes at a false discovery rate (FDR) < 0.05 (Figure S5B). Downregulation in the tumors from cachectic mice was the dominant phenomenon, and of the 30 most downregulated pathways identified by a gene set enrichment analysis (GSEA) (FDR ≤ 0.001 for each of the 30 pathways), 29 were related to either innate or adaptive immunity (Figure 5A). No significantly upregulated pathways (FDR < 0.25) were identified by the analysis. A MetaCore enrichment analysis yielded similar results (Figures S5C and S5E). A review of the list of differentially expressed genes indicated that the dominant pathway alterations resulted from reduction of multiple immune cell types, including the CD4+, CD8+, and natural killer (NK) lymphocyte populations, in C26 tumors from cachectic mice (Table S1). Also reduced were transcripts for molecules involved in lymphocyte chemotaxis (*Cxcl9–11*), and CD4+ Th1 and CD8+ T cell effector function (*Ifng*, *Gzmb*, and *Prf1*). The myeloid compartment was also affected in these C26 tumors from cachectic mice, as demonstrated by a decrease in the levels of transcripts for *Itgam*, *Itgax*, and *Cd74* (CD11b, CD11c, and MHC II, respectively) (Table S1). The cachexia-associated depletion of intratumoral CD3+ T cells was confirmed by immunohistochemistry (Figure S5D). Flow cytometric analysis using markers for lymphoid cells was performed on C26 tumors from independent cohorts of pre-cachectic and cachectic mice (Figures 5B and S5F). In addition to the cachexia-associated loss of CD3+ T cells, this analysis demonstrated the depletion of CD8+ T cells, CD4+Foxp3− T cells, and NK cells. A trend toward reduced numbers of CD4+Foxp3+ T cells was also observed (Figure 5B).

We determined whether the immunological phenotype of C26 tumors taken from cachectic mice could be induced by food restriction of pre-cachectic C26-bearing mice. Since adaptive immune control of tumor growth depends on the intratumoral accumulation and function of T cells (Mikucki et al., 2015; Tumeh

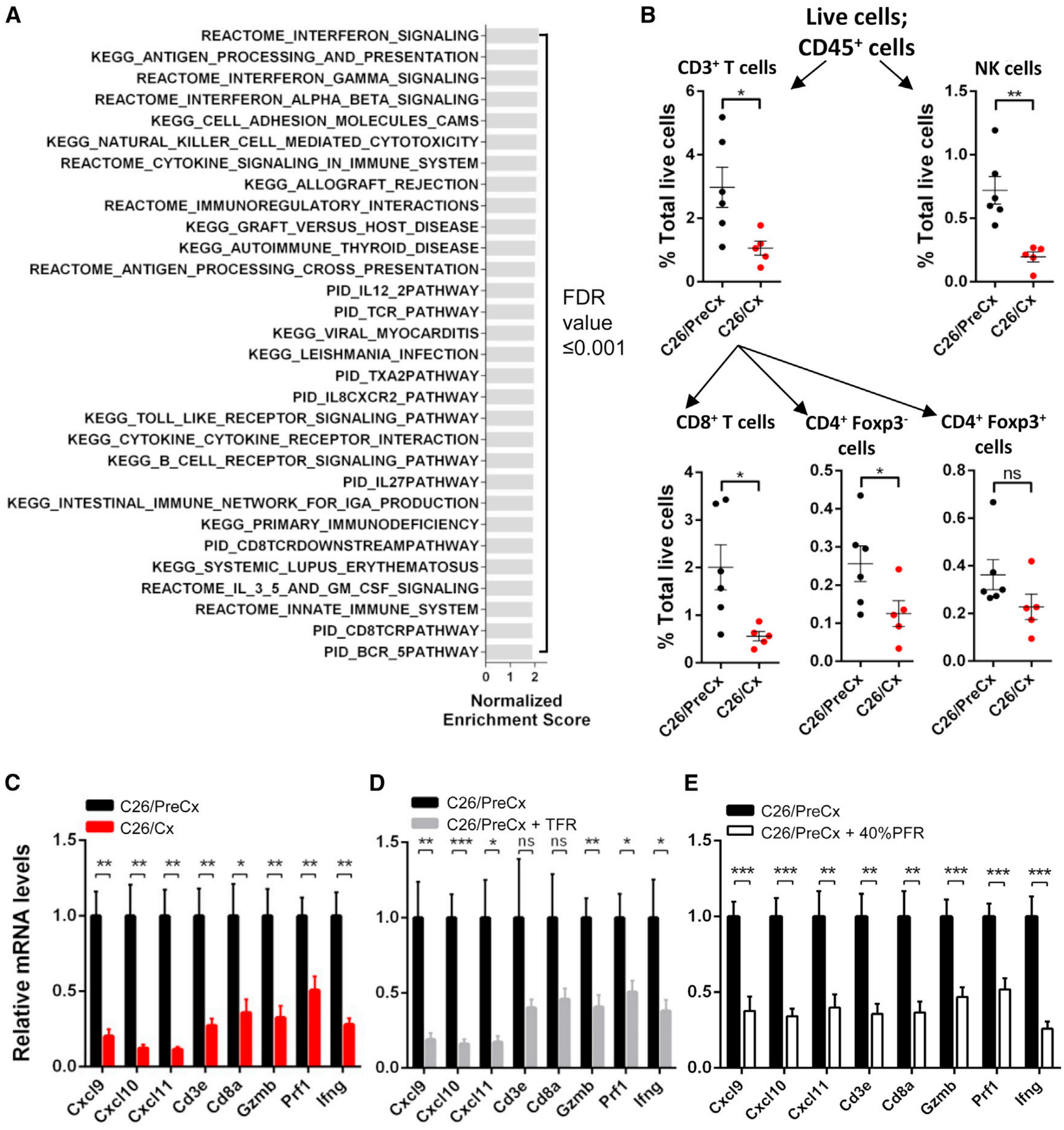

Figure 5. Suppression of Intratumoral Immunity in Cachexia and Caloric Deficiency

(A) RNA-seq data from whole-tumor lysates from C26/PreCx and C26/Cx mice were subjected to GSEA. The 30 most significantly downregulated gene sets in tumors from C26/Cx mice are presented. There were no significantly upregulated gene sets in tumors from C26/Cx mice at FDR < 0.25.

(B) The CD3+, CD8+, CD4+Foxp3−, CD4+Foxp3+, and NK cell populations of tumors from C26/PreCx and C26/Cx mice were enumerated using flow cytometry.

(C–E) Levels of intratumoral transcripts of genes relevant to T cell-mediated immunity were determined via qRT-PCR of tumors taken from (C) C26/PreCx mice, C26/Cx mice, (D) C26/PreCx + TFR mice, and (E) C26/PreCx mice subjected to 3 days of 40% partial food restriction (C26/PreCx + 40% PFR) (n = 6–12 per group).

The indicated data comparisons in (B)–(E) were performed using two-tailed t tests with Welch's correction. *$p < 0.05$, **$p < 0.01$, ***$p < 0.001$. Data are presented as mean ± SEM.

et al., 2014), we measured the expression of a panel of eight genes relating to these immune phenomena that were suppressed in tumors from cachectic mice: the CXCR3-dependent chemotaxis of T cells (*Cxcl9*, *Cxcl10*, and *Cxcl11*), the presence of T cells (*Cd8a* and *Cd3e*), and their effector functions (*Gzmb*, *Prf1*, and *Ifng*). Food-restricted pre-cachectic C26-bearing mice exhibited reductions in the expression of this immune gene panel that were similar to those that were observed in cachectic C26-bearing mice (Figures 5C and 5D). Forty percent partial food restriction, relative to baseline food intake, of pre-cachectic C26-bearing mice also induced metabolic stress, as reported by elevated plasma corticosterone levels; suppressed the expression of the selected immunological genes; and reduced the numbers of intratumoral CD3+ T cells relative to ad libitum-fed pre-cachectic C26-bearing mice (Figures 5E and S6A–S6E). Taken together, these findings demonstrate that the

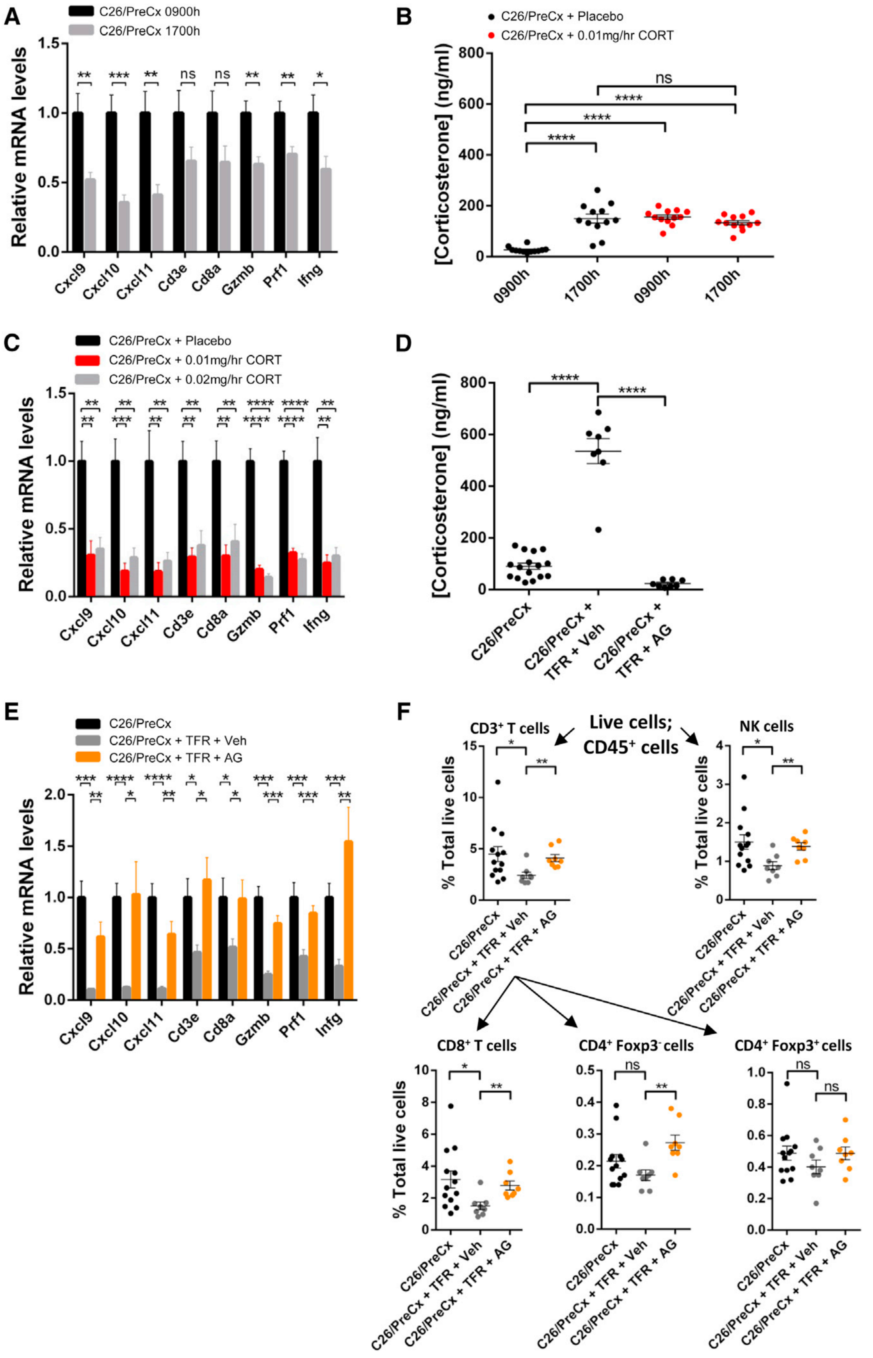

Figure 6. Glucocorticoids Connect Reprogrammed Hepatic Metabolism to Suppressed Intratumoral Immunity

(A) mRNA levels for immunological genes were measured via qRT-PCR in tumors taken from C26/PreCx mice at 0900 and 1700 hr (n = 12 per group).

(B) C26-bearing mice were implanted with subcutaneous pellets releasing 0.01 mg/hr corticosterone, or with placebo pellets, and plasma corticosterone levels were measured at 0900 and 1700 hr on day 7 of the infusion via tail vein bleeds.

(C) C26-bearing mice were implanted with placebo pellets, or pellets eluting either 0.01 mg/hr or 0.02 mg/hr corticosterone. Intratumoral mRNA levels of immunological genes were measured via qRT-PCR (n = 4–5 per group).

(D–F) C26/PreCx mice were subjected to TFR and administered the glucocorticoid synthesis inhibitor, aminoglutethimide, or vehicle control. Tail bleed corticosterone levels (D), intratumoral mRNA levels of immunological genes as measured via qRT-PCR (n = 8–13 per group) (E), and flow cytometric analyses of lymphoid subpopulations (F) are displayed. The C26/PreCx 0900 hr versus C26/PreCx 1700 hr, C26/PreCx versus C26/PreCx + TFR + vehicle, and C26/PreCx + TFR + vehicle versus C26/PreCx + TFR + aminoglutethimide comparisons were all performed using two-tailed t tests with Welch's correction.

Data in (B) were analyzed using two-way ANOVA with Tukey's correction for post hoc comparisons. Comparisons in (C) were performed using one-way ANOVA with Fisher's LSD (least significant difference) test for post hoc comparisons. *p < 0.05, **p < 0.01, ***p < 0.001, ****p < 0.0001. Data are presented as mean ± SEM. Veh, vehicle; AG, aminoglutethimide.

metabolic stress of caloric deficiency, whether spontaneous or experimentally induced, in C26-bearing mice suppresses intratumoral T cell immunity.

Glucocorticoids, Induced by Metabolic Stress, Suppress Intratumoral Immunity

The cachexia-associated phenotypes of reprogrammed hepatic metabolism with metabolic stress (Figures 1, 2, 3, and 4) and suppressed intratumoral immunity (Figures 5 and S5) were both induced by food restriction of pre-cachectic mice. We considered the possibility that the two responses were linked by the marked stress-induced elevations in glucocorticoids (Figures 1E, 3F, 4D, and S2D) because glucocorticoids are clinically used as potent pharmacological immune suppressants. We first assessed whether the immune gene panel of the C26 tumor in freely feeding pre-cachectic mice was sensitive to physiological, diurnal variation in plasma corticosterone levels. Expression of six of the eight genes in the panel was significantly lower in C26 tumors taken at 1700 hr, as compared to their expression in tumors taken at 0900 hr, which correlated with the peak and nadir, respectively, of the plasma corticosterone levels (Figures 6A and 6B). The possibility that these immune changes reflected a marked sensitivity of the C26 tumor microenvironment to corticosterone was examined by implanting subcutaneous pellets continuously releasing corticosterone at 0.01 mg/hr for 7 days in C26-bearing mice, which resulted in plasma corticosterone levels at 0900 and 1700 hr that were equivalent to the peak diurnal level (Figure 6B). This modest alteration in physiological corticosterone levels was sufficient to induce the phenotype of cachexia-associated

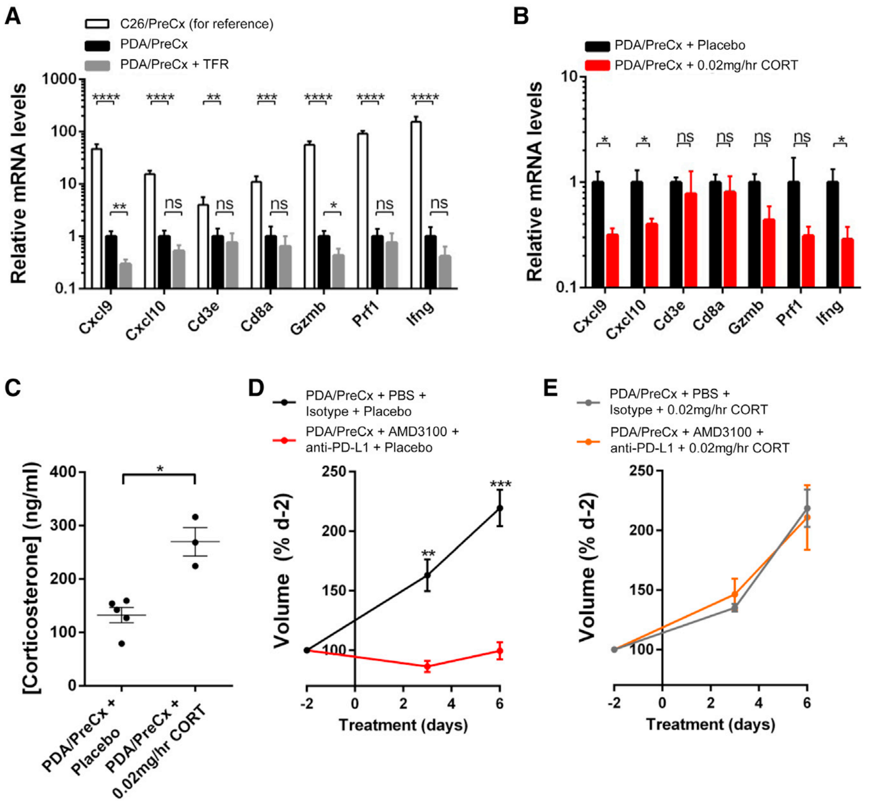

Figure 7. Glucocorticoids Suppress Intratumoral Immunity and Cause Failure of Cancer Immunotherapy in Autochthonous Mouse PDA

(A and B) Pre-cachectic PDA-bearing mice were subjected to (A) TFR (n = 8–9 per group) or (B) 6 day infusions with subcutaneous pellets releasing 0.02 mg/hr corticosterone (n = 4–5 per group). Intratumoral mRNA levels for immunological genes were measured via qRT-PCR. The mRNA levels in the tumors from the C26/PreCx mice from Figure 5D are normalized to the levels from the PDA/PreCx group.

(C) Tail bleed corticosterone levels from the corticosterone pellet-infused PDA/PreCx mice are displayed.

(D and E) PDA mice were administered AMD3100 + anti-PD-L1, or PBS + isotype control antibody, and implanted with (D) placebo pellets or (E) pellets releasing 0.02 mg/hr corticosterone. Tumor volumes were assessed by ultrasound over a 6 day treatment period.

The indicated statistical comparisons in (A)–(C), and comparisons at each time point in (D) and (E), were performed using two-tailed t tests with Welch's correction. Data in (A) and (B) were logarithmically transformed prior to statistical analysis. *$p < 0.05$, **$p < 0.01$, ***$p < 0.001$, ****$p < 0.0001$. Data are presented as mean ± SEM.

intratumoral immune suppression when assessed transcriptionally and histologically (Figures 6C and S6F). Increasing the corticosterone pellet release to 0.02 mg/hr dose dependently led to higher plasma corticosterone levels (354.5 ± 17.7 ng/mL SEM) but did not further suppress the expression of the immune gene panel or the intratumoral CD3+ T cell percentages (Figures 6C and S6F). Flow cytometric analysis of tumors from an independent cohort of C26-bearing mice that were implanted with the 0.01 mg/hr corticosterone pellets confirmed depletion of CD3+ T cells, CD8+ T cells, CD4+Foxp3− T cells, CD4+Foxp3+ T cells, and NK cells (Figure S6G). This marked sensitivity of the immune C26 tumor microenvironment to corticosterone was also reflected by the correlation between anti-IL-6-induced decreased plasma levels of the hormone and increased mRNA levels of *Cxcl9*, *Cxcl10*, and *Cxcl11* in food-restricted pre-cachectic C26-bearing mice (Figure S6H).

These experiments demonstrating the sensitivity of the immune microenvironment of the C26 tumors to changes in corticosterone levels did not exclude the possibility that other consequences of caloric deficiency and metabolic stress in tumor-bearing mice might contribute to immune suppression. To evaluate this possibility, pre-cachectic C26-bearing mice that were subjected to 24 hr TFR were administered the glucocorticoid synthesis inhibitor, aminoglutethimide, or vehicle control. Relative to freely feeding controls, the food-restricted, vehicle-treated group exhibited elevated plasma corticosterone levels, reduced expression of the intratumoral immune panel of genes, and fewer intratumoral T cells and NK cells (Figures 6D–6F and S7A). Trends toward fewer CD4+Foxp3− T cells and CD4+Foxp3+ T cells were also observed (Figure 6F). Aminoglutethimide inhibited the fasting-induced corticosterone production (Figure 6D) and all changes relating to intratumoral immunity (Figures 6E, 6F, and S7A), despite a further reduction in blood glucose levels (Figure S7B). These findings indicate that elevated corticosterone is the primary metabolic mediator of intratumoral immune suppression during caloric deficiency in pre-cachectic tumor-bearing mice.

Stress-Induced Glucocorticoids Cause Failure of Cancer Immunotherapy

Human PDA has not responded to therapy with antagonists of T cell checkpoints (Brahmer et al., 2012; Royal et al., 2010), and the mouse model of this disease is also resistant to antibodies specific for PD-1, PD-L1, and CTLA-4 (Feig et al., 2013; Zhu et al., 2014). Consistent with this lack of response of PDA to stimulators of T cell function, and consistent with the low numbers of intratumoral T cells predicting failure of immunotherapy (Tumeh et al., 2014), are the markedly lower levels of intratumoral transcripts relating to T cell immunity in pre-cachectic PDA-bearing mice as compared to their levels in pre-cachectic C26-bearing mice (Figure 7A). We examined whether this contrasting immune microenvironment also exhibited sensitivity to metabolic stress. Subjecting pre-cachectic PDA-bearing mice to 24 hr of TFR reduced expression of *Cxcl9* and *Gzmb*, with trends toward reduced expression for *Cxcl10* and *Ifng* (Figure 7A). Continuously elevating corticosterone levels in PDA-bearing mice for 6 days using a 0.02 mg/hr corticosterone infusion also suppressed the expression of *Cxcl9*, *Cxcl10*, and

Ifng, with trends toward reduced expression of *Gzmb* and *Prf1* (Figures 7B and 7C).

The potential clinical relevance of the sensitivity of the immune microenvironment of mouse PDA to glucocorticoids was assessed by examining the effect of elevated corticosterone levels on immunotherapy in this model. Pre-clinical experiments have demonstrated T cell-dependent control of PDA growth in mice receiving the selective CXCR4 antagonist, AMD3100, in combination with anti-PD-L1 (Feig et al., 2013). We treated freely feeding, weight-stable pre-cachectic PDA-bearing mice for 6 days with a continuous infusion of AMD3100 or PBS, in combination with anti-PD-L1 or isotype control antibody, in the context of subcutaneous pellets releasing corticosterone at a rate of 0.02 mg/hr or placebo control pellets. In mice with control pellets, this immunotherapy arrested PDA growth (Figure 7D). In contrast, in mice with corticosterone-releasing pellets that resulted in elevated plasma levels of the hormone, which were lower than those of cachectic or food-restricted pre-cachectic PDA-bearing mice (Figure 7C) and comparable to the fold elevations observed in human cachexia (Figure 1G), this immunotherapy failed to control PDA growth (Figure 7E). AMD3100 plasma levels were comparable in the placebo and corticosterone-infused groups (1,020 ± 63.0 ng/mL SEM versus 1,310 ± 161 ng/mL SEM, respectively). Through infusion of glucocorticoids at doses that are relevant both physiologically and clinically, we have thus demonstrated a direct link between the tumor-induced alteration in the metabolic response to caloric deprivation and failure of PDA tumors to respond to immunotherapy.

DISCUSSION

We have demonstrated in two mouse models of cancer-induced cachexia that in pre-cachectic mice, even before the onset of the weight-losing phase of the syndrome, tumor-induced IL-6 has altered the capacity of the liver to respond to caloric deprivation. Through a suppression of ketogenesis that is attributable to suppression of its transcriptional master regulator, PPARalpha, this tumor-induced reprogramming of hepatic metabolism has removed an important component of the host's capacity to make available endogenous sources of energy that compensate for decreased caloric intake. This compounding of the energy deficit magnifies the host stress response and leads to glucocorticoid levels that are more than sufficient to suppress tumor immunity, especially when considering the sensitivity of the immune tumor microenvironment even to diurnal variations of the hormone. This apparently paradoxical metabolic response to caloric deficiency may subvert therapeutic interventions designed to correct other causes of the failure of tumor immune surveillance, such as poor immunogenicity and local immune privilege (Joyce and Fearon, 2015; Le et al., 2015; Zelenay et al., 2015). The translational relevance of our study is emphasized by validation of all key aspects of the metabolic and immunological phenotypes, and performance of the immune therapy experiment, in the KPC autochthonous PDA model, which recapitulates human PDA and is the accepted model for pre-clinical studies of this cancer (Mayers et al., 2014; Rhim et al., 2014). In addition, this study demonstrates, in accordance with our murine data, that some patients with pancreatic cancer exhibit raised IL-6 levels when they are pre-cachectic, and that they may exhibit the triad of raised IL-6 levels, reduced ketone levels, and raised glucocorticoid levels when they are cachectic.

The finding that tumor-induced IL-6 suppresses PPARalpha-regulated ketogenesis was confirmed mechanistically, in vivo, through both gain- and loss-of-function studies (Figures 3C–3F, 4A–4D, S3D–S3F, and S4A–S4G). This ketogenic deficit was shown to be independent of decreased fat mass (Figures S3E and S4E) and, importantly, to be induced by infusing IL-6 in the absence of a tumor (Figures 3C–3F). The mechanism whereby IL-6 impinged upon the expression of PPARalpha in the liver is not addressed by our study, although as PPARalpha regulates its own transcription (Mandard et al., 2004), suppressed expression may relate to deprivation of the physiological ligand for PPARalpha (Chakravarthy et al., 2005) or repression of the peroxisome proliferator response element (PPRE) (Sengupta et al., 2010). The finding that tumor-induced IL-6 was both necessary and sufficient to suppress hepatic ketogenesis, taken together with the fact that consistent elevations of other cytokines were not detected in our model systems, indicates that the effects on hepatic metabolism of other cytokines are less relevant in this metabolic context. Finally, we have not addressed the possibility that the fatty acids that are liberated from adipose tissue and not readily metabolized by reprogrammed livers may be a source of macronutrients to be used by cancer cells for growth (Kamphorst et al., 2013). Nevertheless, the observation of tumor-induced IL-6 reprogramming hepatic ketogenesis, when taken together with the recent report of lung cancer rewiring hepatic circadian homeostasis involving insulin, glucose, and lipid metabolism (Masri et al., 2016), suggests that the liver may be a specific target of tumor-induced effects on the host, presumably for the benefit of the tumor and the detriment of the host.

The results relating to hepatic metabolism alone may have multiple clinical implications. Hepatic ketone body production is essential in order to spare glucose and support brain function during periods of caloric insufficiency (Cahill, 2006). The responses of blood ketones and glucose in fasted pre-cachectic mice to the exogenous fatty acid substrate, octanoate, were directionally opposed relative to the responses of non-tumor-bearing controls (Figures 2D and S2E), indicating severely compromised nutrient processing, and perhaps explaining why conventional nutritional support has so far been ineffective in cachectic cancer patients (Tisdale, 2009). Such aberrant liver metabolism may not be limited to the context of cancer: the hepatic effect of IL-6 in tumor-free mice is dose dependent across the range observed in tumor-bearing mice (Figures 3A, 3C, and 3D), implicating the process in a wide range of IL-6-associated diseases where weight loss is observed, such as sepsis, HIV, tuberculosis, chronic obstructive pulmonary disease, cardiac failure, and rheumatoid arthritis. In terms of therapeutic reversibility, our body composition data following acute IL-6 neutralization are consistent with existing clinical data regarding the failure of anti-IL-6 to improve wasting parameters in cachectic patients without concomitant correction of caloric deficiencies (Fearon et al., 2012). When taken with the more subtle effects on metabolism, and in particular the rescue of the octanoate response (Figure 4B), these data suggest that successful reversal of

cachexia may require co-administration of anti-IL-6 with nutritional support.

In the context of caloric deficiency, where ketogenesis is normally activated to provide energy substrates that are essential to support brain function (Cahill, 2006), IL-6-induced metabolic reprogramming leads directly to the phenotype of glucocorticoid-induced immune suppression within the tumor microenvironment. Initially observed in an unbiased comparison of the transcriptomes of the C26 tumors in cachectic and pre-cachectic mice, and then shown to be a consequence of the compensatory glucocorticoid response to caloric deficiency, this immunological phenotype exhibited an internally consistent cluster of findings: decreased levels of *Cxcl9*, *Cxcl10*, and *Cxcl11* mRNA in association with fewer lymphocytes and diminished mRNA levels of the lymphocyte effector genes, *Ifng*, *Gzmb*, and *Prf1* (Figures 5A–5E, 6A–6F, S6A–S6G, and S7A). We focused our attention on these markers because CXCR3 signaling is required for T cell infiltration into tumor sites (Mikucki et al., 2015), and the relative intensity of intratumoral T cell infiltration predicts both cancer survival and the response to anti-cancer immunotherapy in humans (Galon et al., 2006; Rooney et al., 2015; Tumeh et al., 2014). The transcriptional findings were recapitulated in the PDA model system (Figures 7A and 7B). Glucocorticoid sensitivity was thus observed in tumor immune microenvironments with high and low levels of immunological activity. This is translationally relevant because tumors from patients with cancer exhibit a wide spectrum of immunological activity (Rooney et al., 2015). Among the many potential mechanisms mediating the immunosuppressive effects of glucocorticoids, the diminished expression of *Cxcl9–11* suggests that the most relevant cellular target of the elevated glucocorticoids may be the intratumoral myelomonocytic cell. Deletion of NR3C1, the glucocorticoid receptor, in these cells has been shown to abolish glucocorticoid-dependent suppression of the expression of *Cxcl10* and other chemotactic factors in a contact hypersensitivity reaction (Tuckermann et al., 2007).

A potential biological role for this metabolic pathway may be to suppress immunological damage to inflamed, non-infected tissues since many inflammatory conditions are associated with anorexia, high IL-6 levels, and elevated serum glucocorticoids. In the context of inflammation associated with cancer, however, this metabolic pathway subverts the host's capacity to mediate immune control of the cancer. Providing exogenous corticosterone in freely feeding pre-cachectic PDA-bearing mice isolated the effects of this hormone from other alterations associated with cachexia, thereby permitting an evaluation of its effects on anti-tumor immunotherapy. Corticosterone levels that were below the range observed in cachectic or food-restricted pre-cachectic mice were sufficient to abolish the anti-tumor effect of dual CXCR4 and PD-L1 antagonism in mice bearing autochthonous PDA (Figures 7C–7E). Cachexia is a frequent occurrence in human pancreatic cancer (Bachmann et al., 2013), associated with fold elevations of glucocorticoids that are comparable to those achieved during the corticosterone infusions (Figures 1G, 6B, and 7C), and known to be reversible upon tumor shrinkage or removal (Fearon et al., 2013; Strassmann et al., 1992). These observations, together with the recently initiated clinical trials involving CXCR4 antagonism with or without co-targeting of the PD-1:PD-L1 axis in PDA (NCT02179970; NCT02472977), attest to the clinical relevance of our findings.

In conclusion, we have unveiled a new aspect of how metabolism in tumor-bearing hosts is reprogrammed in the pre-cachectic state. Such metabolic dysregulation can ultimately lead to failure of host immunity to control cancer in the context of anti-cancer immunotherapy. Prior resolution of such deranged metabolism through a combination of IL-6 neutralization and hyperalimentation may be necessary in order to maximize the proportion of patients that respond to these therapies. Rescue of immunotherapeutic efficacy may also be achieved in a more direct manner, through inhibition of glucocorticoid synthesis. In turn, glucocorticoid levels themselves may be of use as biomarkers in the context of immunotherapy. Future clinical studies of both cachexia-modulating interventions and anti-cancer immune therapies may need to take these findings into consideration in order to optimize the selection of therapeutic combinations and the discovery of biomarkers.

EXPERIMENTAL PROCEDURES

Full experimental procedures are provided in the Supplemental Experimental Procedures.

Animal Experiments

All experiments were performed in accordance with national and institutional guidelines and approved by the UK Home Office, the animal ethics committee of Cancer Research UK Cambridge Institute, and the University of Cambridge. The C26 model experiments were performed on wild-type male BALB/c mice, which were kept on a 24 hr 12:12 light-dark cycle and inoculated with $2*10^6$ viable cells subcutaneously (s.c.). Mice were termed pre-cachectic from 18 days post-inoculation. Cachexia was defined as >5% loss from peak body weight.

PDA was detected via ultrasound in KPC mice. Mice with maximum average PDA tumor diameters >4 mm were defined as pre-cachectic.

i.p. injections were administered as follows: anti-IL-6 (MP5.20F3) and isotype IgG1 (HRPN) at 48 hr prior to and at the point of TFR (1.25 mg per injection); sodium octanoate at 24 hr post-TFR (6 mL/kg of 200 mM); aminoglutethimide at 0, 8, and 16 hr post-TFR (37.5 mg/kg per injection); and anti-PD-L1 on days 0, 2, and 4 (200 μg per injection). Corticosterone or placebo pellets and osmotic minipumps loaded with AMD3100, recombinant IL-6, or PBS were implanted s.c.

Murine Blood and Plasma Measurements

Blood samples were kept on ice. Plasma and tissues were snap frozen in liquid nitrogen and stored at −80°C. Tail bleeds were analyzed for glucose and ketone concentrations using gluco- and ketometers. Corticosterone was quantified using ELISA. Terminal cardiac bleed plasma glucose and ketone levels were assessed using the Siemens Dimension RxL analyzer and the Stanbio Beta Hydroxybutyrate Liquicolor kit, respectively. Insulin, leptin, and cytokines were measured using MesoScale Discovery kits. IL-6 levels were measured using ELISA.

AMD3100 was extracted from plasma samples pre-spiked with internal standard (D4-labeled AMD3100) and EDTA by protein precipitation with methanol. Quantitation was by liquid chromatography-tandem mass spectrometry (LC-MS/MS) over a calibration range of 50–5,000 ng/mL.

qRT-PCR

mRNA was extracted from snap-frozen tissues using TRIzol Reagent. mRNA templates were diluted to 2 ng/μL (muscle and liver) or 30 ng/μL (tumor) and analyzed by quantitative real-time PCR. mRNA levels were normalized to either Rn18s (liver and tumor) or Tbp (quadriceps) using the ddCt method.

RNA-Seq

RNA extracted from frozen tissues via TRIzol was run through QIAGEN RNeasy columns following the “RNA cleanup” protocol. Integrity was confirmed using RIN values with a cut-off of 8, and libraries were prepared using the Illumina TruSeq mRNA Stranded Sample prep kit (96 index high throughput) using twelve rounds of PCR. Libraries were quantity and quality checked and normalized. The final pooled library was run on a MiSeq to assess final sequencing quality before HiSeq 2500 V4 single-end 50 bp sequencing. We aimed to generate 10–20 M single-end 50 bp reads per sample.

For the analysis, single-end 50 bp reads were aligned to the mouse genome version GRCm38.74 using TopHat v2.0.4. Read counts were obtained using HTSeq-count v0.5.3p9. Read counts were normalized and tested for differential gene expression using the Bioconductor package DESeq v1.10.1. Multiple testing correction was applied using the Benjamini-Hochberg method. The data can be accessed at http://dx.doi.org/10.17863/CAM.4930.

GSEA was performed by ranking all genes tested in RNA-seq using $-\log_{10}$ (p values) derived from differential expression analyses and testing against MSigDB Canonical Pathways (C2:CP). MetaCore enrichment analyses gated on the significantly (FDR < 0.05) up- and downregulated genes were also performed using MetaCore’s pathway maps database.

Immunohistochemistry

Tissues were fixed in 10% neutral buffered formaldehyde. Immunohistochemistry was performed for CD3 using a Leica Bond III immunostainer. Deparaffinization and rehydration were conducted before antigen retrieval was performed using Tris EDTA (pH 9) (ER2) for 20 min at 100°C, incubation in a rabbit polyclonal anti-CD3 antibody (A0452) for 15 min, HRP-linked anti-rabbit polymer for 8 min, diaminobenzidine for 10 min, and DAB Enhancer (Leica) for 10 min. Counterstaining was with hematoxylin for 2 min. The slides were scanned at 20× on a Leica AT2 and subsequently analyzed in a blinded manner using the Cytonuclear v1.4 algorithm on the HALO platform.

Flow Cytometry

C26 tumors were mechanically and enzymatically homogenized using buffer containing RPMI with 1 mg/mL collagenase (Sigma C0130) and 0.1 mg/mL DNase (Sigma D4527). Cells were stained for flow cytometry according to reagent manufacturers’ protocols. Viability was determined using eBioscience e780 fixable viability dye at a 1/1,000 dilution. All antibodies are listed in the Supplemental Experimental Procedures. CD49b positivity was determined using a fluorescence-minus-one control. Anti-CD16/32 (unconjugated; 2.4G2; 5 μg/mL; BD) was used for Fc blocking prior to antibody staining. The eBioscience Foxp3/Transcription Factor Fixation/Permeabilization Concentrate and Diluent was used prior to Foxp3 staining. Sample analysis was performed using an LSR II cytometer. At least 200,000 events per tumor sample were collected. Data were subsequently analyzed using FlowJo.

Human Studies

The study and the sample acquisition were performed in concordance with local and national guidelines. Patients at least 18 years old with histologically confirmed pancreatic adenocarcinoma were recruited in the outpatient department of the Cambridge University Hospital NHS Foundation Trust as part of the CAMPAN study. All participants provided written informed consent. All samples were taken between 1300 and 1500 hr. Patients were designated cachectic if they met the international consensus definition for cachexia, i.e., if they exhibited weight loss of >5% over past 6 months (in the absence of simple starvation) or BMI < 20 and ongoing weight loss of more than 2% (Fearon et al., 2011). Serum cortisol levels were assessed using the Cortisol Parameter Assay kit from R&D Systems. Serum IL-6 levels were assessed using ELISA. Ketone levels from plasma were assessed using Freestyle Optium Neo ketometers (Abbott Laboratories).

Statistical Analysis

Statistical analyses, unless otherwise indicated, were performed using GraphPad Prism 6.

SUPPLEMENTAL INFORMATION

Supplemental Information includes Supplemental Experimental Procedures, seven figures, and one table and can be found with this article online at http://dx.doi.org/10.1016/j.cmet.2016.10.010.

AUTHOR CONTRIBUTIONS

Conceptualization, T.R.F., T.J., and D.T.F; Methodology, T.R.F. and T.J.; Formal Analysis, T.R.F., T.J., and D.T.F.; Investigation, T.R.F. and T.J.; C.M.C., E.W.R., and A.E.D. assisted with experiments; Resources, T.R.F., T.J., D.I.J., and D.T.F.; Writing – Original Draft, T.R.F., T.J., and D.T.F.; Writing – Review & Editing, T.R.F., T.J., A.P.C., D.I.J. and D.T.F.; Visualization, T.R.F. and T.J.; Funding Acquisition, T.R.F., T.J., D.I.J., and D.T.F.;

ACKNOWLEDGMENTS

We thank all patients. We also thank the University of Cambridge, Cancer Research UK, the CRUK Cambridge Institute Core Facilities, and Hutchison Whampoa Limited. This work was also supported by the Lustgarten Foundation for Pancreatic Cancer Research, the Ludwig Institute for Cancer Research, the NIHR Biomedical Research Centre, and the Cambridge ECMC. T.R.F. was supported by the Rosetrees Trust and the Cambridge School of Clinical Medicine’s MB/PhD Programme, T.J. was supported by the Wellcome Trust Translational Medicine and Therapeutics Programme and the University of Cambridge Department of Oncology (RJAG/076), C.M.C. was supported by the Cambridge University Hospitals NHS Foundation Trust, E.W.R. was supported by the CRI Irvington Postdoctoral Fellowship Program, and A.P.C. was supported by the Medical Research Council (MRC) Metabolic Diseases Unit (MRC_MC_UU_12012/1). D.T.F. is a Distinguished Scholar of the Lustgarten Foundation.

Received: December 9, 2015
Revised: September 12, 2016
Accepted: October 17, 2016
Published: November 8, 2016

REFERENCES

Amiel, S.A., Archibald, H.R., Chusney, G., Williams, A.J., and Gale, E.A. (1991). Ketone infusion lowers hormonal responses to hypoglycaemia: evidence for acute cerebral utilization of a non-glucose fuel. Clin. Sci. *81*, 189–194.

Bachmann, J., Büchler, M.W., Friess, H., and Martignoni, M.E. (2013). Cachexia in patients with chronic pancreatitis and pancreatic cancer: impact on survival and outcome. Nutr. Cancer *65*, 827–833.

Brahmer, J.R., Tykodi, S.S., Chow, L.Q.M., Hwu, W.-J., Topalian, S.L., Hwu, P., Drake, C.G., Camacho, L.H., Kauh, J., Odunsi, K., et al. (2012). Safety and activity of anti-PD-L1 antibody in patients with advanced cancer. N. Engl. J. Med. *366*, 2455–2465.

Cahill, G.F., Jr. (2006). Fuel metabolism in starvation. Annu. Rev. Nutr. *26*, 1–22.

Chakravarthy, M.V., Pan, Z., Zhu, Y., Tordjman, K., Schneider, J.G., Coleman, T., Turk, J., and Semenkovich, C.F. (2005). “New” hepatic fat activates PPARalpha to maintain glucose, lipid, and cholesterol homeostasis. Cell Metab. *1*, 309–322.

Chang, C.-H., Qiu, J., O’Sullivan, D., Buck, M.D., Noguchi, T., Curtis, J.D., Chen, Q., Gindin, M., Gubin, M.M., van der Windt, G.J.W., et al. (2015). Metabolic competition in the tumor microenvironment is a driver of cancer progression. Cell *162*, 1229–1241.

Ellacott, K.L.J., Morton, G.J., Woods, S.C., Tso, P., and Schwartz, M.W. (2010). Assessment of feeding behavior in laboratory mice. Cell Metab. *12*, 10–17.

Fearon, K.C., McMillan, D.C., Preston, T., Winstanley, F.P., Cruickshank, A.M., and Shenkin, A. (1991). Elevated circulating interleukin-6 is associated with an acute-phase response but reduced fixed hepatic protein synthesis in patients with cancer. Ann. Surg. *213*, 26–31.

Fearon, K., Strasser, F., Anker, S.D., Bosaeus, I., Bruera, E., Fainsinger, R.L., Jatoi, A., Loprinzi, C., MacDonald, N., Mantovani, G., et al. (2011). Definition and classification of cancer cachexia: an international consensus. Lancet Oncol. *12*, 489–495.

Fearon, K.C.H., Glass, D.J., and Guttridge, D.C. (2012). Cancer cachexia: mediators, signaling, and metabolic pathways. Cell Metab. *16*, 153–166.

Fearon, K., Arends, J., and Baracos, V. (2013). Understanding the mechanisms and treatment options in cancer cachexia. Nat. Rev. Clin. Oncol. *10*, 90–99.

Feig, C., Jones, J.O., Kraman, M., Wells, R.J.B., Deonarine, A., Chan, D.S., Connell, C.M., Roberts, E.W., Zhao, Q., Caballero, O.L., et al. (2013). Targeting CXCL12 from FAP-expressing carcinoma-associated fibroblasts synergizes with anti-PD-L1 immunotherapy in pancreatic cancer. Proc. Natl. Acad. Sci. USA *110*, 20212–20217.

Galon, J., Costes, A., Sanchez-Cabo, F., Kirilovsky, A., Mlecnik, B., Lagorce-Pagès, C., Tosolini, M., Camus, M., Berger, A., Wind, P., et al. (2006). Type, density, and location of immune cells within human colorectal tumors predict clinical outcome. Science *313*, 1960–1964.

Hingorani, S.R., Wang, L., Multani, A.S., Combs, C., Deramaudt, T.B., Hruban, R.H., Rustgi, A.K., Chang, S., and Tuveson, D.A. (2005). Trp53R172H and KrasG12D cooperate to promote chromosomal instability and widely metastatic pancreatic ductal adenocarcinoma in mice. Cancer Cell *7*, 469–483.

Ho, P.-C., Bihuniak, J.D., Macintyre, A.N., Staron, M., Liu, X., Amezquita, R., Tsui, Y.-C., Cui, G., Micevic, G., Perales, J.C., et al. (2015). Phosphoenolpyruvate is a metabolic checkpoint of anti-tumor T cell responses. Cell *162*, 1217–1228.

Joyce, J.A., and Fearon, D.T. (2015). T cell exclusion, immune privilege, and the tumor microenvironment. Science *348*, 74–80.

Kalaany, N.Y., and Sabatini, D.M. (2009). Tumours with PI3K activation are resistant to dietary restriction. Nature *458*, 725–731.

Kamphorst, J.J., Cross, J.R., Fan, J., de Stanchina, E., Mathew, R., White, E.P., Thompson, C.B., and Rabinowitz, J.D. (2013). Hypoxic and Ras-transformed cells support growth by scavenging unsaturated fatty acids from lysophospholipids. Proc. Natl. Acad. Sci. USA *110*, 8882–8887.

Kersten, S., Seydoux, J., Peters, J.M., Gonzalez, F.J., Desvergne, B., and Wahli, W. (1999). Peroxisome proliferator-activated receptor alpha mediates the adaptive response to fasting. J. Clin. Invest. *103*, 1489–1498.

Keusch, G.T., and Farthing, M.J.G. (1986). Nutrition and infection. Annu. Rev. Nutr. *6*, 131–154.

Kir, S., White, J.P., Kleiner, S., Kazak, L., Cohen, P., Baracos, V.E., and Spiegelman, B.M. (2014). Tumour-derived PTH-related protein triggers adipose tissue browning and cancer cachexia. Nature *513*, 100–104.

Le, D.T., Wang-Gillam, A., Picozzi, V., Greten, T.F., Crocenzi, T., Springett, G., Morse, M., Zeh, H., Cohen, D., Fine, R.L., et al. (2015). Safety and survival with GVAX pancreas prime and Listeria Monocytogenes-expressing mesothelin (CRS-207) boost vaccines for metastatic pancreatic cancer. J. Clin. Oncol. *33*, 1325–1333.

Mandard, S., Müller, M., and Kersten, S. (2004). Peroxisome proliferator-activated receptor alpha target genes. Cell. Mol. Life Sci. *61*, 393–416.

Masri, S., Papagiannakopoulos, T., Kinouchi, K., Liu, Y., Cervantes, M., Baldi, P., Jacks, T., and Sassone-Corsi, P. (2016). Lung adenocarcinoma distally rewires hepatic circadian homeostasis. Cell *165*, 896–909.

Mayers, J.R., Wu, C., Clish, C.B., Kraft, P., Torrence, M.E., Fiske, B.P., Yuan, C., Bao, Y., Townsend, M.K., Tworoger, S.S., et al. (2014). Elevation of circulating branched-chain amino acids is an early event in human pancreatic adenocarcinoma development. Nat. Med. *20*, 1193–1198.

McGarry, J.D., and Foster, D.W. (1971). The regulation of ketogenesis from octanoic acid. The role of the tricarboxylic acid cycle and fatty acid synthesis. J. Biol. Chem. *246*, 1149–1159.

Mikucki, M.E., Fisher, D.T., Matsuzaki, J., Skitzki, J.J., Gaulin, N.B., Muhitch, J.B., Ku, A.W., Frelinger, J.G., Odunsi, K., Gajewski, T.F., et al. (2015). Non-redundant requirement for CXCR3 signalling during tumoricidal T-cell trafficking across tumour vascular checkpoints. Nat. Commun. *6*, 7458.

Okada, S., Okusaka, T., Ishii, H., Kyogoku, A., Yoshimori, M., Kajimura, N., Yamaguchi, K., and Kakizoe, T. (1998). Elevated serum interleukin-6 levels in patients with pancreatic cancer. Jpn. J. Clin. Oncol. *28*, 12–15.

Olive, K.P., Jacobetz, M.A., Davidson, C.J., Gopinathan, A., McIntyre, D., Honess, D., Madhu, B., Goldgraben, M.A., Caldwell, M.E., Allard, D., et al. (2009). Inhibition of Hedgehog signaling enhances delivery of chemotherapy in a mouse model of pancreatic cancer. Science *324*, 1457–1461.

Park, E.J., Lee, J.H., Yu, G.-Y., He, G., Ali, S.R., Holzer, R.G., Österreicher, C.H., Takahashi, H., and Karin, M. (2010). Dietary and genetic obesity promote liver inflammation and tumorigenesis by enhancing IL-6 and TNF expression. Cell *140*, 197–208.

Petruzzelli, M., Schweiger, M., Schreiber, R., Campos-Olivas, R., Tsoli, M., Allen, J., Swarbrick, M., Rose-John, S., Rincon, M., Robertson, G., et al. (2014). A switch from white to brown fat increases energy expenditure in cancer-associated cachexia. Cell Metab. *20*, 433–447.

Rhim, A.D., Oberstein, P.E., Thomas, D.H., Mirek, E.T., Palermo, C.F., Sastra, S.A., Dekleva, E.N., Saunders, T., Becerra, C.P., Tattersall, I.W., et al. (2014). Stromal elements act to restrain, rather than support, pancreatic ductal adenocarcinoma. Cancer Cell *25*, 735–747.

Roberts, E.W., Deonarine, A., Jones, J.O., Denton, A.E., Feig, C., Lyons, S.K., Espeli, M., Kraman, M., McKenna, B., Wells, R.J., et al. (2013). Depletion of stromal cells expressing fibroblast activation protein-α from skeletal muscle and bone marrow results in cachexia and anemia. J. Exp. Med. *210*, 1137–1151.

Rooney, M.S., Shukla, S.A., Wu, C.J., Getz, G., and Hacohen, N. (2015). Molecular and genetic properties of tumors associated with local immune cytolytic activity. Cell *160*, 48–61.

Royal, R.E., Levy, C., Turner, K., Mathur, A., Hughes, M., Kammula, U.S., Sherry, R.M., Topalian, S.L., Yang, J.C., Lowy, I., and Rosenberg, S.A. (2010). Phase 2 trial of single agent Ipilimumab (anti-CTLA-4) for locally advanced or metastatic pancreatic adenocarcinoma. J. Immunother. *33*, 828–833.

Sengupta, S., Peterson, T.R., Laplante, M., Oh, S., and Sabatini, D.M. (2010). mTORC1 controls fasting-induced ketogenesis and its modulation by ageing. Nature *468*, 1100–1104.

Siegel, R., Naishadham, D., and Jemal, A. (2012). Cancer statistics, 2012. CA Cancer J. Clin. *62*, 10–29.

Staal-van den Brekel, A.J., Dentener, M.A., Schols, A.M., Buurman, W.A., and Wouters, E.F. (1995). Increased resting energy expenditure and weight loss are related to a systemic inflammatory response in lung cancer patients. J. Clin. Oncol. *13*, 2600–2605.

Strassmann, G., Fong, M., Kenney, J.S., and Jacob, C.O. (1992). Evidence for the involvement of interleukin 6 in experimental cancer cachexia. J. Clin. Invest. *89*, 1681–1684.

Tanaka, Y., Eda, H., Tanaka, T., Udagawa, T., Ishikawa, T., Horii, I., Ishitsuka, H., Kataoka, T., and Taguchi, T. (1990). Experimental cancer cachexia induced by transplantable colon 26 adenocarcinoma in mice. Cancer Res. *50*, 2290–2295.

Tisdale, M.J. (2009). Mechanisms of cancer cachexia. Physiol. Rev. *89*, 381–410.

Tuckermann, J.P., Kleiman, A., Moriggl, R., Spanbroek, R., Neumann, A., Illing, A., Clausen, B.E., Stride, B., Förster, I., Habenicht, A.J.R., et al. (2007). Macrophages and neutrophils are the targets for immune suppression by glucocorticoids in contact allergy. J. Clin. Invest. *117*, 1381–1390.

Tumeh, P.C., Harview, C.L., Yearley, J.H., Shintaku, I.P., Taylor, E.J.M., Robert, L., Chmielowski, B., Spasic, M., Henry, G., Ciobanu, V., et al. (2014). PD-1 blockade induces responses by inhibiting adaptive immune resistance. Nature *515*, 568–571.

Wang, J., and Dunn, A.J. (1998). Mouse interleukin-6 stimulates the HPA axis and increases brain tryptophan and serotonin metabolism. Neurochem. Int. *33*, 143–154.

Zelenay, S., van der Veen, A.G., Böttcher, J.P., Snelgrove, K.J., Rogers, N., Acton, S.E., Chakravarty, P., Girotti, M.R., Marais, R., Quezada, S.A., et al. (2015). Cyclooxygenase-dependent tumor growth through evasion of immunity. Cell *162*, 1257–1270.

Zhou, X., Wang, J.L., Lu, J., Song, Y., Kwak, K.S., Jiao, Q., Rosenfeld, R., Chen, Q., Boone, T., Simonet, W.S., et al. (2010). Reversal of cancer cachexia and muscle wasting by ActRIIB antagonism leads to prolonged survival. Cell *142*, 531–543.

Zhu, Y., Knolhoff, B.L., Meyer, M.A., Nywening, T.M., West, B.L., Luo, J., Wang-Gillam, A., Goedegebuure, S.P., Linehan, D.C., and DeNardo, D.G. (2014). CSF1/CSF1R blockade reprograms tumor-infiltrating macrophages and improves response to T-cell checkpoint immunotherapy in pancreatic cancer models. Cancer Res. *74*, 5057–5069.

Cell Metabolism, Volume *24*

Supplemental Information

Tumor-Induced IL-6 Reprograms Host Metabolism to Suppress Anti-tumor Immunity

Thomas R. Flint, Tobias Janowitz, Claire M. Connell, Edward W. Roberts, Alice E. Denton, Anthony P. Coll, Duncan I. Jodrell, and Douglas T. Fearon

Figure S1

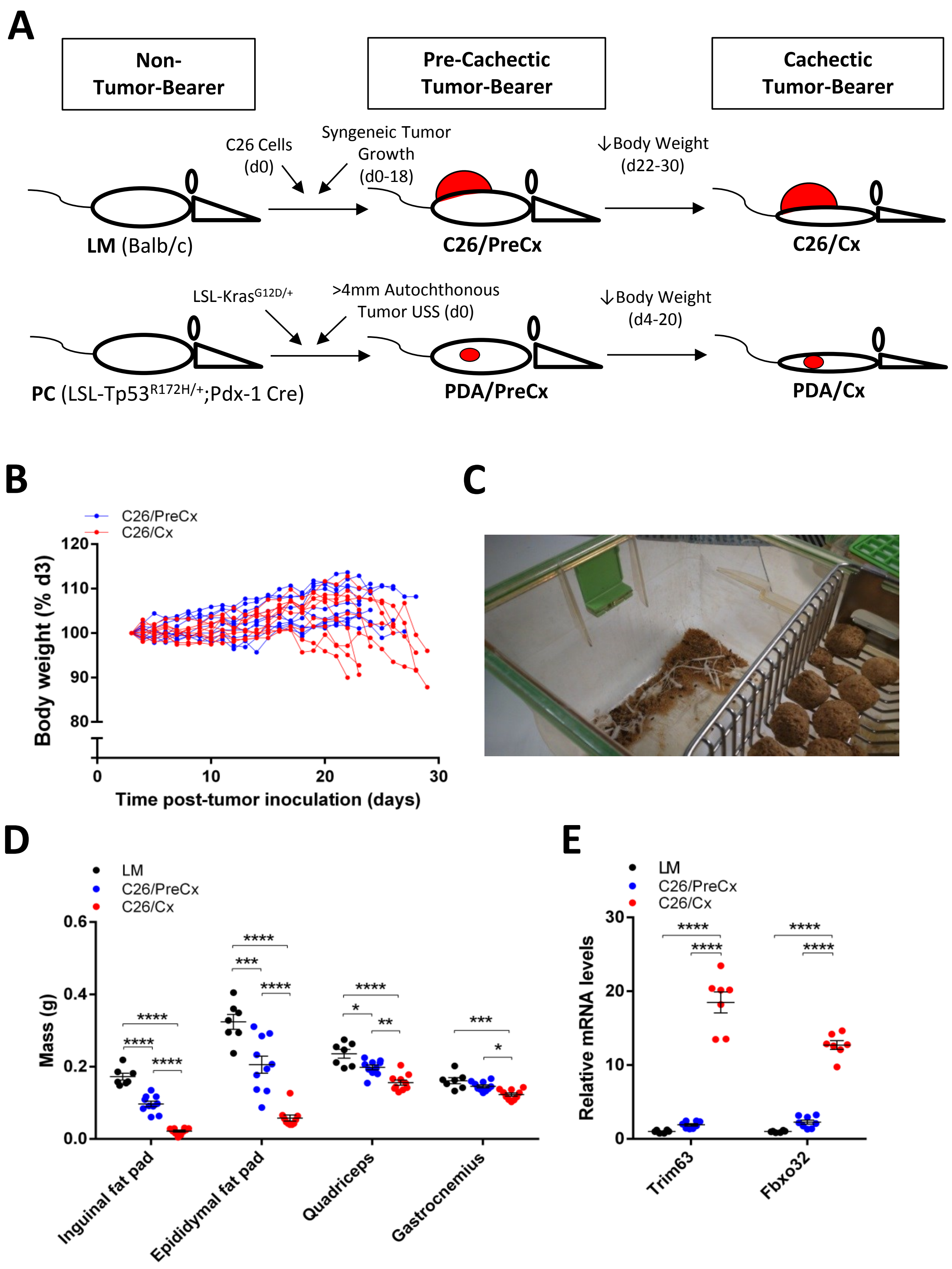
A
Non-Tumor-Bearer
Pre-Cachectic Tumor-Bearer
Cachectic Tumor-Bearer
C26 Cells (d0)
Syngeneic Tumor Growth (d0-18)
↓Body Weight (d22-30)
LM (Balb/c)
C26/PreCx
C26/Cx
LSL-Kras$^{G12D/+}$
>4mm Autochthonous Tumor USS (d0)
↓Body Weight (d4-20)
PC (LSL-Tp53$^{R172H/+}$;Pdx-1 Cre)
PDA/PreCx
PDA/Cx
B
C26/PreCx
C26/Cx
Body weight (% d3)
120
110
100
90
80
0
10
20
30
Time post-tumor inoculation (days)
C
D
LM
C26/PreCx
C26/Cx
Mass (g)
0.6
0.4
0.2
0.0
Inguinal fat pad
Epididymal fat pad
Quadriceps
Gastrocnemius
E
LM
C26/PreCx
C26/Cx
Relative mRNA levels
30
20
10
0
Trim63
Fbxo32

Figure S1

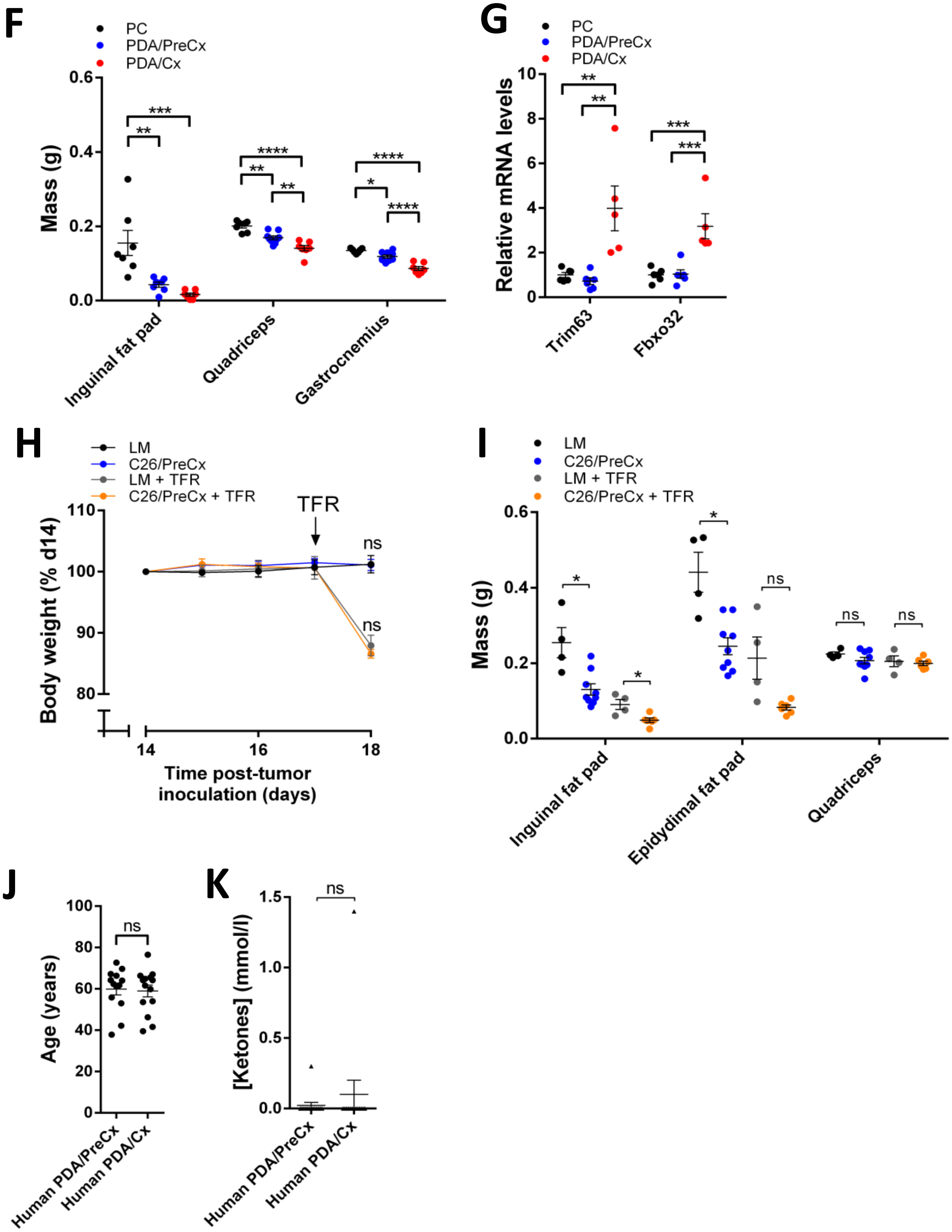

F
PC
PDA/PreCx
PDA/Cx
Mass (g)
0.6
0.4
0.2
0.0
Inguinal fat pad
Quadriceps
Gastrocnemius
G
Relative mRNA levels
Trim63
Fbxo32
H
LM
C26/PreCx
LM + TFR
C26/PreCx + TFR
TFR
ns
Body weight (% d14)
Time post-tumor inoculation (days)
I
Mass (g)
Inguinal fat pad
Epidydimal fat pad
Quadriceps
J
Age (years)
Human PDA/PreCx
Human PDA/Cx
K
[Ketones] (mmol/l)
Human PDA/PreCx
Human PDA/Cx

Figure S2

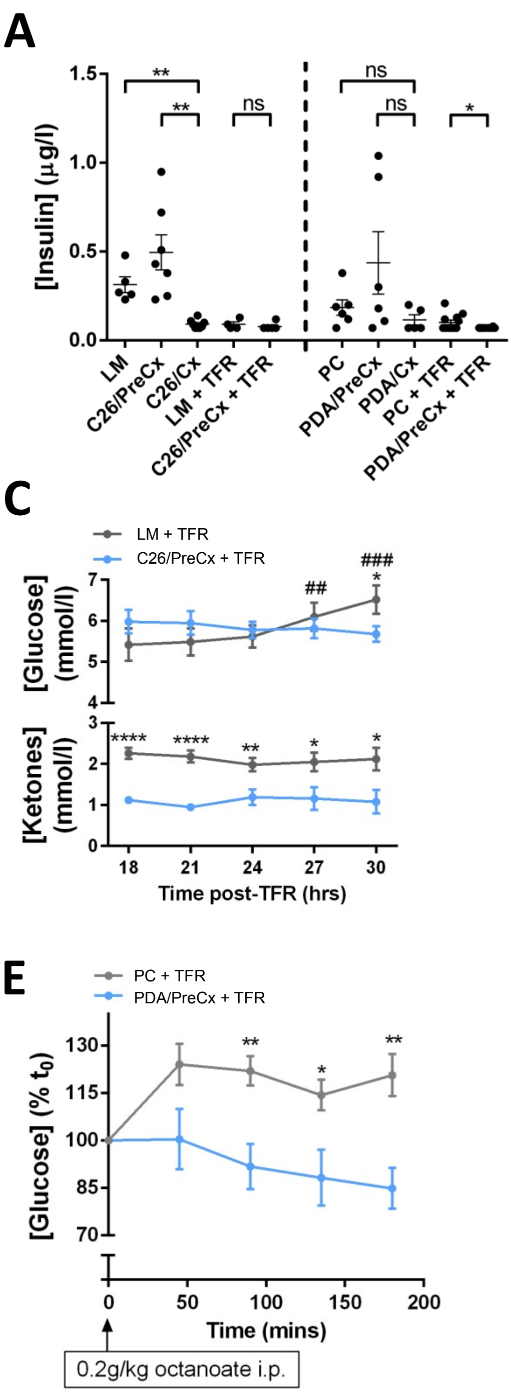
A
[Insulin] (μg/l)
LM
C26/PreCx
C26/Cx
LM + TFR
C26/PreCx + TFR
PC
PDA/PreCx
PDA/Cx
PC + TFR
PDA/PreCx + TFR
B
[Leptin] (pg/l)
C
LM + TFR
C26/PreCx + TFR
[Glucose] (mmol/l)
[Ketones] (mmol/l)
Time post-TFR (hrs)
D
[Corticosterone] (ng/ml)
Time post-TFR (hrs)
E
PC + TFR
PDA/PreCx + TFR
[Glucose] (% t0)
Time (mins)
0.2g/kg octanoate i.p.

Figure S3

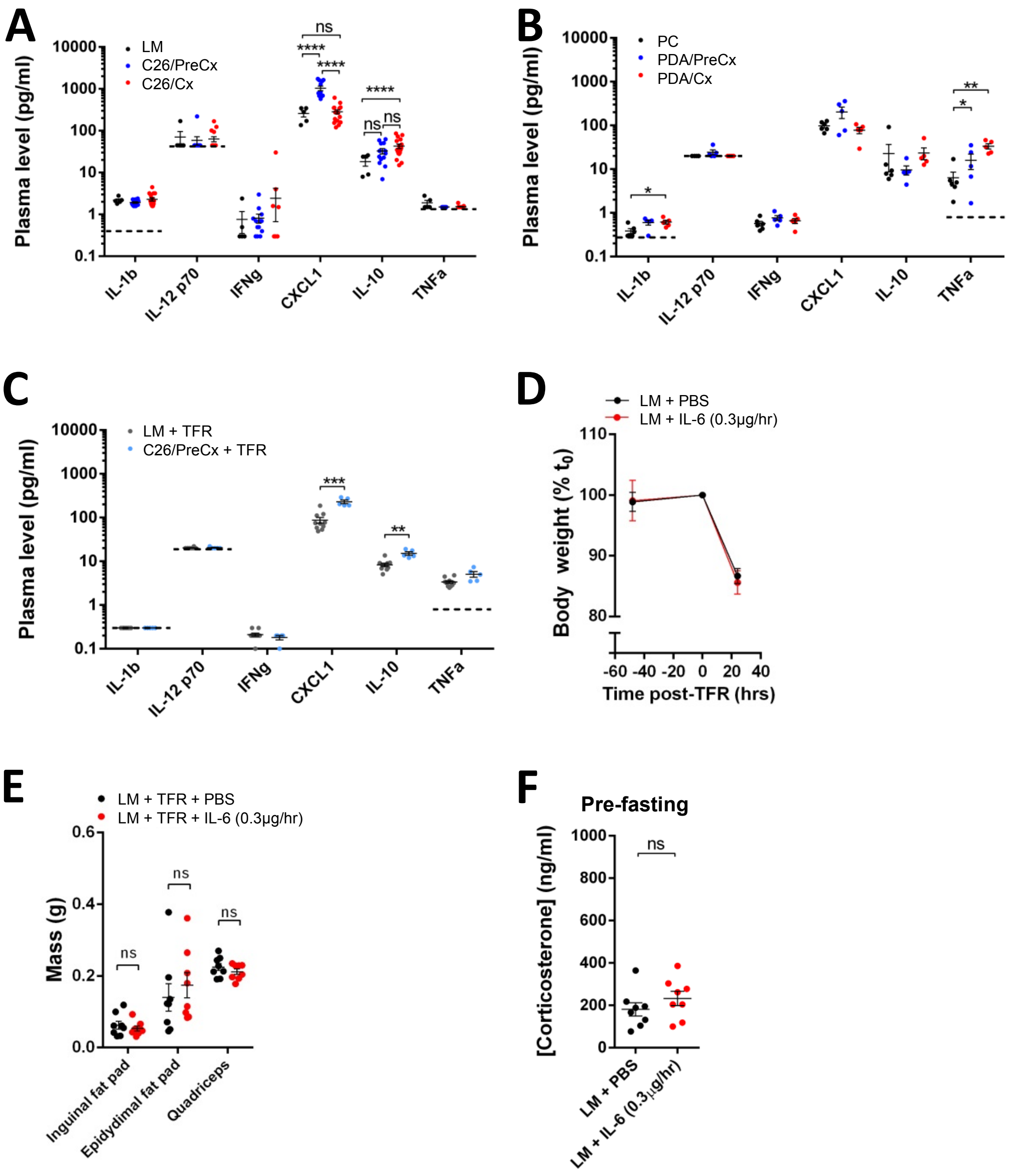
A
LM
C26/PreCx
C26/Cx
Plasma level (pg/ml)
IL-1b
IL-12 p70
IFNg
CXCL1
IL-10
TNFa
B
PC
PDA/PreCx
PDA/Cx
C
LM + TFR
C26/PreCx + TFR
D
LM + PBS
LM + IL-6 (0.3μg/hr)
Body weight (% t0)
Time post-TFR (hrs)
E
LM + TFR + PBS
LM + TFR + IL-6 (0.3μg/hr)
Mass (g)
Inguinal fat pad
Epidydimal fat pad
Quadriceps
F
Pre-fasting
[Corticosterone] (ng/ml)
LM + PBS
LM + IL-6 (0.3μg/hr)

Figure S4

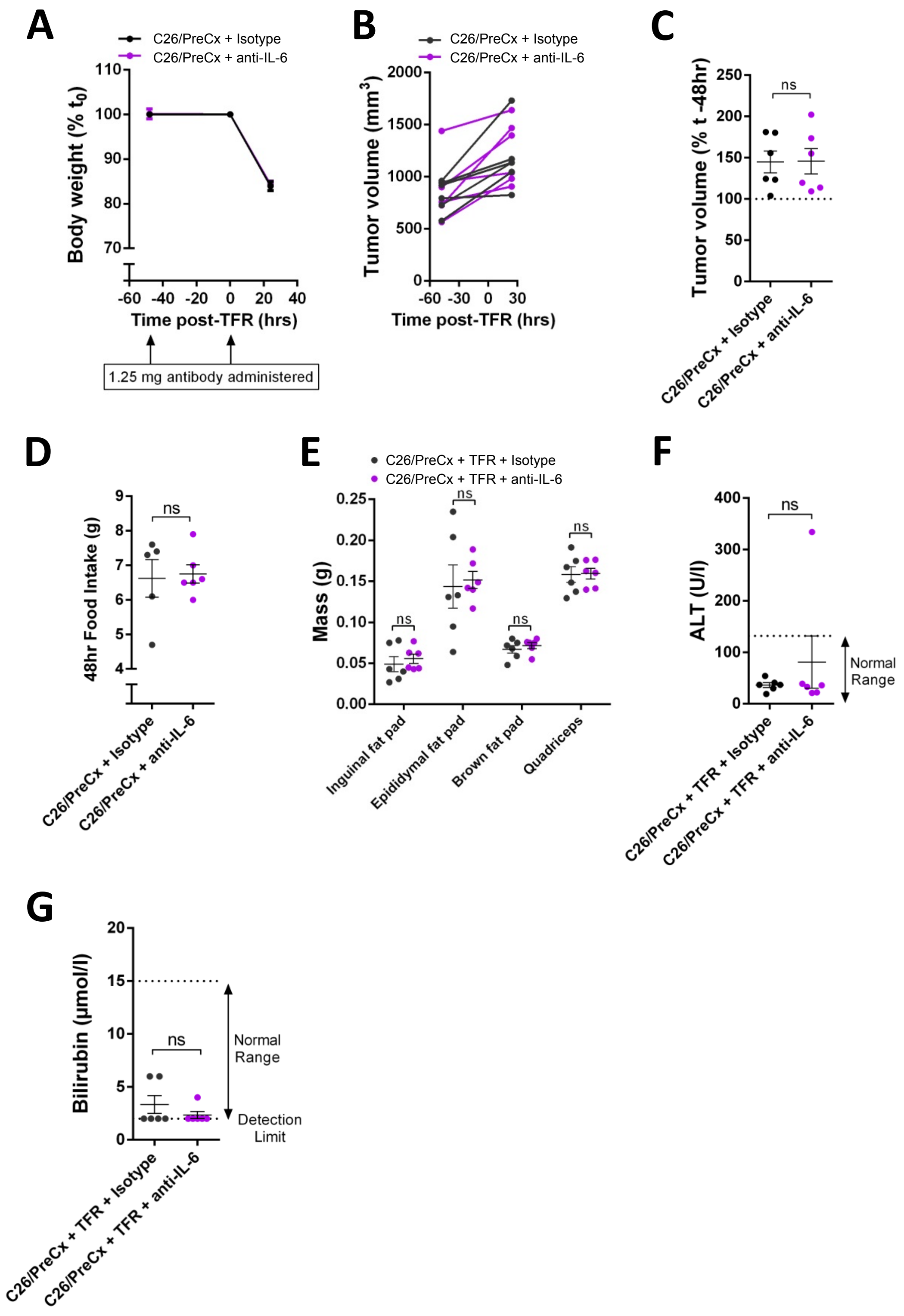
A
C26/PreCx + Isotype
C26/PreCx + anti-IL-6
Body weight (% t0)
Time post-TFR (hrs)
1.25 mg antibody administered
B
Tumor volume (mm3)
Time post-TFR (hrs)
C
Tumor volume (% t -48hr)
ns
D
48hr Food Intake (g)
E
C26/PreCx + TFR + Isotype
C26/PreCx + TFR + anti-IL-6
Mass (g)
Inguinal fat pad
Epididymal fat pad
Brown fat pad
Quadriceps
F
ALT (U/l)
Normal Range
C26/PreCx + TFR + Isotype
C26/PreCx + TFR + anti-IL-6
G
Bilirubin (μmol/l)
Normal Range
Detection Limit

Figure S5

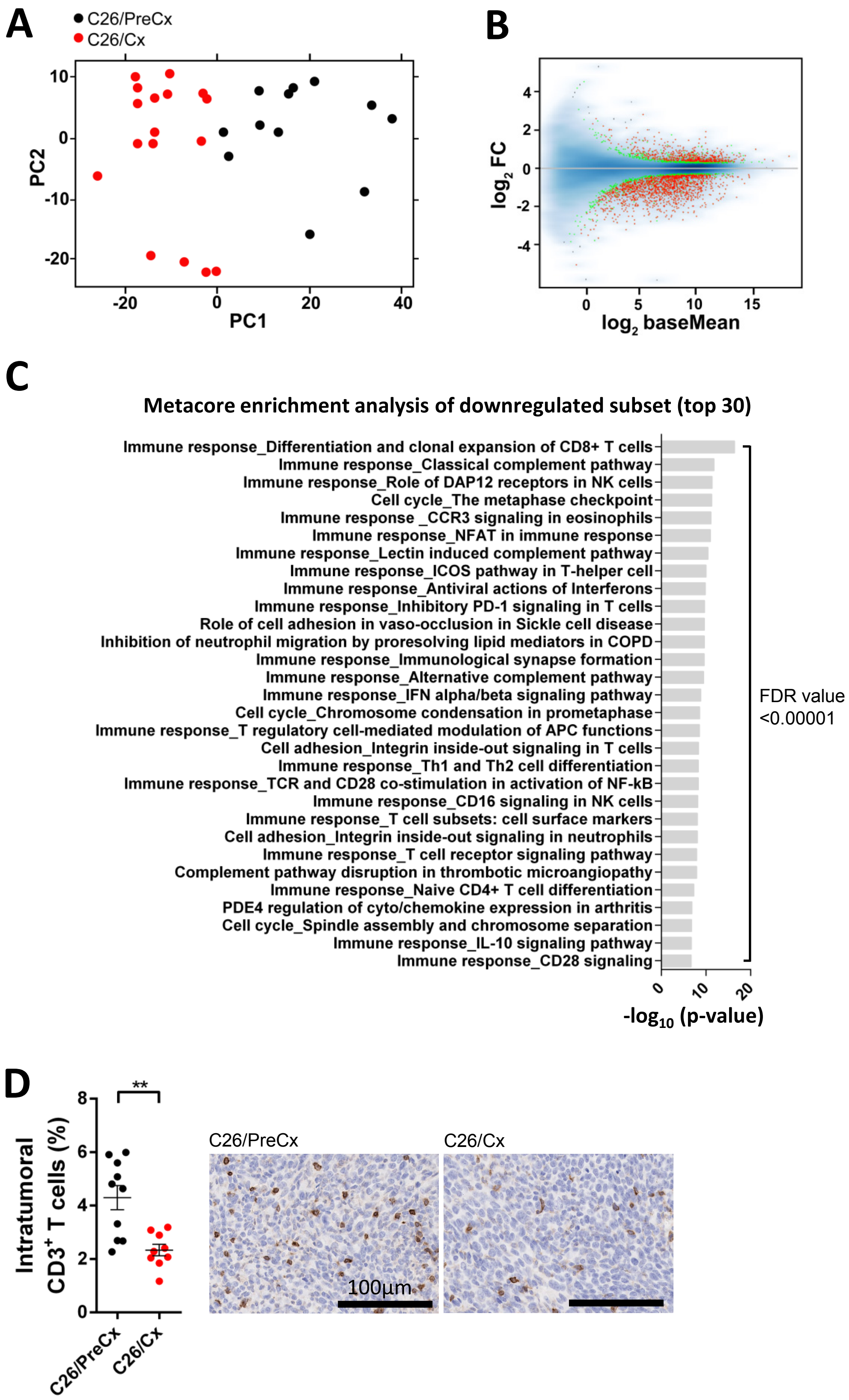
A
C26/PreCx
C26/Cx
PC2
PC1
10
0
-10
-20
-20
0
20
40
B
log2 FC
log2 baseMean
4
2
0
-2
-4
0
5
10
15
C
Metacore enrichment analysis of downregulated subset (top 30)
Immune response_Differentiation and clonal expansion of CD8+ T cells
Immune response_Classical complement pathway
Immune response_Role of DAP12 receptors in NK cells
Cell cycle_The metaphase checkpoint
Immune response _CCR3 signaling in eosinophils
Immune response_NFAT in immune response
Immune response_Lectin induced complement pathway
Immune response_ICOS pathway in T-helper cell
Immune response_Antiviral actions of Interferons
Immune response_Inhibitory PD-1 signaling in T cells
Role of cell adhesion in vaso-occlusion in Sickle cell disease
Inhibition of neutrophil migration by proresolving lipid mediators in COPD
Immune response_Immunological synapse formation
Immune response_Alternative complement pathway
Immune response_IFN alpha/beta signaling pathway
Cell cycle_Chromosome condensation in prometaphase
Immune response_T regulatory cell-mediated modulation of APC functions
Cell adhesion_Integrin inside-out signaling in T cells
Immune response_Th1 and Th2 cell differentiation
Immune response_TCR and CD28 co-stimulation in activation of NF-kB
Immune response_CD16 signaling in NK cells
Immune response_T cell subsets: cell surface markers
Cell adhesion_Integrin inside-out signaling in neutrophils
Immune response_T cell receptor signaling pathway
Complement pathway disruption in thrombotic microangiopathy
Immune response_Naive CD4+ T cell differentiation
PDE4 regulation of cyto/chemokine expression in arthritis
Cell cycle_Spindle assembly and chromosome separation
Immune response_IL-10 signaling pathway
Immune response_CD28 signaling
FDR value
<0.00001
0
10
20
-log10 (p-value)
D
**
8
6
4
2
0
Intratumoral
CD3+ T cells (%)
C26/PreCx
C26/Cx
C26/PreCx
C26/Cx
100μm

Figure S5

E

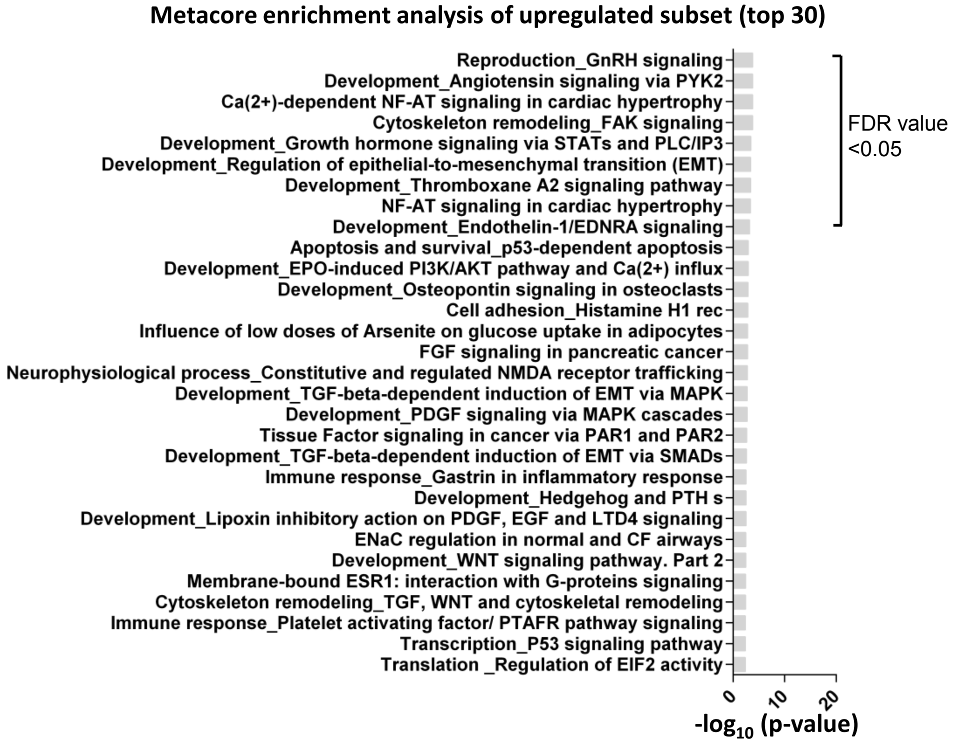

Metacore enrichment analysis of upregulated subset (top 30)
Reproduction_GnRH signaling
Development_Angiotensin signaling via PYK2
Ca(2+)-dependent NF-AT signaling in cardiac hypertrophy
Cytoskeleton remodeling_FAK signaling
Development_Growth hormone signaling via STATs and PLC/IP3
Development_Regulation of epithelial-to-mesenchymal transition (EMT)
Development_Thromboxane A2 signaling pathway
NF-AT signaling in cardiac hypertrophy
Development_Endothelin-1/EDNRA signaling
Apoptosis and survival_p53-dependent apoptosis
Development_EPO-induced PI3K/AKT pathway and Ca(2+) influx
Development_Osteopontin signaling in osteoclasts
Cell adhesion_Histamine H1 rec
Influence of low doses of Arsenite on glucose uptake in adipocytes
FGF signaling in pancreatic cancer
Neurophysiological process_Constitutive and regulated NMDA receptor trafficking
Development_TGF-beta-dependent induction of EMT via MAPK
Development_PDGF signaling via MAPK cascades
Tissue Factor signaling in cancer via PAR1 and PAR2
Development_TGF-beta-dependent induction of EMT via SMADs
Immune response_Gastrin in inflammatory response
Development_Hedgehog and PTH s
Development_Lipoxin inhibitory action on PDGF, EGF and LTD4 signaling
ENaC regulation in normal and CF airways
Development_WNT signaling pathway. Part 2
Membrane-bound ESR1: interaction with G-proteins signaling
Cytoskeleton remodeling_TGF, WNT and cytoskeletal remodeling
Immune response_Platelet activating factor/ PTAFR pathway signaling
Transcription_P53 signaling pathway
Translation _Regulation of EIF2 activity
FDR value
<0.05
0
10
20
-log10 (p-value)

F

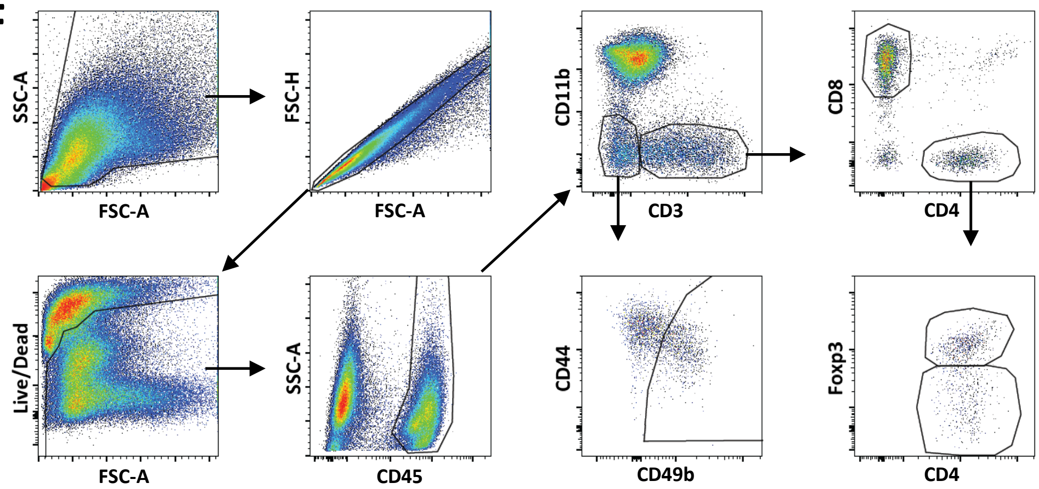

SSC-A
FSC-A
FSC-H
FSC-A
CD11b
CD3
CD8
CD4
Live/Dead
FSC-A
SSC-A
CD45
CD44
CD49b
Foxp3
CD4

Figure S6

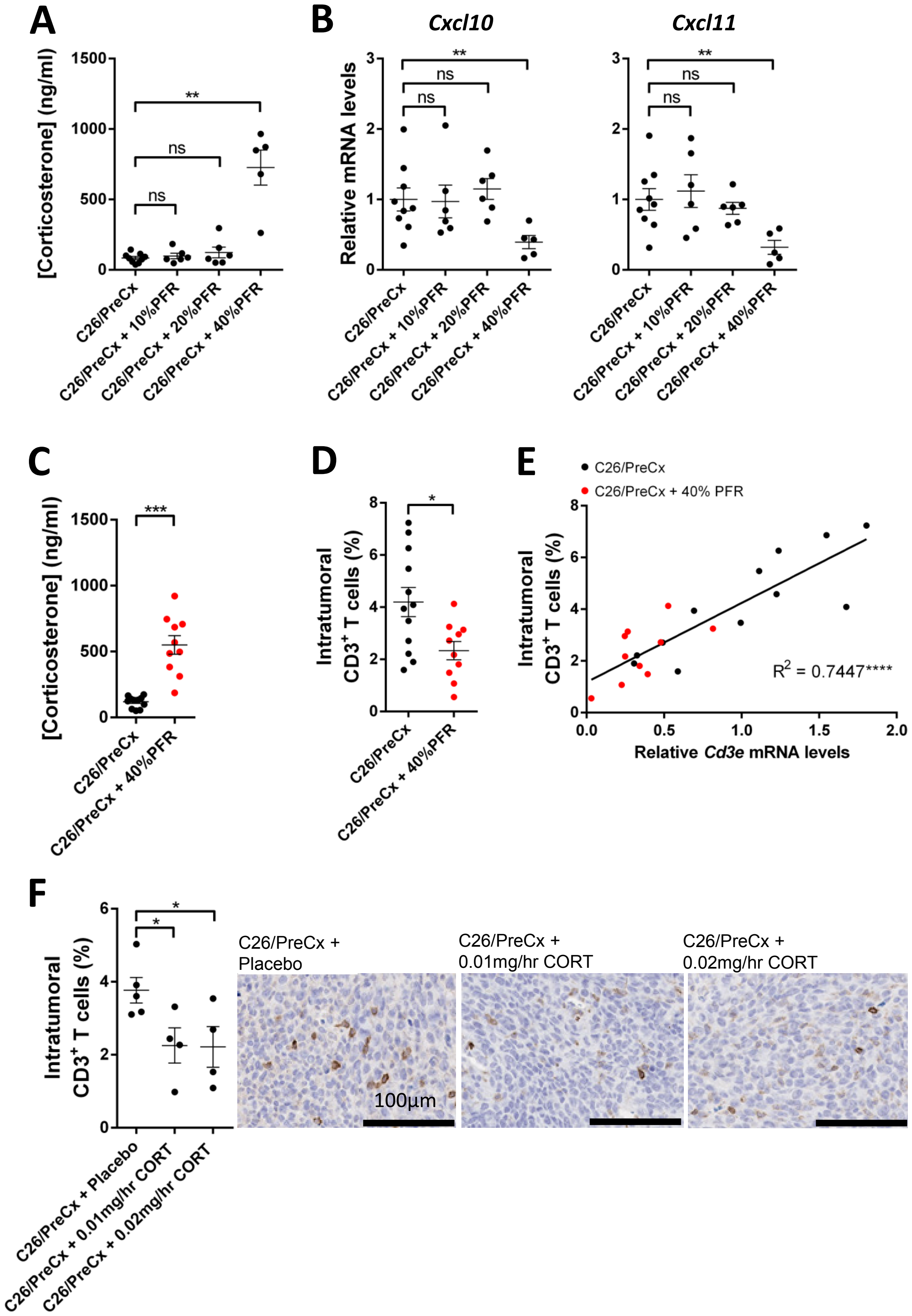
A
[Corticosterone] (ng/ml)
ns
**
C26/PreCx
C26/PreCx + 10%PFR
C26/PreCx + 20%PFR
C26/PreCx + 40%PFR
B
Cxcl10
Cxcl11
Relative mRNA levels
C
***
D
Intratumoral CD3+ T cells (%)
*
E
C26/PreCx
C26/PreCx + 40% PFR
R2 = 0.7447****
Relative Cd3e mRNA levels
F
C26/PreCx + Placebo
C26/PreCx + 0.01mg/hr CORT
C26/PreCx + 0.02mg/hr CORT
100µm

Figure S6

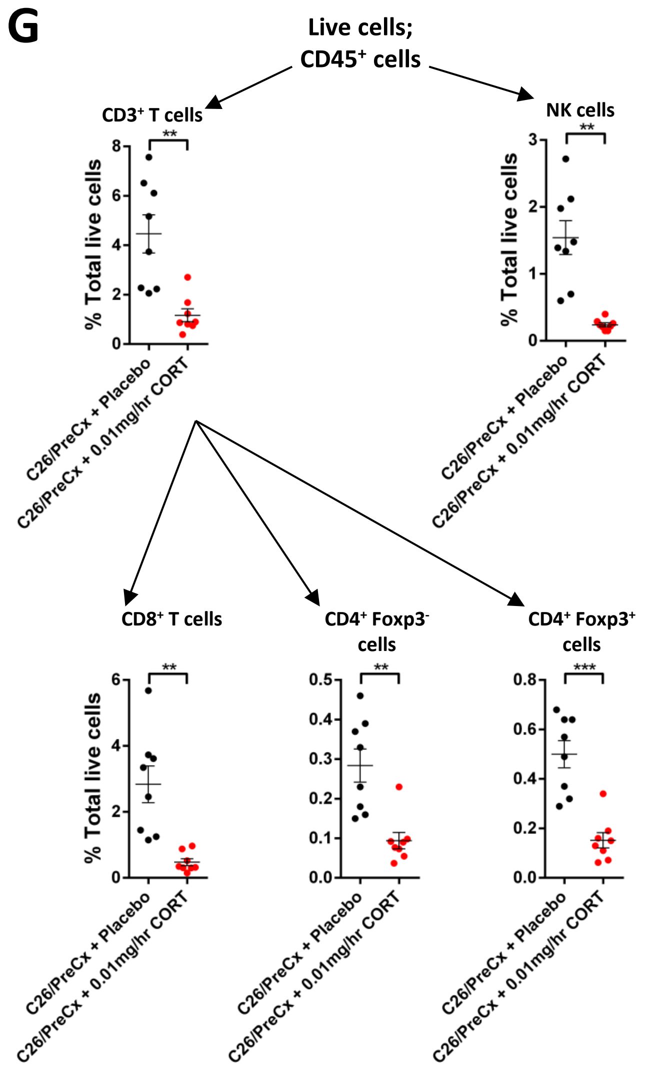
G
Live cells;
CD45+ cells
CD3+ T cells
NK cells
CD8+ T cells
CD4+ Foxp3- cells
CD4+ Foxp3+ cells
% Total live cells
**
***
C26/PreCx + Placebo
C26/PreCx + 0.01mg/hr CORT

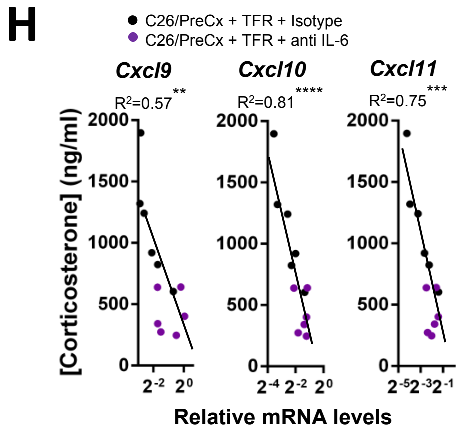
H
C26/PreCx + TFR + Isotype
C26/PreCx + TFR + anti IL-6
Cxcl9
R2=0.57 **
Cxcl10
R2=0.81 ****
Cxcl11
R2=0.75 ***
[Corticosterone] (ng/ml)
Relative mRNA levels

Figure S7

A

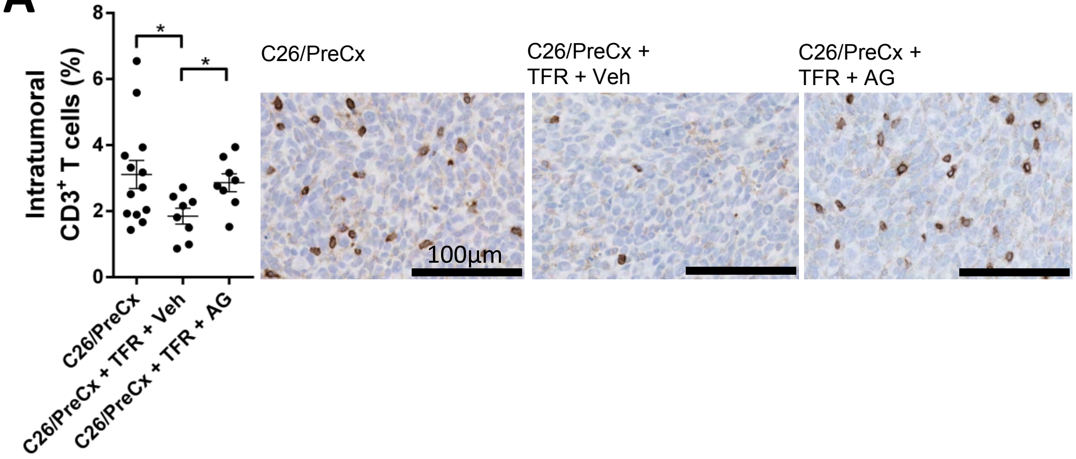

Intratumoral CD3+ T cells (%)
8
6
4
2
0
*
*
C26/PreCx
C26/PreCx + TFR + Veh
C26/PreCx + TFR + AG
C26/PreCx
C26/PreCx + TFR + Veh
C26/PreCx + TFR + AG
100μm

B

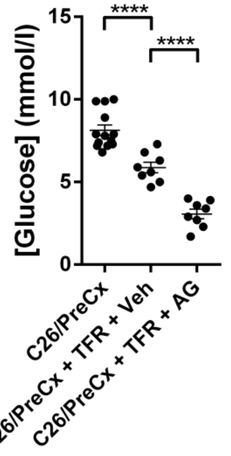

[Glucose] (mmol/l)
15
10
5
0
****
****
C26/PreCx
C26/PreCx + TFR + Veh
C26/PreCx + TFR + AG

Supplemental figure legends

Figure S1, related to Figure 1 and Figure 2: Phenotypic characterization of C26- and PDA-bearing mice, and extended human data. (A) A schematic illustration of pre-cachectic (/PreCx) and cachectic (/Cx) mice from the C26 and PDA model systems, and the respective non-tumor-bearing control groups, is presented. Weight-stable mice with established C26 or PDA tumors that were yet to develop cachexia were termed pre-cachectic (C26/PreCx and PDA/PreCx respectively). C26- and PDA-bearing mice were termed cachectic (C26/Cx and PDA/Cx respectively) following >5% body weight loss from their peak weight. (B) Body weight trajectories from individual C26/PreCx and C26/Cx mice are shown. (C) Representative photograph of masticated food pellets in a cage of a representative individually housed C26/Cx mouse. This phenomenon was not observed in the cages of C26/PreCx mice. The mass of the masticated food was taken into account when calculating the daily food intake for both C26 and PDA model systems. (D) Body composition was assessed via tissue wet weights in LM, C26/PreCx, and C26/Cx mice. (E) mRNA levels of genes involved in muscle catabolism were measured by RT-qPCR in the quadriceps of LM, C26/PreCx, and C26/Cx mice. (F) Body composition and (G) quadriceps mRNA levels were also assessed in PC, PDA/PreCx, and PDA/Cx mice. (H) Changes in body weight and (I) tissue wet weights caused by 24 hr TFR of LM and C26/PreCx mice were measured. Body weights were normalized to d14 post-tumor inoculation, and tissue wet weights were determined at d18. The (J) ages and (K) plasma ketone levels of pre-cachectic and cachectic patients with PDA whose weight changes and serum cortisol levels were presented in Fig. 1F, G are shown. The comparisons in (D-G) were performed using one-way ANOVA with Tukey's correction for post-hoc testing. LM vs C26/PreCx, LM + TFR vs C26/PreCx + TFR, and non-cachectic vs cachectic cancer patient data comparisons in (H-K) were performed using two-tailed t-tests with Welch's correction. *p<0.05, **p<0.01, ***p<0.001, ****p<0.0001. Data are presented as mean ±SEM.

Figure S2, related to Figure 1 and Figure 2: Extended characterization of the response to spontaneous and induced food restriction in mice bearing C26 and PDA tumors, respectively. (A) Insulin and (B) leptin plasma levels were measured from terminal blood samples. Dotted lines represent assay detection limits. (C, D) Blood ketone, blood glucose, and plasma corticosterone profiles in C26/PreCx and LM mice were determined 18-30 hrs post-initiation of TFR via tail vein bleeds (n=10 per group). (E) Sodium octanoate was administered to PC and PDA/PreCx mice 24 hrs post-initiation of TFR, and blood glucose levels were measured (n=7-8 per group). Data from LM, C26/PreCx, and C26/Cx mice, and PC, PDA/PreCx, and PDA/Cx mice, were compared using one-way ANOVA with Tukey's correction for post-hoc comparisons. The LM + TFR vs C26/PreCx + TFR, and PC + TFR vs PDA/PreCx + TFR data comparisons in (A, B) as well as data comparisons at each timepoint in (C-E) were performed using two-tailed t-tests with Welch's correction. Additional within-group comparisons of the data from the timecourses in (C, D) were performed using two-tailed ratio paired t-tests of both groups at each timepoint relative to their 18hrs post-TFR measurements (#).* $p<0.05$; ** $p<0.01$; *** $p<0.001$; ****$p<0.0001$. Data are presented as mean +/- SEM.

Figure S3, related to Figure 3: Extended data from plasma cytokine and chemokine screens, and effects of IL-6 infusion on additional metabolic parameters. (A-C) Multi-cytokine array analysis was performed using terminal bleed samples from LM, C26/PreCx , C26/Cx, LM + TFR and C26/PreCx + TFR mice, as well as PC, PDA/PreCx and PDA/Cx mice. Dotted lines represent assay detection limits for the respective target cytokines. (D) Body weight kinetics (n=8 per group) and (E) tissue wet weights at dissection were determined in non-tumor-bearing LM mice after 72 hrs of 0.3µg/hr IL-6 infusion, with TFR during the final 24 hrs. (F) Tail bleed corticosterone levels after 48 hrs of 0.3µg/hr IL-6 infusion and before TFR were determined in LM mice. Data from (A) LM, C26/PreCx, and C26/Cx mice, and (B) PC, PDA/PreCx, and PDA/Cx mice were compared using one-way ANOVA with Tukey's correction for post-hoc comparisons. All other data comparisons as well as comparisons of data at each timepoint in (D) were performed using two-tailed t-tests with Welch's correction. * $p<0.05$; ** $p<0.01$; *** $p<0.001$; **** $p<0.001$. Data are presented as mean +/- SEM.

Figure S4, related to Figure 4: Additional effects of neutralization of IL-6 in C26/PreCx + TFR mice. Mice receiving anti-IL-6 or isotype control antibody were assessed for (A) changes in body weight (n=6 per group), and (B, C) tumor volume. (D) The food intake during the 48 hrs of antibody exposure prior to TFR was measured. Following TFR, which occurred during the final 24 hrs of the experiment, (E) tissue wet weights and terminal bleed plasma levels of (F) ALT and (G) bilirubin were assessed. C26/PreCx + TFR + isotype vs C26/PreCx + TFR + anti-IL-6 data comparisons, including data comparisons at each timepoint in (A), were performed via two-tailed t-tests with Welch's correction. Data are presented as mean ±SEM.

Figure S5, related to Figure 5: Extended RNA-seq and flow cytometry data from tumors taken from C26/PreCx and C26/Cx mice. (A) Principal Components Analysis of RNA-seq data from tumor lysates from C26/PreCx and C26/Cx mice. The first component (PC1) accounted for 45.0% of the variance and separated the two groups. (B) A plot of differential gene expression in tumors from C26/Cx relative to C26/PreCx mice is displayed. The intensity of the blue area represents density, green dots represent changes that were significant at a false discovery rate (FDR) <0.05, red dots represent changes significant at FDR<0.01, and $\log_2$FC is $\log_2$(fold change of C26/Cx transcripts relative to C26/PreCx). (C, E) The significantly down- and up-regulated gene subsets in tumors from C26/Cx relative to C26/PreCx mice, totalling 66.2% and 33.8% of the differentially expressed gene population respectively, were subjected to MetaCore enrichment analysis. The pathways were ranked by $-\log_{10}$ (p-value) and the top 30 pathways for each subset are displayed. (D) $CD3^+$ T cell percentages from C26/PreCx and C26/Cx mice were quantified via IHC. (F) Tumors from an independent cohort of C26/PreCx and C26/Cx mice were subjected to flow cytometric analysis using markers for lymphoid cells. Data comparisons in (D) were performed using two-tailed t-tests with Welch's correction. **$p<0.01$. Data are presented as mean ±SEM.

Figure S6, related to Figure 6: Effects of partial food restriction, corticosterone infusion, and anti-IL-6 treatment on intratumoral immunity. (A, B) C26/PreCx mice were subjected to 3 days of partial food restriction (PFR) by 10, 20, and 40% relative to baseline. (A) Corticosterone levels were determined from tail bleed samples, and (B) mRNA levels of both *Cxcl10* and *Cxcl11* were determined by RT-qPCR from C26 tumor lysates. In freely feeding C26/PreCx mice and C26/PreCx mice that were subjected to 3 days of 40% PFR, (C) plasma corticosterone levels via tail bleeds, (D) tumoral $CD3^+$ T cell percentages via IHC of whole-tumor sections, and (E) the correlation between $CD3^+$ T cell percentages (as assessed by IHC) and relative *Cd3e* mRNA levels (as assessed by RT-qPCR) were determined (n=10-12 per group). (F) C26-bearing mice were subjected to 0.01mg/hr or 0.02mg/hr corticosterone infusions or placebo control, and $CD3^+$ T cell percentages were determined via IHC. (G) Additional C26-bearing mice were subjected to 0.01mg/hr corticosterone infusion or placebo control, and lymphoid populations were quantified using flow cytometry. (H) C26/PreCx mice were administered isotype control or neutralizing anti-IL-6 antibodies 48 hrs prior to TFR and when TFR was initiated. The tumoral mRNA levels for the CXCR3-related chemokines were measured by RT-qPCR, logarithmically transformed, and plotted versus the terminal bleed plasma corticosterone levels. Data in (E, H) were analysed by linear regression. Data in (F) were compared using one way ANOVA with Fisher's LSD test for post-hoc comparisons. All other indicated data comparisons were performed using two-tailed t-tests with Welch's correction. * $p<0.05$; **$p<0.01$; ***$p<0.001$; ****$p<0.0001$. Data are presented as mean +/- SEM.

Figure S7, related to Figure 6: Extended data from aminoglutethimide-treated C26/PreCx + TFR mice. C26/PreCx mice were administered aminoglutethimide or vehicle control at 0hr, 8hr, and 16hrs post-TFR and sacrificed 24hrs post-TFR. (A) Intratumoral $CD3^+$ T cell percentages were determined via IHC of whole-tumor sections, and (B) blood glucose levels were measured via tail bleed at 24hrs post-TFR. All indicated comparisons were performed using two-tailed t-tests with Welch's correction. * $p<0.05$; ** $p<0.01$; *** $p<0.001$; **** $p<0.0001$. Data are presented as mean ±SEM. Veh = vehicle; AG = aminoglutethimide.

Table S1, related to Figure 5: Selected differentially expressed genes in tumors from pre-cachectic and cachectic C26 bearing mice.

	(Protein)	Gene	Mean (C26)	Mean (C26/Cx)	Fold Change	p-value (adj.)
**Epithelial Cells**		*Cdh1*	31.24	62.64	2.005	8.49E-03
		Krt20	8475.04	14107.02	1.665	8.86E-05
**Fibroblasts**		*Fap*	677.62	828.98	1.223	1.81E-01
	(aSMA)	*Acta2*	113.93	48.48	0.426	8.14E-06
		Col1a1	16606.91	11264.02	0.678	5.31E-03
		Col1a2	28152.49	24170.74	0.859	1.35E-01
		Col4a1	8504.55	12754.90	1.500	4.87E-05
		Col4a2	5960.03	9566.37	1.605	1.22E-05
**Leucocytes**	(CD45)	*Ptprc*	3161.28	1213.74	0.384	4.11E-09
**Lymphoid Cells**		*Cd3e*	57.09	17.53	0.307	1.08E-03
		Cd4	69.13	26.99	0.390	1.37E-04
		Cd8a	117.38	36.19	0.308	2.73E-04
		Cd8b	36.81	10.09	0.274	1.03E-03
		Klrb1b	178.73	74.75	0.418	4.79E-12
		Klrb1c	48.17	18.99	0.394	1.97E-07
		Gzma	144.82	24.97	0.172	5.29E-04
		Gzmb	258.12	77.72	0.301	9.14E-04
		Prf1	156.53	65.85	0.421	2.62E-05
		Foxp3	14.56	9.85	0.677	6.02E-02
	(CD25)	*Il2ra*	30.96	21.97	0.710	6.62E-02
		Ifng	12.54	2.02	0.161	3.84E-03
	(T-bet)	*Tbx21*	13.59	4.38	0.322	7.89E-05
		Eomes	37.58	23.67	0.630	6.61E-05
	(PD-1)	*Pdcd1*	50.99	20.08	0.394	2.20E-04
**Myeloid Cells**	(CD11b)	*Itgam*	2901.61	1697.48	0.585	1.32E-09
	(CD11c)	*Itgax*	2135.56	1223.86	0.573	1.45E-12
	(MHC II)	*Cd74*	15713.18	5582.11	0.355	2.67E-08
		Cd40	70.24	20.57	0.293	2.26E-08
		Cd68	1212.43	679.46	0.560	<1.21E-13
	(F4/80)	*Emr1*	1427.34	545.88	0.382	8.32E-12
		Ly6c1	328.85	139.20	0.423	3.09E-04
		Ly6c2	105.67	15.81	0.150	5.35E-04
		Ly6g	0.11	0.47	4.112	4.43E-01
		Ccr2	825.30	293.64	0.356	2.61E-09
**Endothelial Cells**	(CD31)	*Pecam1*	964.03	554.18	0.575	6.63E-08
		Vwf	78.15	104.92	1.342	1.09E-01
	(eNOS)	*Nos3*	38.47	34.22	0.889	6.86E-01
**Chemokines**		*Cxcl9*	1384.42	298.51	0.216	<1.21E-13
		Cxcl10	473.52	112.15	0.237	6.27E-07
		Cxcl11	68.17	12.57	0.184	2.91E-06
		Cxcl12	187.27	81.07	0.433	1.56E-03
		Ccl2	1676.61	757.83	0.452	1.91E-03
		Ccl5	133.98	37.68	0.281	4.64E-06
**Growth Factors**	(M-CSF)	*Csf1*	2034.25	1264.97	0.622	8.46E-07
	(GM-CSF)	*Csf2*	1.13	1.03	0.913	9.98E-01
	(G-CSF)	*Csf3*	10.37	33.14	3.196	3.49E-01

Experimental procedures

Animal Experiments

All experiments were performed in accordance with national and institutional guidelines and were approved by the UK Home Office, the animal ethics committee of Cancer Research UK Cambridge Institute, and the University of Cambridge. The C26 model experiments were performed on wild-type male Balb/c mice purchased from Charles River Laboratories and kept on a 24hr 12:12 light-dark cycle. For tumor experiments, cells from the C26 cell line were cultured in RPMI 1640 Medium (+L-Glutamine) with 10% heat-inactivated FBS under sterile conditions. This was followed by non-enzymatic dissociation (Gibco), resuspension in FBS-free RPMI, counting of viable cell concentration using a Vi-Cell counter and injection of $2*10^6$ viable cells subcutaneously into the flank of each mouse, in an RPMI vehicle at 100µl per mouse, at between 8-10 weeks of age. All mice were singly housed on blotting paper bedding post-inoculation so as to enable quantification of food spillover as part of food intake measurements (See Fig. S1C).

C26-bearing mice were termed pre-cachectic from 18 days post-inoculation onwards. Cachexia, defined as >5% loss of body weight from peak body weight, occurred in these mice at 22-30 days post-inoculation. The 24hr fasted pre-cachectic C26-bearing mice (C26/PreCx + TFR) were enrolled in experiments at day 18 post-injection, contingent on prior weight stability, and fasted at 1200h which was the middle of the 12hr daily light period. Only pre-cachectic C26 and littermate control (LM) mice weighing 23-28g at the point of food restriction were enrolled in food restriction experiments. All tumors from experimentally enrolled C26/PreCx + TFR mice weighed 0.7-1.5g upon dissection, were fully located in the flank, and had no macroscopic evidence of ulcerations. C26-bearing mice in all statistical comparisons were matched regarding age and tumor incubation time, and C26-bearers undergoing therapeutic reversal with anti-IL-6 or aminoglutethimide were, in addition, stratified by body weight prior to randomisation to either treatment or control study arms. Anti-IL-6 (MP5.20F3) and isotype IgG1 (HRPN) was purchased from BioXcell, diluted in PBS and injected i.p. at 1.25mg per mouse 48hrs prior to and again at the point of TFR. For the octanoate challenge, sodium octanoate (Sigma) was dissolved to 200mM in 0.9% NaCl and injected i.p. at 6ml/kg in 24hr fasted mice. For aminoglutethimide experiments, aminoglutethimide was dissolved in a PBS vehicle containing 9% DMSO, 1% Tween, and 30% PEG-400 by volume. Aminoglutethimide was administered to pre-cachectic C26-bearing mice at 37.5mg/kg at 0hr, 8hr, and 16hr post-food restriction, with sacrifice 24hrs post-food restriction. Mild sedation and ataxia were observed in some treated mice. For partial food restriction (PFR) experiments, C26-bearing mice were singly housed day 10 post-inoculation and a 7-day baseline food intake was determined for each mouse following a 24hr acclimatization period. Mice were then stratified primarily by baseline food intake and secondarily by body weight, food restricted by 10, 20 or 40% from their individual baselines and fed at 1700h on days 17, 18, and 19 before being sacrificed at 1300h on day 20. For the continuous corticosterone infusion experiments, C26-bearing mice were anaesthetized with isoflurane and subcutaneously implanted with one placebo or one or two 5mg corticosterone pellets (Innovative Research of America) designed to release their content over 21 days. Pellet implantation was initiated at day 13 post-tumor inoculation.

For the IL-6 infusion in non-tumor bearing littermate mice, recombinant carrier-free murine IL-6 was purchased from BioLegend, diluted in PBS, loaded into osmotic minipumps (ALZET model 1003D, which elutes at 1.0µl/hr) at 0.3, 0.1, and 0.03mg/ml, and inserted into the subcutaneous space of singly housed 12 week old male Balb/c mice. The pumps were left to elute for 72hrs with the final 24hrs under TFR. The PBS-infused male Balb/c control group was matched for age and mice were stratified by body weight prior to enrolment into the experimental groups. TFR was initiated in the middle of the 12hr light period at 1200h, and mice were sacrificed after 24hrs of TFR. At this point, some mice were approaching the clinical severity limit.

KPC mice and PC littermates were of various age and male and female mice were enrolled. All experimental groups were randomised for sex and age. Pancreatic ductal adenocarcinoma (PDA) tumors in KPC mice were detected via palpation and high-resolution ultrasound scans (Vevo 2100, VisualSonics), and confirmed at necropsy. All tumours

from treatment trials were confirmed histopathologically as adenocarcinomata. Only weight-stable, PDA-bearing mice with tumors with maximum diameters >4mm (measured by ultrasound) were enrolled in experiments, and these mice were all termed pre-cachectic at the point of enrolment. Pre-cachectic PDA-bearing mice were monitored for body weight and food intake on a daily basis, and were termed cachectic following weight loss of more than 5% from their peak recorded weight. Excluding those PDA-bearing mice that succumbed to ascites, 85% of the PDA-bearing mice that were enrolled as pre-cachectic subsequently developed cachexia over a period of 30 days post enrolment. KPC mice that were found to have macroscopic liver metastases, severe ascites, diarrhoea or bowel obstruction at or before necropsy were excluded from the analyses.

To determine the effects of corticosterone pellets +/- immune therapy on PDA tumors mice, pre-cachectic PDA-bearing KPC mice with average maximum tumor diameters between 4-7mm (measured via ultrasound) and good clinical condition were enrolled at d-2 for treatment studies. The treatment was started at d0 and tumour size was measured using ultrasound scans on d3 and d6. Where possible, tumors were assessed at multiple angles and the average volumes were determined for each timepoint. Subcutaneous insertion of corticosterone or placebo pellets and/or osmotic pumps (ALZET model 1007D containing a 0.22µm filtered 90mg/ml solution of AMD3100) in KPC mice was performed on d0, with anti-PD-L1 (10F.9G2; BioXcell) or isotype (LTF-2; BioXcell) therapy at 0.2mg/mouse on d0, 2 and 4 post-surgery via i.p. injection.

Euthanasia was performed within the 12hr light period between 1200-1400h unless otherwise stated. Terminal bleeds were obtained through exanguination via cardiac puncture under isofluorane anaesthesia and death was confirmed by cervical dislocation. Heparin was used as anti-coagulant for plasma collection. Tail vein bleeds were performed using venesection before anaesthesia and without restraint and blood was collected into heparinized BRAND micro haematocrit capillary tubes (Sigma). Samples were kept on ice at all time. Plasma was prepared by centrifugation at 14,000g for 5 minutes at 4^0C and snap frozen in liquid nitrogen. Organs and other tissues were rapidly dissected in a consistent order within 3 minutes, snap frozen in liquid nitrogen and stored at -80^0C. Aliquots of tumor samples were fixed in 10% neutral buffered formaldehyde for 24hrs at room temperature before being transferred to 70% ethanol and processed for immunohistochemistry.

Murine Blood & Plasma Measurements

Tail bleeds (whole blood) acquired via tail venesection using a scalpel were analysed for glucose and ketone concentrations using Aviva Accu-Check glucometers (Roche) and Freestyle Optium Neo ketometers (Abbott laboratories) respectively. Mice were not restrained for tail bleeds. Corticosterone from heparinized plasma prepared from tail bleeds (collected using heparinized BRAND micro haematocrit capillary tubes) or terminal cardiac bleeds (acquired using a syringe flushed with heparin solution in PBS) where indicated was quantified using either the IBL or the IDS ELISA. The assay provider was switched from IDS to IBL due to IDS discontinuing their ELISA as data for this manuscript were being collected. The sample incubation step from the IBL assay protocol was 3hrs at room temperature so as to reach displacement equilibrium as determined by preliminary data (not shown). The IBL assay was used for the measurements of corticosterone in the following figure panels: 3F; 6B, D; 7C; S2D; S3F; S6C. All other corticosterone assays in this manuscript were performed using the IDS assay. Terminal cardiac bleed plasma glucose levels were assessed using an automated assay on the Siemens Dimension RxL analyser, and terminal bleed plasma ketone levels were assessed using the Stanbio Beta Hydroxybutyrate Liquicolour kit. Insulin, leptin and cytokines for the multi-cytokine array were measured using Meso Scale Discovery kits. The IL-6 levels for Fig. 3C were measured using R&D's Quantikine ELISA for murine IL-6.

AMD3100 was extracted from plasma samples by protein precipitation with methanol of samples pre spiked with internal standard (D_4 labelled AMD3100) and EDTA. Quantitation was by LC-MS/MS over the calibration range 50-5000 ng/mL. LC separation was achieved on a Phenomenex Kinetex 2.6µm Bipehenyl 100x2.1mm using an optimised gradient with 0.1 % formic acid in methanol and water using an Accela UHPLC autosampler and pump.

Electro spray in the positive ionisation mode monitoring the mass transitions 252-420 for AMD3100 and 254-424 for the deuterium labelled internal standard was performed on a Thermo TSQ Vantage.

RT-qPCR

mRNA was extracted from frozen tissues using TRIzol Reagent (Invitrogen) following the manufacture's protocol. The concentration and purity of aqueous RNA from each preparation was assessed using an ND1000 spectrophotometer. mRNA templates were diluted to 2ng/µl (muscle and liver) or 30ng/µl (tumor) and mRNA was analyzed by quantitative real-time PCR using the TaqMan RNA-to-Ct 1-Step Kit (Life Technologies). The detection threshold for all samples including controls in a given run was manually adjusted so that it lay within the exponential phase of product amplification. mRNA levels were normalized to either *Rn18s* (liver and tumor) or *Tbp* (quadriceps) using the ddCt method. Tumor lysate *Rn18s* values were measured on a 1 in 10 dilution of the template to ensure that the results for this parameter lay within the log-linear range of the standard curve. The following TaqMan gene expression assays were used: Mm01277044_m1 (*Tbp*); Mm03928990_g1 (*Rn18s*); Mm00440939_m1 (*Ppara*); Mm01323360_g1 (*Acadm*); Mm00550050_m1 (*Hmgcs2*); Mm00499523_m1 (*Fbxo32*); Mm01185221_m1 (*Trim63*); Mm00434946_m1 (*Cxcl9*); Mm99999072_m1 (*Cxcl10*); Mm00444662_m1 (C*xcl11*); Mm00599683_m1 (*Cd3e*); Mm01182108_m1 (*Cd8a*); Mm01168134_m1 (*Ifng*); Mm00442834_m1 (*Gzmb*); Mm00812512_m1 (*Prf1*)

RNA-sequencing

RNA extracted from frozen tissues via TRIzol was run through Qiagen RNeasy columns following the "RNA cleanup" protocol. Integrity was confirmed using RIN values (Agilent) with a cutoff of 8, and libraries were prepared using the Illumina TruSeq mRNA Stranded Sample prep kit (96 index High Throughput) using 12 rounds of PCR. Libraries were quantity checked and normalized using Kapa Biosystem's Library Quantification Kit. Library Quality was assessed using Agilent Bioanalyser DNA high sensitivity kits. The final pooled library was run on a MiSeq to assess final sequencing quality before HiSeq 2500 V4 single end 50bp sequencing. We aimed to generate 10-20M single-end 50bp reads per sample.

For the analysis, single-end 50bp reads were aligned to the mouse genome version GRCm38.74 using TopHat v2.0.4 (Trapnell et al., 2009). Read counts were obtained using HTSeq-count v0.5.3p9 (http://www-huber.embl.de/users/anders/HTSeq/doc/overview.html). Read counts were normalized and tested for differential gene expression using the Bioconductor package DESeq v1.10.1 (Anders and Huber, 2010). Multiple testing correction was applied using the Benjamini-Hochberg method. GSEA was performed by ranking all genes tested in RNA-Seq using $-\log_{10}$ (p-values) derived from differential expression analyses and testing against MSigDB Canonical Pathways (C2:CP). Metacore enrichment analyses gated on the significantly (FDR<0.05) up- and down-regulated genes were also performed using Metacore's pathway maps database.

Immunohistochemistry

Tissues were fixed in 10% neutral buffered formaldehyde for 24hrs at room temperature before transferral to 70% ethanol and embedding into paraffin wax. All sections were cut at 3µm and dried at 60°C for 1 hr. Immunohistochemistry was performed for CD3 using a Leica Bond III immunostainer (Leica, Milton Keynes, UK). Deparaffinisation and rehydration was conducted on a Leica ST5020 before transferring to the Bond III where antigen retrieval was performed using Tris EDTA, pH9 (ER2) for 20 mins at 100 °C, incubation in a rabbit polyclonal anti-CD3 antibody (A0452) for 15 mins, HRP-linked anti-rabbit polymer for 8 mins, diaminobenzidine for 10 mins and DAB Enhancer (Leica) for 10 mins. Counterstaining was with Haematoxylin (Leica) for 2 mins. All incubations were performed at room temperature and all incubation steps were followed with 3 washed in TBS (Bondwash, Leica). Following application of cover slips and drying, the slides were scanned at 20x on a Leica AT2 and subsequently analysed in a blinded manner using the Cytonuclear v1.4 algorithm on the HALO platform (Indica Labs).

Flow Cytometry

C26 tumors were dissected and placed in ice-cold PBS. Portions of each tumor were then minced using razor blades, and placed into a ice-cold digestion buffer containing RPMI with 1mg/ml collagenase (Sigma C0130) and 0.1mg/ml DNAse (Sigma D4527). The suspensions were warmed to 37 ^{0}C and agitated at 750rpm for 60 minutes using an Eppendorf Thermomixer Comfort. The suspensions were triturated for 5 seconds at the 0, 20, 40, and 60 minute timepoints during the agitation period. At 60 minutes, EDTA was added to the suspension to a final concentration of 10mmol/l, and 5 minutes after that the suspension was pipetted up and down before being passed though at 70µm cell strainer. 5 volumes of flow cytometry staining buffer (PBS containing 5% heat-inactivated FBS, 0.05% sodium azide, and 2mmol/l EDTA) were added through the filter at this point and the resulting single-cell suspension was kept on ice. Cells were stained for flow cytometry according to reagent manufacturers' protocols. Viability was determined using eBioscience e780 fixable viability dye at a 1/1000 dilution. The following antibodies were used to identify immune cell subsets: anti-CD45 (BUV395; 30-F11; 0.5µg/ml; BD); anti-CD3e (PE; 145-2C11; 1µg/ml; Biolegend); anti-CD4 (BV605; GK1.5; 1µg/ml; Biolegend); anti-CD8a (FITC; 53-6.7; 2.5µg/ml; Biolegend); anti-CD49b (PE-Cy7; DX5; 2µg/ml; Biolegend); anti-CD11b (BV786; M1/70; 0.5µg/ml; Biolegend); anti-Foxp3 (APC; FJK-16s; 3µg/ml; eBioscience); anti-CD44 (PerCP/Cy5.5; IM7; 0.2µg/ml; Biolegend). CD49b positivity was determined using a fluorescence-minus-one control. Anti-CD16/32 (Unconjugated; 2.4G2; 5 µg/ml; BD) was used for Fc-blocking prior to antibody staining. The eBioscience Foxp3/Transcription Factor Fixation/Permeabilization Concentrate and Diluent was used prior to Foxp3 staining. Sample analysis was performed using an LSR II cytometer. At least 200,000 events per tumor sample were collected. Data were subsequently analysed using FlowJo.

Human studies

The study and the sample acquisition were performed in concordance with local and national guidelines. Patients at least 18 years old with histologically confirmed pancreatic adenocarcinoma were recruited in the outpatient department of the Cambridge University Hospital NHS Foundation Trust as part of the CAMPAN study. Important exclusion criteria were confirmed second malignancies and previous steroid therapy other than anti-emetic therapy with steroids more than 14 days preceding sample acquisition. All participants provided written informed consent. All samples were taken between 1300h and 1500h. For serum acquisition, blood samples were spun at 14000rpm in a table top centrifuge for 5 min after 15min of coagulation. Plasma was obtained using heparin as anti-coagulant with the same centrifugation protocol. Plasma and serum samples were snap frozen in aliquots. Patients in both study cohorts were matched with regard to age, sex, and treatment history. Patients were designated cachectic if they met the international consensus definition for cachexia i.e. if they exhibited weight loss of >5% over past 6 months (in the absence of simple starvation) or BMI <20 and ongoing weight loss of more than 2% (Fearon et al., 2011). Serum cortisol levels were assessed using the Cortisol Parameter Assay Kit from R&D systems. Serum IL-6 levels were assessed using the Human IL-6 Quantikine ELISA from R&D systems. Ketone levels from plasma were assessed using Freestyle Optium Neo ketometers (Abbott laboratories).

Statistical Analysis

Statistical analyses, unless otherwise indicated, were performed using GraphPad Prism 6.

References

Anders, S., and Huber, W. (2010). Differential expression analysis for sequence count data. Genome Biol. *11*, R106.

Fearon, K., Strasser, F., Anker, S.D., Bosaeus, I., Bruera, E., Fainsinger, R.L., Jatoi, A., Loprinzi, C., MacDonald, N., Mantovani, G., et al. (2011). Definition and classification of cancer cachexia: an international consensus. Lancet Oncol. *12*, 489–495.

Trapnell, C., Pachter, L., and Salzberg, S.L. (2009). TopHat: discovering splice junctions with RNA-Seq. Bioinformatics *25*, 1105–1111.
